# Bean Extract-Based Gargle for Efficient Diagnosis of Active COVID-19 Infection Using Rapid Antigen Tests

**DOI:** 10.1128/spectrum.01614-21

**Published:** 2022-02-16

**Authors:** Joseph Kwon, Euna Ko, Se-Young Cho, Young-Ho Lee, Sangmi Jun, Kyuhong Lee, Eunha Hwang, Bipin Vaidya, Jeong-Hwan Hwang, Joo-Hee Hwang, Namsu Kim, Mi-Kyung Song, Hye-Yeon Kim, Dai Ito, Yuxi Lin, Eunae Jo, Kyeong Eun Yang, Hee-Chung Chung, Soyoung Cha, Dong Im Kim, Yoon-Sun Yi, Sung-Ho Yun, Sun Cheol Park, Sangmin Lee, Jong-Soon Choi, Dal Sik Kim, Duwoon Kim

**Affiliations:** a Department of BioChemical Analysis, Korea Basic Science Institute, Daejeon, Republic of Korea; b Bio-Analytical Science, University of Science and Technology, Daejeon, Republic of Korea; c BIO3S, Inc., Gwangju, Republic of Korea; d Research Center for Materials Analysis, Korea Basic Science Institute, Daejeon, Republic of Korea; e Department of Food Science and Technology, Foodborne Virus Research Center, Chonnam National University, Gwangju, Republic of Korea; f Graduate School of Analytical Science and Technology, Chungnam National University, Daejeon, Republic of Korea; g Research Center for Bioconvergence Analysis, Korea Basic Science Institute, Daejeon, Republic of Korea; h National Center for Efficacy Evaluation for Respiratory Disease Product, Korea Institute of Toxicology, Daejeon, Republic of Korea; i Department of Human and Environmental Toxicology, University of Science and Technology, Daejeon, Republic of Korea; j Department of Internal Medicine, Research Institute of Clinical Medicine of Jeonbuk National University-Biomedical Research Institute of Jeonbuk National University Hospital, Jeonbuk National University Medical School and Hospital, Jeonju, Republic of Korea; k Department of Laboratory Medicine, Research Institute of Clinical Medicine of Jeonbuk National University-Biomedical Research Institute of Jeonbuk National University Hospital, Jeonbuk National University Medical School and Hospital, Jeonju, Republic of Korea; l Center for Convergent Research of Emerging Virus Infection, Korea Research Institute of Chemical Technology, Daejeon, Republic of Korea; m Department of Brain and Cognitive Science, Institute of Science and Technology (DGIST), Daegu, Republic of Korea; n BioApplications Inc., Pohang, Republic of Korea; University of Prince Edward Island

**Keywords:** COVID-19, oral virus, rapid diagnostic test, SARS-CoV-2, sensitivity, specificity

## Abstract

The antigen-based rapid diagnostic test (Ag-RDT) using saliva specimens is fast, noninvasive, and suitable for SARS-CoV-2 self-testing, unlike nasopharyngeal swab (NPS) testing. We evaluated a novel Beanguard gargle (BG)-based virus collection method that can be applied to Ag-RDT as an alternative to the current RT-PCR with an NPS for early diagnosis of COVID-19. This clinical trial comprised 102 COVID-19-positive patients hospitalized after a governmental screening process and 100 healthy individuals. Paired NPS and BG-based saliva specimens from COVID-19 patients and healthy individuals were analyzed using NPS-RT-PCR, BG-RT-PCR, and BG-Ag-RDTs, whose diagnostic performance for detecting SARS-CoV-2 was compared. BG-Ag-RDTs showed high sensitivity (97.8%) and specificity (100%) in 45 patients within 6 days of illness and detected all cases of SARS-CoV-2 Alpha and Delta variants. In 11 asymptomatic active COVID-19 cases, both BG-Ag-RDTs and BG-RT-PCR showed sensitivities and specificities of 100%. Sensitivities of BG-Ag-RDT and BG-RT-PCR toward salivary viral detection were highly concordant, with no discrimination between symptomatic (97.0%), asymptomatic (100%), or SARS-CoV-2 variant (100%) cases. The intermolecular interactions between SARS-CoV-2 spike proteins and truncated canavalin, an active ingredient from the bean extract (BE), were observed in terms of physicochemical properties. The detachment of the SARS-CoV-2 receptor-binding domain from hACE2 increased as the BE concentration increased, allowing the release of the virus from hACE2 for early diagnosis. Using BG-based saliva specimens remarkably enhances the Ag-RDT diagnostic performance as an alternative to NPS and enables noninvasive, rapid, and accurate COVID-19 self-testing and mass screening, supporting efficient COVID-19 management.

**IMPORTANCE** An Ag-RDT is less likely to be accepted as an initial test method for early diagnosis owing to its low sensitivity. However, our self-collection method, Ag-RDT using BG-based saliva specimens, showed significantly enhanced detection sensitivity and specificity toward SARS-CoV-2 including the Alpha and Delta variants in all patients tested within 6 days of illness. The method represents an attractive alternative to nasopharyngeal swabs for the early diagnosis of symptomatic and asymptomatic COVID-19 cases. The evidence suggests that the method could have a potential for mass screening and monitoring of COVID-19 cases.

## INTRODUCTION

The prevalence of coronavirus disease-2019 (COVID-19) caused by severe acute respiratory syndrome coronavirus-2 (SARS-CoV-2) has significantly altered daily living and rapidly become a global health threat (1) with variants spreading across the world (2). Therefore, accurate identification and rapid diagnosis of SARS-CoV-2 is critical for lowering continued transmission and gaining control over the current pandemic.

RT-PCR-based quantification of SARS-CoV-2 RNA obtained from the upper respiratory tract via nasopharyngeal swabs (NPSs) has been universally adopted as the reference standard for viral detection (3). Although this method offers high sensitivity and specificity for SARS-CoV-2 detection, it requires skilled professionals and sophisticated instrumentation, as well as a long period for detection (4). Therefore, a rapid and improved self-diagnostic strategy is required to facilitate early detection and prevent COVID-19 spread.

Antigen detection using lateral flow-based rapid diagnostic tests (known as Ag-RDTs) are widely used to provide on-site diagnosis and mass screening for the early detection of pathogens (5) before them becoming a significant risk for community transmission (6, 7). Previous mathematical modeling research indicated that mass antigen testing coupled with the quarantining of positive cases and their contacts could be an effective tool in mitigating pandemics compared to other infection control measures (8, 9). However, the performance of several commercial RDTs is highly variable, and additional methods are required to enhance their sensitivity for precise clinical diagnosis and to inform subsequent medical action (10).

The sensitivity of RDT can be improved by selecting appropriate specimens with alternative sampling techniques, which is critical to ensure accurate disease diagnosis without false test results. As an alternative to NPS, saliva-based specimens have attracted increasing attention for the diagnosis of respiratory infection (11–13). The noninvasive and self-collection methods associated with saliva specimens have the potential to increase population-based surveillance coverage without increasing the risk of exposure to nosocomial virus infections during the testing process. Several studies have reported that saliva, laden with virus, can serve as a transient medium for the transmission of SARS-CoV-2, which is broadly enriched on the epithelial cells lining the oral cavity and oral mucosae (14–16). Moreover, the U.S. Food and Drug Administration and the Centers for Disease Control and Prevention have updated their guidelines to include saliva-based COVID-19 testing (17, 18). Virus detection in saliva has been adopted by different techniques, such as chemiluminescence immunoassay (19), electrochemical analysis (20), and fluorescence assay (21). Although saliva specimens have advantages, including self-collection of a sample, it is difficult to obtain a sufficient volume of saliva specimens from patients with disease-caused dry mouth or xerostomia. Because saliva samples contain proteins, hormones, and therapeutic drugs and are too sticky to apply directly to the lateral flow assay, it is necessary to pretreat the saliva before assaying (22).

The current study aimed to improve the efficiency of COVID-19 diagnostics using a novel virus collection method with bean extract (BE)-based Beanguard gargle (BG, BIO3S, Inc., Republic of Korea). BG-RT-PCR and BG-Ag-RDT for SARS-CoV-2 exhibited outstanding analytical performance with the highest sensitivities being 100% (95% CI, 92.1 to 100) and 97.8% (95% CI, 88.4 to 99.6) compared to NPS-RT-PCR, respectively. Moreover, the BG-Ag-RDT exceeded the minimum performance criteria for early diagnosis of COVID-19 as recommended by the World Health Organization (WHO) (≥80% sensitivity and ≥ 97% specificity, compared to a standard PCR method) (5). These results suggested that the proposed BG-based virus collection method can serve as an efficient and promising strategy for self-diagnosis and on-site COVID-19 screening, thus facilitating the identification and subsequent isolation of symptomatic and asymptomatic active infections and the detection of SARS-CoV-2 variants.

## RESULTS

### Trial population characteristics.

The mean ages of the participants were 45.7 ± 16.0 years for patients with COVID-19 and 40.4 ± 11.18 years for healthy subjects (Table 1). Of the 102 patients initially confirmed by the government’s COVID-19 screening, 45 (44.1%) were sampled within 6 days of illness and 57 (55.9%) within 7 to 15 days of illness. Additionally, 27 (26.5%) patients were categorized as an asymptomatic group, while 75 (73.5%) were assigned to the COVID-19 symptomatic group characterized by symptoms, including cough (48.0%), sore throat (41.3%), fever (40.0%), headache (18.7%), myalgia (18.7%), chest pain (6.7%), chills (6.7%), fatigue (6.7%), diarrhea and nausea (5.3%), dry mouth (5.3%), and loss of taste or smell (4.0%). In addition, 8 (7.8%) and 2 (2.0%) of the 102 patients carried the SARS-CoV-2 B.1.1.7 (Alpha) and B.1.617.2 (Delta) variants, respectively.

**TABLE 1:** Characteristics of the participants

Characteristics	SARS-CoV-2 positive (*n* = 102)	Healthy subjects (*n* = 100)
Demographic composition of participants		
Sex (no. [%])		
Female	37 (36.3)	68 (68.0)
Male	65 (63.7)	32 (32.0)
Age (yrs)		
avg	45.7	40.4
Range	18–83	23–60
Patient with days of illness (no. [%])		
≤6 days	45 (44.1)	
>6 days	57 (55.9)	
Symptomatic (no. [%])	75 (73.5)	
Asymptomatic (no. [%])	27 (26.5)	
SARS-CoV-2 variant (no. [%])		
Alpha	8 (7.8)	
Delta	2 (2.0)	
Days of illness (days)		
Avg	7.9	
Range	2–15	

### Application of BG-RT-PCR for SARS-CoV-2 detection.

**FIG 1:**
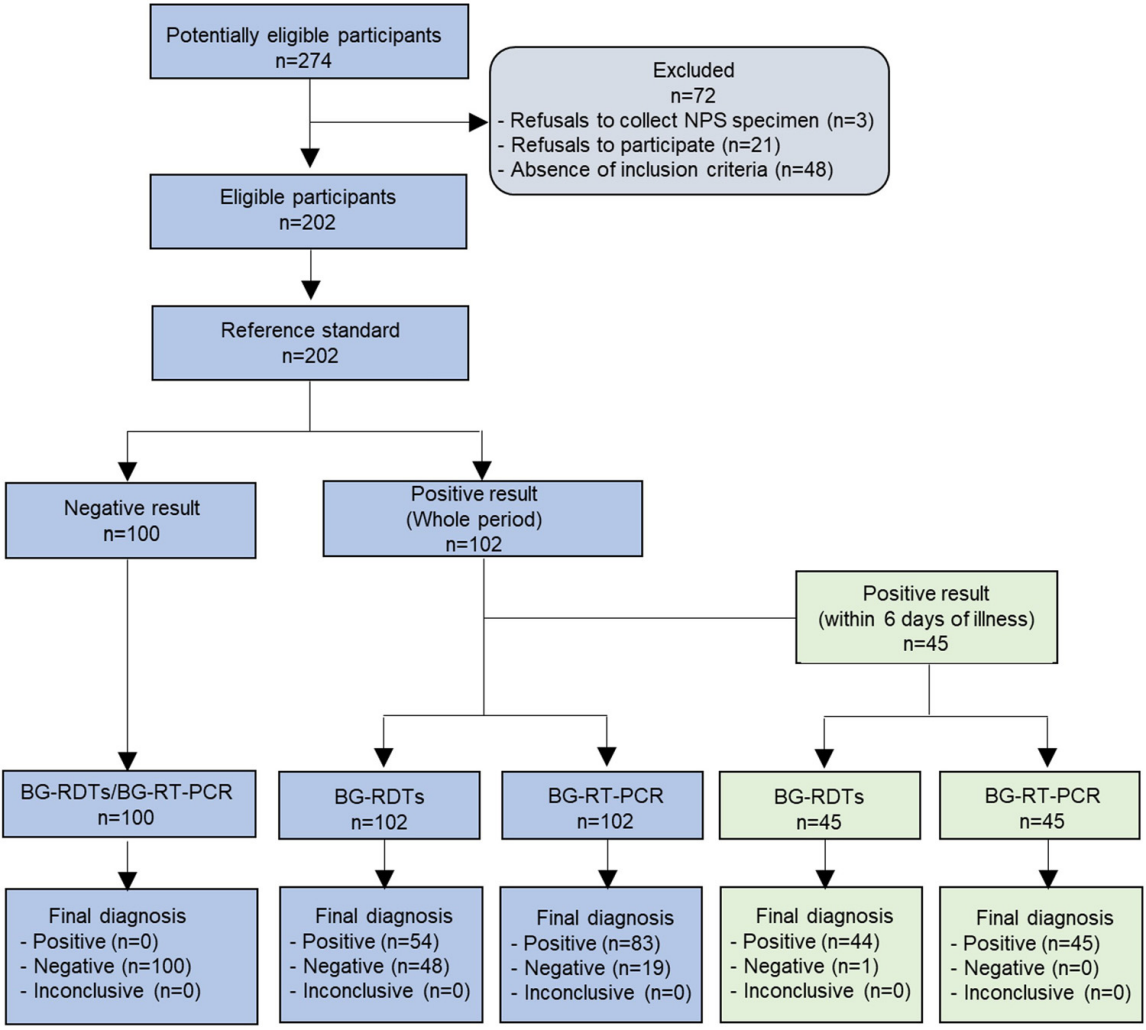
Enrollment and outcomes of participants in this clinical trial. Among total participants (*n* = 202), 86 participants (42.6%) were 18 to 39 years of age, 106 participants (52.5%) were aged 40 to 69 years, and 10 participants (5.0%) were older than 70 years. Nasopharyngeal swab-based RT-PCR (NPS-RT-PCR) was adopted as a reference standard.

Of the total 202 clinical specimens, 102 (50.5%) were positive via NPS-RT-PCR for SARS-CoV-2, with the cycle threshold (Ct) ranging from 11.1 to 39.3, and 100 (49.5%) were negative (Fig. 1 and Table S1). For 45 samples taken from individuals within 6 days of illness, both sensitivity and specificity of BG-RT-PCR against NPS-RT-PCR were 100% (95% confidence interval [CI], 92.1 to 100), and 100% (95% CI, 96.3 to 100), respectively (Table 2, Fig. S3A, and Table S2). In 11 asymptomatic cases within 6 days of initial confirmation of COVID-19, both sensitivity and specificity of BG-RT-PCR against NPS-RT-PCR were also 100% (95% CI, 74.1 to 100 and 95% CI, 20.7 to 100, respectively) (Fig. S1). All specimens tested positive for the SARS-CoV-2 Alpha (B.1.1.7) and Delta (B.1.617.2) variants were detectable using BG-RT-PCR within 6 days of illness (Table S3 and S4). In all COVID-19 positive patients in our clinical trial, the overall sensitivity of BG-RT-PCR was 81.4% (95% CI, 72.7 to 87.7) (Table S1).

**TABLE 2:** Evaluation of the diagnostic performance of BG-RT-PCR and BG-Ag-RDTs for negative controls (*n* = 100) and patients with COVID-19 within 6 days of illness (*n* = 45)

Test methods	Sensitivity (%)	Specificity (%)	PPA^*a*^ (%)	NPA^*a*^ (%)
	(95% CI)	(95% CI)	(95% CI)	(95% CI)
BG-RT-PCR compared with NPS-RT-PCR				
BG-RT-PCR (n)				
Participants (145)	100	100	100	100
	(92.1–100)	(96.3–100)	(92.1–100)	(96.3–100)
Asymptomatic (11)	100	100	100	100
	(74.1–100)	(20.7–100)	(74.1–100)	(20.7–100)
BG-Ag-RDT compared to BG-RT-PCR				
BG-Ag-RDT^*b*^ (n)				
Participants (145)	97.8	100	100	99.0
	(88.4–99.6)	(96.3–100)	(92.0–100)	(94.6-99.8)
Asymptomatic (11)	100	100	100	100
	(74.1–100)	(20.7–100)	(74.1–100)	(20.7–100)
BG-Ag-RDT compared to NPS-RT-PCR				
BG-Ag-RDT^*b*^ (n)				
Participants (145)	97.8	100	100	99.0
	(88.4–99.6)	(96.3–100)	(92.0–100)	(94.6–99.8)
Asymptomatic (11)	100	100	100	100
	(74.1–100)	(20.7–100)	(74.1–100)	(20.7–100)

a PPA, positive predictive agreement; NPA, negative predictive agreement.

b Two saliva-based Ag-RDTs were assessed: (i) STANDARD Q COVID-19 Ag Saliva test and (ii) Gmate COVID-19 Ag Saliva. There is no difference between the test results of two Ag-RDTs tested for negative controls and patients with COVID-19 within 6 days of illness.

**FIG 2:**
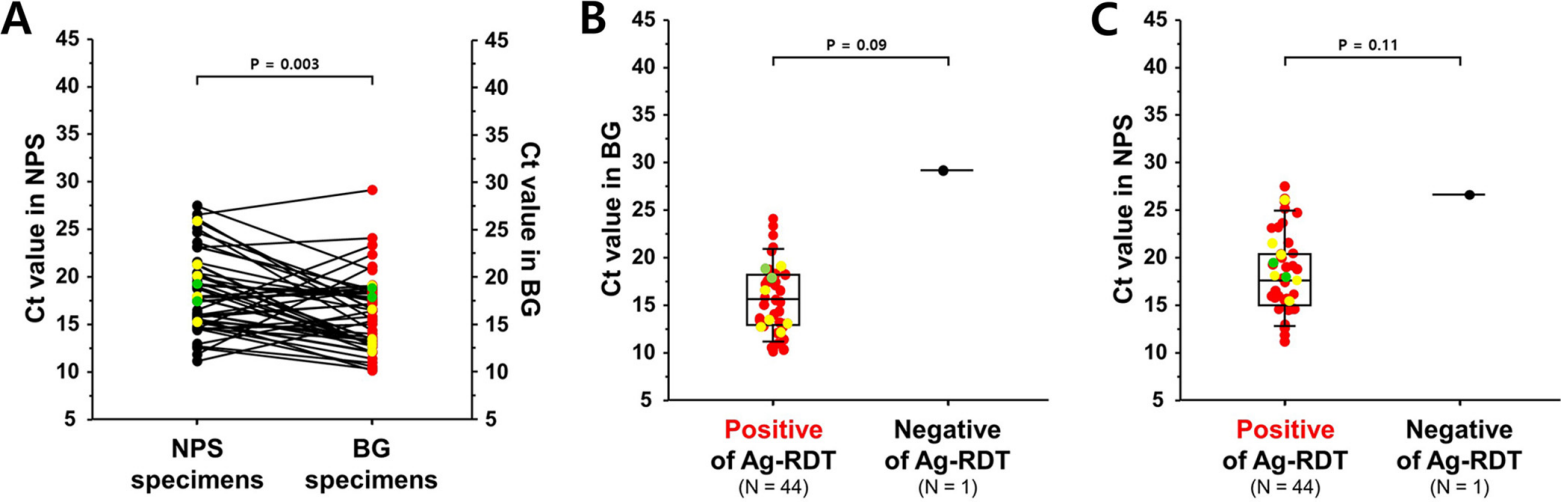
Comparison of Ct values from Beanguard gargle-based RT-PCR (BG-RT-PCR) with NPS-based RT-PCR (NPS-RT-PCR) analysis. (A) All patients within 6 days of initial COVID-19 confirmation. (B) Among the positive COVID-19 cases within 6 days of illness, the relationship between BG-based antigen rapid detection test results and Ct values of BG-RT-PCR with the RdRP gene for all patients is shown. (C) The relationship between BG-Ag-RDTs and the Ct values of NPS-RT-PCR within 6 days of patients with COVID-19 is shown. Yellow and green circles represent data points of Alpha and Delta variants, respectively.

Comparing the Ct values between BG-RT-PCR and NPS-RT-PCR samples from cases within 6 days of symptom onset or initial confirmation of COVID-19, the mean and median Ct values of BG-RT-PCR were 16.1 (95% CI,14.9 to 17.3) and 15.8 (interquartile range [IQR],13.0 to 18.3), respectively, whereas those of NPS-RT-PCR was 18.3 (95% CI,17.0 to 19.6) and 17.8 (IQR,15.2 to 20.4), respectively (Fig. 2A and Fig. S2). In asymptomatic cases within 6 days of initial confirmation, the mean and median Ct values of BG-RT-PCR were 15.9 (95% CI,13.4 to 18.4) and 15.8 (IQR,13.2 to 18.5), respectively, whereas those of NPS-RT-PCR was 18.7 (95% CI,15.8 to 21.6) and 17.9 (IQR,14.7 to 22.4), respectively (Fig. S1A and S2). Notably, the scatterplots also showed that the Ct values of BG-RT-PCR were positively correlated with those of NPS-RT-PCR, and their mean differences of Ct values for all cases and those within 6 days of illness were 2.1 and 2.2, respectively (Fig. S2).

### SARS-CoV-2 Ag-RDTs using BG-based saliva specimens.

The sensitivity and specificity of two commercial Ag-RDTs using BG-based saliva within 6 days of illness were 97.8% (95% CI, 88.4 to 99.6) and 100% (95% CI, 96.3 to 100), respectively (Table 2, Fig. S3B, and Table S2). Notably, the sensitivity and specificity of BG-Ag-RDT for asymptomatic cases within 6 days of initial confirmation were 100% (95% CI, 74.1 to 100) and 100% (95% CI, 20.7 to 100), respectively. BG-Ag-RDTs could detect all cases of SARS-CoV-2 Alpha and Delta variants within 6 days of illness (Table S3 and S4). Additionally, the sensitivity of BG-Ag-RDT for positive samples with Ct ≤30 within 6 days of illness was 97.8% (95% CI, 88.4 to 99.6; Table S5).

The BG-Ag-RDT results were also displayed with the Ct values of BG-RT-PCR and NPS-RT-PCR (Fig. 2B and C and Fig. S1B and C). In cases within 6 days of symptom onset or initial confirmation of COVID-19, the mean and median Ct values of BG-RT-PCR for positive Ag-RDT samples were 15.8 (95% CI, 14.7 to 16.9) and 15.7 (IQR, 12.9 to 18.3), respectively (Fig. 2B). For the asymptomatic cases within 6 days from the initial confirmation, the mean and median Ct values of BG-RT-PCR were 15.9 (95% CI, 13.4 to 18.4) and 15.8 (IQR, 13.2 to 18.5), respectively (Fig. S1B). Additionally, the mean and median Ct values of NPS-RT-PCR for the positive Ag-RDT samples within 6 days of illness were 18.1 (95% CI, 16.8 to 19.4) and 17.6 (IQR, 15.0 to 20.4), respectively (Fig. 2C). For the asymptomatic cases (initial confirmation within 6 days of illness), the mean and median Ct values of NPS-RT-PCR for positive BG-Ag-RDT samples were 18.7 (95% CI, 15.8 to 21.6) and 17.9 (IQR, 14.7 to 22.4), respectively (Fig. S1C).

### Interaction of active ingredient with SARS-CoV-2.

**FIG 3:**
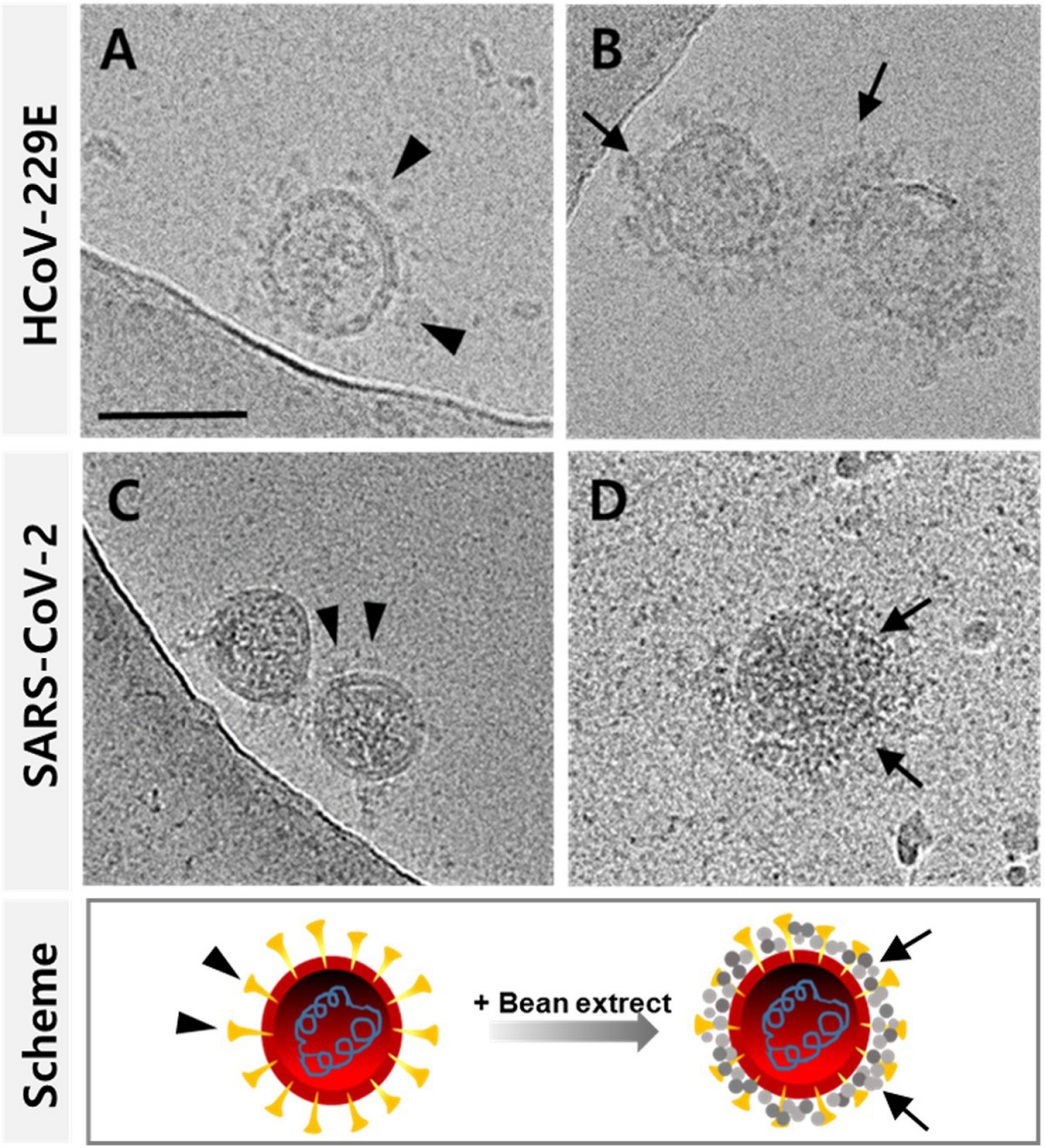
Interaction of bean extract (BE) with SARS-CoV-2. Cryo-EM images revealed the surface of HCoV-229E and SARS-CoV-2 surrounded by BE. Before (A) and after (B) exposure of BE to HCoV-229E and before (C) and after (D) exposure of BE to SARS-CoV-2 are presented with a schematic diagram, showing that BE is attached to the virus particle. The arrowheads and arrows indicate spike protein and BE, respectively. Scale bar, 100 nm.

To examine the interaction of HCoV-229E and SARS-CoV-2, belonging to the family Coronaviridae (23), with BE contained in the gargle, the ultrastructure of whole HCoV-229E and SARS-CoV-2 before and after treatment with BE was observed by cryo-electron microscopy (Cryo-EM) (Fig. 3A to D). The viral particles were spherical with club-shaped spikes embedded in the envelope. After BE treatment, BE covered the surface of the coronavirus particle, providing evidence that BE could effectively interact with SARS-CoV-2. Moreover, the results of transmission electron microscopy (TEM) using the negative staining method were consistent with those of cryo-EM (Fig. S4). To confirm that BE interferes with the interaction of hACE2 receptors and recombinant SARS-CoV-2 receptor binding domain (RBD)-Fc-tagged proteins using indirect enzyme-linked immunosorbent assay (ELISA), the RBD–hACE2 receptor complexes attached to ELISA wells were washed two times with BE at concentrations ranging from 0 to 140 ppm (Fig. S5A and B). ELISA results showed that BE effectively inhibited the binding of RBD and hACE2 in a dose-dependent manner up to 70 ppm.

We then identified the active ingredient of BE, truncated canavalin (TCan) (Fig. S6 and S7). The production of BE included the fermentation of sword beans containing canavalin and concanavalin A (ConA). After the removal of ConA through a 72 h fermentation process, the N-terminal region of the purified active ingredient (21.17 kDa) in the fermented bean extract was determined by Edman degradation as ^242^LSSQDKPFN^251^, which indicated that the N-terminal region of canavalin was truncated. TCan has a sequence coverage of 97% compared to the amino acid sequence of C. gladiata, P10562.1 by liquid chromatography-tandem mass spectrometry (LC-MS/MS). TCan has a stable three-dimensional structure and exists as an oligomer (Fig. S5C and S8). The purity of TCan was 99.91% (Fig. S6). Details regarding identification and molecular characterization of TCan are provided in Text S1. Furthermore, the binding capability of TCan to the two SARS-CoV-2 spike proteins, S1 and S2, was directly demonstrated using isothermal titration calorimetry (ITC) (Fig. S5D). TCan spontaneously bound to S1 and S2 (Fig. S5D) by releasing heat, i.e., in an exothermic reaction, which showed an energetically favorable negative value of enthalpy change (^ITC^Δ*H*_bind_). The dissociation constant (*K*_d_) of TCan for S1 and S2 ranged between ∼20 to ∼100 μM and ∼10 to ∼80 μM, respectively. Additional details regarding ITC are provided in Fig. S9 to S11. Based on ^ITC^ΔH_bind_, a good reporter of physicochemical properties of intermolecular interactions, possible interprotein forces at the interfaces of two complexes have been described (24–29). In general, attractive electrostatic and polar interactions (e.g., the salt bridge and hydrogen bonding) at a binding interface lead to the release of heat. Thus, these attractive interactions decrease the enthalpy of a protein complex and produce a negative ^ITC^ΔH_bind_ (^ITC^ΔH_bind_ <0). van der Waals attractions also contribute to a negative ^ITC^ΔH_bind_. As we revealed using the ITC analysis (Fig. S5D), the binding of TCan to S1 and S2 both resulted in a negative ^ITC^ΔH_bind_, suggesting that the attractive electrostatic and polar interactions and/or van der Waals attractions exist at a binding interface of the TCan-S protein. Because the magnitude of ^ITC^ΔH_bind_ depended on the type of S protein (i.e., TCan-S1 complex ≈ −6 to −7 kcal/mol; TCan-S2 complex ≈ −0.8 kcal/mol), it is conceivable that the physicochemical feature of each binding interface was distinct, and noncovalent forces decreasing ^ITC^ΔH_bind_ were more marked in the formation of the TCan-S1 complex.

### *In vivo* toxicity and *in vitro* cytotoxicity of BE.

To evaluate the toxicity of BE, the toxic effects of BE from sword beans (Canavalia gladiata) and concanavalin A on cell viability, lung and liver injuries, and inflammatory cells were compared. Concanavalin A, one of the components in sword bean, is well known for its mitogenic effect on splenocytes. It also activates the immune system, recruits lymphocytes, and elicits cytokine production (30). Results showed that BE neither induced toxic effects (based on analysis of survival rate, liver toxicity, and lung injury) nor exhibited cytotoxic effects or induce reactive oxygen species (ROS) generation (Fig. S12 to S19).

## DISCUSSION

Due to the COVID-19 pandemic and the rapid spread of multiple SARS-CoV-2 variants, the demand for diagnostic tests of SARS-CoV-2 infection is increasing. However, the conventional NPS-based RT-PCR method, adopted as a reference standard, requires skilled health workers and personal protective equipment for the workers during sampling. There is a risk of cross-infection due to the spread of contaminated aerosol during NPS specimen collection and handling. Moreover, NPS is not the first preference of patients due to the discomfort associated with insertion and removal of the swab. Hence, saliva as a specimen that can be self-collected could be a suitable alternative to NPS. Even though previous studies reported the application of saliva specimens for SARS-CoV-2 diagnosis, the sensitivity of saliva as a specimen was considerably lower than that of NPS (31, 32). Therefore, we focused on the use of a newly designed virus collection method, BG, for its user-friendliness and the convenience of virus collection from the oral cavity and increasing the efficacy of viral detection.

In this study, the BG-based virus collection method remarkably increased the sensitivity and specificity of Ag-RDT and RT-PCR for SARS-CoV-2 detection. Although the performance of saline gargle was evaluated for the detection of different respiratory viruses and SARS-CoV-2 using only RT-PCR, the application of saline gargle was not reported for Ag-RDTs (33–35). We showed that the BG-based virus collection method can be compatible with Ag-RDTs and allows for rapid diagnosis of COVID-19 during active infection without discriminating between symptomatic and asymptomatic cases.

The Alpha (B.1.1.7) and Delta (B.1.617.2) variants were spreading at a high rate in many countries, as well as in South Korea, during the study period (36). Compared with the NPS-RT-PCR results, we found the performance of the BG-RT-PCR within 6 days of illness to be 100% sensitive and specific for SARS-CoV-2, including the Alpha and Delta variants, suggesting a high sensitivity of BG-RT-PCR with comparable ability to NPS-RT-PCR. Furthermore, the sensitivity of the BG-Ag-RDT within 6 days of illness was enhanced up to 97.8% compared with the previously reported 65.0% sensitivity against salivary RT-PCR (37). All BG specimens of SARS-CoV-2, including Alpha and Delta variants, were also detected by BG-Ag-RDT, implying that the use of BG-Ag-RDT will facilitate rapid and accurate diagnosis of SARS-CoV-2 variants during active COVID-19 infection. Moreover, the diagnostic performance of the proposed BG-Ag-RDTs remarkably fulfilled WHO’s recommendations for the use of Ag-RDTs in early diagnosis of COVID-19 within 5 to 7 days of symptoms onset, which recommends ≥80% sensitivity and ≥97% specificity compared with that of the PCR assay (5).

One limitation of this study is the exclusion of three participants from NPS sampling due to the inability to confirm the test. Furthermore, due to the small sample size of Alpha and Delta variants available for this study, the generalization of our test results for the variants could be limited. Moreover, the performance of BG-Ag-RDT was not compared with salivary Ag-RDT in this study. This was due to the feasibility of simultaneously sampling the same patient in a clinical trial environment, which could be carried out with either gargle and NPS specimens or saliva and NPS specimens. The simultaneous request of the patient for gargle and saliva specimens within the limited clinical sample collection time frame in the COVID-19 quarantine hospital would affect the virus diagnosis results according to the order in which the gargle or simple saliva samples were collected. Therefore, in this study, we compared the performance of BG-Ag-RDT with WHO-recommended NPS-RT-PCR as this method is widely adopted and has high sensitivity.

The spike protein of SARS-CoV-2, S1 including receptor-binding domain (RBD) and S2, and its primary receptor hACE2 were extensively glycosylated. It is also known that canavalin possesses a substantial level of α-d-mannosidase activity (38). Investigation of the α-d-mannosidase activity of TCan has not yet been performed. Based on our combined studies on the interactions between TCan and SARS-CoV-2 viral proteins (i.e., RBD, S1, and S2), TCan is capable of efficiently capturing spike proteins. Microscopic (Fig. 3 and Fig. S4), biophysical (Fig. S8), and biochemical (Fig. S9) approaches convincingly revealed the binding of TCan to SARS-CoV-2 through protein-protein interactions. Thus, we reasoned that the exclusive and unique properties of TCan contributed to the improved sensitivity of the suggested diagnosis. In addition, the clinical trial results showed that collecting as much SARS-CoV-2 as possible from the oral cavity by gargling with a solution containing TCan can increase the accuracy of diagnosis.

In conclusion, based on the interaction between TCan and SARS-CoV-2, the BG-Ag-RDT offers an effective method with high sensitivity for self-diagnosis, on-site monitoring, and rapid testing of COVID-19. Moreover, this novel strategy will facilitate the execution of effective clinical responses by overcoming the long duration required for NPS-RT-PCR results and the low sensitivity of Ag-RDTs during active COVID-19 infection.

## MATERIALS AND METHODS

### Study design and participants.

In this study, paired NPS and BG-based saliva specimens were collected from 102 patients with COVID-19 who were hospitalized at Gunsan Medical Center, Namwon Medical Center, and Jeonbuk National University Hospital between May 7 and July 7 of 2021, and 100 healthy subjects as negative controls. Statistically, we estimated minimum sample size to obtain 95% sensitivity. A schematic study design and procedures are shown in Text S1. The specimens were then analyzed with NPS-RT-PCR, BG-RT-PCR, and BG-Ag-RDTs. The baseline clinical and demographic data of enrolled patients, presented in Table 1 and Fig. 1, did not show any significant differences. The date of a patient’s clinical symptoms onset, hospitalization, and initial confirmation of COVID-19 was recorded by health care professionals (Supplemental material 1). Ethical approval for the study was granted by the Institutional Review Board of Jeonbuk National University Hospital (CUH2021-04-036-002) and written informed consent was obtained from all participants. The inclusion and exclusion criteria are listed in Supplemental material 1.

### Procedures.

Medical professionals collected NPS specimens as a reference from participants and saliva specimens by asking participants to spit into a tube after swirling and gargling 5 mL of BG for 2 min. The RT-PCR diagnosis for SARS-CoV-2 in NPS and saliva specimens was performed using Allplex 2019-nCoV Real-time PCR (Seegene, Seoul, Republic of Korea), with RNA-dependent RNA polymerase (RdRP), envelope protein (E), and nucleocapsid protein (N) genes as the targets, and a STANDARD M nCoV Real-Time Detection kit (SD BIOSENSOR, Suwon, Republic of Korea), with RdRP and E genes as the targets, according to the manufacturer’s instructions. When all target genes were detected, the RT-PCR result for SARS-CoV-2 RNA was considered positive. For BG-Ag-RDTs, we used two different COVID-19 Ag-RDT kits, namely, STANDARD Q COVID-19 Ag Saliva test (SD BIOSENSOR, Suwon, South Korea) and Gmate COVID-19 Ag Saliva (AG-020, Philosys Co., Ltd., Republic of Korea). BG-based saliva specimens (200 μL) were gently mixed with equal volumes of extraction buffer included in each COVID-19 antigen test kit. Subsequently, the treated specimen was added to the sample well of the test cassette, according to the manufacturer’s instructions. The test results obtained from Ag-RDTs were confirmed with the naked eye and the appearance of a colored band in both the control and test lines indicates a positive result.

The interaction of BE with the receptor-binding domain (RBD)-human version of angiotensin-converting enzyme 2 (hACE2) complex was evaluated by ELISA, and the encapsulation of BE on HCoV-229E and SARS-CoV-2 surface was visualized by cryo-EM. Intermolecular interactions between active ingredient, truncated canavalin (TCan) purified from BE, and spike proteins and molecular properties of TCan were examined by calorimetry and various biophysical approaches, respectively. Further details on calorimetry, molecular characterization, cryo-EM, ELISA, cell viability, reactive oxygen species (ROS) production, and statistical analysis are described in Supplemental material 1.

### Statistical analysis.

All statistical analyses were performed using GraphPad Prism v.7, and statistical multiple comparisons were performed using one-way ANOVA followed by *post hoc* Dunnett’s test. Data were expressed as mean ± SD. Significance was set at *P <* 0.05. We determined the difference between BG-RT-PCR and NPS-RT-PCR for SARS-CoV-2 using two-sided Wilcoxon signed-rank and two-sided Mann-Whitney rank-sum tests. We reported the diagnostic accuracy of sensitivity, specificity, positive percent agreement, and negative percent agreement with 95% confidence intervals. Bland-Altman analysis was used to assess method agreement between BG-RT-PCR and NPS-RT-PCR. The average refers to the mean Ct values of BG-RT-PCR and NPS-RT-PCR, and the difference was obtained by subtracting the Ct values for BG-RT-PCR from those of NPS-RT-PCR.

The minimal sample size was calculated with 90% power at a significance level of 0.05 for the estimated sensitivity and specificity, according to the equation:

$$\mathrm{Sample size (n)}=\frac{{\left(Z_{(1-\alpha )/2}+Z_{1-\beta }\right)}^{2}\times p(1-p)}{{\left(p-p_{0}\right)}^{2}}$$

Here, *Z*_(1-α)/2_ and *Z*_1-β_ were Z-values for the corresponding level of confidence and the desired power, respectively. *p* was the estimated sensitivity or specificity, and *p*_0_ was the sensitivity or specificity of the reference. In this study, *n* = 50 for 95% sensitivity with 85% reference sensitivity, and *n* = 65 for 99% specificity with 95% reference specificity.

### Data availability.

The complete deidentified participant data set is available upon request to dskim@jbnu.ac.kr for researchers whose proposed use of the data has been approved for any purpose. Data will be available with publication. If needed, requests will require the ethics committee approval of the Jeonbuk National University Hospital in Jeonju (Republic of Korea). Anonymized data are fully available upon reasonable request from the corresponding author after approval by the hospital ethics committee.
