# Supplementary material for: Bean Extract-Based Gargle for Efficient Diagnosis of Active COVID-19 Infection Using Rapid Antigen Tests
Source: Microbiol Spectr. 2022 Feb 16;10(1):e01614-21. doi: 10.1128/spectrum.01614-21 (PMC8849053; doi:10.1128/spectrum.01614-21)
Supplement: SUPPLEMENTAL FILE 1 — Supplemental material. Download SPECTRUM01614-21_Supp_1_seq5.pdf, PDF file, 4.9 MB [file spectrum01614-21_supp_1_seq5.pdf]

Supplementary Appendix 1. Raw Data from 102 Patients with COVID-19

Column	Description
1	Serial Number
2	Clinical Trial Patient Number
3	Age (years)
4	Sex (Man=1, Woman=2)
5	Clinical Trial Date (mm-dd-yy)
6	Initial Confirmed Date by NPS-PCR (mm-dd-yy)
7	Hospitalization Date (mm-dd-yy)
8	Hospitaliztion Period (day) (from hospitalization date to clinical trial date)
9	Hospitaliztion Period of Symptomatic case (day) (from symtoms onset date to clinical trial date)
10	Hospitaliztion Period of Asymptomatic case (day) (from initial confirmed date to clinical trial date)
11	Presence or absence of symptoms at diagnosis (presence=1, absence=2)
12	Date of Symptoms onset (mm-dd-yy)
13	Clinical Symtoms at diagnosis
14	Routes of transmission/infection (close contact=1, unknown=2)
15	Presence of SARS-CoV-2 Variant (Alpha=1, Delta=2)
16	NPS_PCR_Ct value, E gene
17	NPS_PCR_Ct value, RdRP gene
18	NPS_PCR_Ct value, N gene
19	NPS_PCR_Result (Positive=1, Negative=2)
20	Saliva_SARS-CoV-2 Antigen RDT [STANDARD Q COVID-19 Ag Saliva test (SD BIOSENSOR)] (Positive=1, Negative=2)
21	Saliva_SARS-CoV-2 Antigen RDT [Gmate® COVID-19 Ag Saliva (Philosys)] (Positive=1, Negative=2)
22	Saliva_PCR_Result (Positive=1, Negative=2, Inconclusive=3)
23	Saliva_PCR_Ct value, RdRP gene
24	Saliva_PCR_Ct value, E gene

Supplementary Appendix 1. Raw Data from 102 Patients with COVID-19

1	2	3	4	5	6	7	8	9	10	11	12	13	14	15	16	17	18	19	20	21	22	23	24
**1**	J001	45	2	05-07-21	04-23-21	04-23-21	15	15	15	1	04-23-21	fever, sore throat	2		30.92	31.79	33.78	1	2	2	3	34.35	negative
**2**	J003	35	1	05-07-21	05-01-21	05-03-21	5	8	8	1	04-30-21	cough	1		31.02	31.49	30.70	1	2	2	3	34.32	negative
**3**	J005	38	1	05-07-21	05-03-21	05-03-21	5	7	7	2			1		28.73	29.17	28.69	1	2	2	1	26.77	26.06
**4**	J006	79	1	05-07-21	04-26-21	04-27-21	11	>11	11	1		cough	1		35.35	35.01	33.94	1	2	2	1	33.23	31.72
**5**	J008	70	1	05-07-21	04-26-21	04-27-21	11	13	13	1	04-25-21	fatigue, muscle ache	1		21.84	21.54	21.75	1	2	2	1	22.04	22.35
**6**	J009	47	1	05-07-21	05-01-21	05-01-21	7		7	2			1	1	27.54	28.88	26.74	1	1	1	1	19.82	19.53
**7**	J011	38	1	05-07-21	05-02-21	05-02-21	6	6	6	1	05-01-21	fever, sputum, sore throat	2	1	16.12	17.78	15.38	1	1	1	1	13.05	13.29
**8**	J012	46	1	05-07-21	05-01-21	05-02-21	6	6	6	1	05-01-21	cough	2	1	19.11	20.11	17.35	1	1	1	1	13.43	13.05
**9**	J015	65	1	05-07-21	04-29-21	04-29-21	9	11	11	1	04-27-21	fever, sore throat, cold sweat, chest tightness	2		30.24	31.00	32.40	1	2	2	3	negative	34.00
**10**	J016	63	2	05-07-21	04-29-21	04-29-21	9	11	11	1	04-27-21	fever	1		19.65	19.74	21.38	1	1	2	1	16.01	16.31
**11**	J018	56	2	05-07-21	05-02-21	05-02-21	6	14	14	2			1		31.05	31.43	30.76	1	2	2	1	31.44	30.92
**12**	J019	75	2	05-07-21	04-25-21	04-26-21	12		13	2		cough, headache	2		33.14	32.72	31.34	1	2	2	1	18.35	18.54
**13**	J020	34	1	05-07-21	05-02-21	05-03-21	5	5	5	2			1	1	17.69	17.91	15.65	1	1	1	1	19.08	18.97
**14**	J021	50	1	05-07-21	05-02-21	05-03-21	3	6	6	1	05-02-21	muscle ache, chills, headache	1	1	20.57	21.30	18.53	1	1	1	1	12.69	12.72
**15**	J022	63	2	05-07-21	05-03-21	05-04-21	4	4	4	1	05-04-21	fever, cough	1		20.64	20.43	19.62	1	1	1	1	17.59	17.84
**16**	J023	35	1	05-07-21	05-04-21	05-04-21	3	3	3	1	05-04-21	fever, sore throat, muscle ache	2		10.50	12.52	9.97	1	1	1	1	10.32	10.44
**17**	J024	55	1	05-07-21	05-04-21	05-05-21	3	5	5	1	05-03-21	fever, sore throat, muscle ache, cough	2		11.93	14.58	10.90	1	1	1	1	10.56	10.74
**18**	J025	53	1	05-07-21	05-04-21	05-05-21	3		4	2			1		24.43	21.57	21.21	1	1	1	1	17.46	17.64
**19**	J026	20	2	05-07-21	05-05-21	05-06-21	2		3	2			1		16.76	18.78	16.33	1	1	1	1	12.07	12.07
**20**	J027	55	1	05-07-21	05-03-21	05-04-21	4	4	4	1	05-04-21	fever, chest tightness, headache, foreign body sensation of throat	1	1	11.80	15.22	11.04	1	1	1	1	12.12	12.29
**21**	J028	60	1	05-07-21	04-30-21	04-30-21	8	>8	8	1		dry mouth, fatigue	1		32.56	31.92	33.08	1	2	2	1	21.31	20.89
**22**	J029	59	1	05-07-21	04-27-21	04-28-21	10	12	12	1	04-26-21	sore throat, fever, sputum, throat tightness	2		31.13	31.07	33.96	1	2	2	1	28.04	27.46
**23**	J031	83	1	05-07-21	04-29-21	04-29-21	9		9	2			1		37.87	36.69	37.67	1	2	2	1	29.19	28.24

Supplementary Appendix 1. Raw Data from 102 Patients with COVID-19

1	2	3	4	5	6	7	8	9	10	11	12	13	14	15	16	17	18	19	20	21	22	23	24
**24**	J032	34	1	05-07-21	05-01-21	05-02-21	6	6	6	1		fever, flu sensation	1	1	24.41	25.89	22.21	1	1	1	1	16.57	16.47
**25**	J033	34	1	05-07-21	04-27-21	04-27-21	11	12	12	1	04-26-21	fever, cough	1	1	21.50	22.89	21.07	1	2	2	1	22.31	21.84
**26**	J036	71	1	05-14-21	05-04-21	05-05-21	10	11	11	1		sore throat	2		34.38	34.95	33.19	1	2	2	3	35.96	negative
**27**	J037	64	2	05-14-21	05-04-21	05-05-21	10	11	11	1	05-05-21	dry mouth, fatigue	1		30.56	32.47	29.71	1	2	2	2	negative	negative
**28**	J038	62	1	05-14-21	05-04-21	05-04-21	11		11	2			1		38.65	39.3	38.73	1	2	2	2	negative	negative
**29**	J040	60	1	05-14-21	05-11-21	05-12-21	3		4	2			1		12.94	14.55	18.58	1	1	1	1	15.03	14.98
**30**	J041	21	1	05-14-21	05-11-21	05-12-21	3		4	2			1		16.26	15.7	19.59	1	1	1	1	10.13	10.15
**31**	J042	51	1	05-14-21	05-13-21	05-13-21	2		3	2			2		23.61	23.18	24.12	1	1	1	1	18.71	18.44
**32**	J043	38	1	05-14-21	05-13-21	05-13-21	2	2	2	1	05-13-21	fever, sore throat, muscle ache	2		15.78	15.95	17.27	1	1	1	1	17.14	15.61
**33**	J044	58	1	05-14-21	05-13-21	05-13-21	2	4	4	1	05-11-21	cough, rhinorrhea	2		24.15	23.66	24.22	1	1	1	1	17.43	17.22
**34**	J045	27	1	05-14-21	05-13-21	05-13-21	2	3	3	1	05-12-21	sore throat	1		15.94	15.52	19.08	1	1	1	1	13.63	13.2
**35**	J047	51	2	05-14-21	05-08-21	05-08-21	7		7	2			1		33.61	34.81	32.29	1	2	2	2	negative	negative
**36**	J048	36	1	05-14-21	05-13-21	05-13-21	2	3	3	1	05-12-21	sputum	1		13.25	14.54	16.6	1	1	1	1	13.41	12.73
**37**	J049	32	1	05-14-21	05-12-21	05-12-21	3	>10	10	1		decreased taste sense, anosmia	1		35.33	35.46	36.64	1	2	2	1	31.6	30.66
**38**	J050	76	2	05-14-21	05-11-21	05-11-21	4	>10	10	2			2		33.49	32.53	35.67	1	2	2	1	31.52	30.56
**39**	J051	53	2	05-14-21	05-10-21	05-10-21	5	12	12	1	05-03-21	chills, sputum	1		33.75	33.47	36.12	1	2	2	1	29.12	28.24
**40**	J052	72	2	05-14-21	05-10-21	05-10-21	5	15	15	1	04-30-21	cough	2		35.81	35.4	35.39	1	2	2	2	negative	negative
**41**	J054	29	1	05-14-21	05-12-21	05-13-21	2	5	5	1	05-10-21	cough, fever, cold sweating	1		16.09	16.18	18.78	1	1	1	1	12.74	12.04
**42**	J055	54	2	05-14-21	05-12-21	05-12-21	3	3	3	2			1		15.22	14.78	17.7	1	1	1	1	11.39	11.33
**43**	J056	37	1	05-14-21	05-12-21	05-12-21	3	3	3	1	05-12-21	cough, sputum, chills	2		11.88	12.68	15.84	1	1	1	1	10.95	10.8
**44**	J057	21	1	05-14-21	05-11-21	05-12-21	3	3	3	2			1		25.36	25.16	28.19	1	1	1	1	14.37	14.37
**45**	J058	44	2	05-14-21		05-13-21	2	4	4	1	05-11-21	cough, sputum	1		16.2	15.96	18.85	1	1	1	1	18.65	18.15
**46**	J060	52	2	05-14-21	05-01-21	05-01-21	14	>14	14	2			2		34.95	36.13	34.72	1	2	2	1	35.34	34.86
**47**	J061	32	1	05-14-21	05-13-21	05-13-21	2	3	3	1	05-12-21	cough, muscle ache, sputum, rhinorrhea	1		15.68	16.13	19.2	1	1	1	1	13.2	13.6
**48**	J062	45	1	05-14-21	05-13-21	05-13-21	2		2	2			1		12.65	14.43	16.69	1	1	1	1	15.81	15.05
**49**	J064	83	1	05-14-21	05-10-21	05-10-21	5	12	12	2			2		27.55	28.21	29.25	1	1	1	1	24.16	24.44
**50**	J065	54	2	05-14-21	05-06-21	05-06-21	9	11	11	1	05-04-21	fever, cough	2		33.08	33.73	31.98	1	2	2	3	negative	35.54

Supplementary Appendix 1. Raw Data from 102 Patients with COVID-19

1	2	3	4	5	6	7	8	9	10	11	12	13	14	15	16	17	18	19	20	21	22	23	24
**51**	J068	39	2	05-14-21	05-13-21	05-13-21	2		2	2			2		13.22	14.57	18.17	1	1	1	1	22.37	21.62
**52**	J069	31	1	05-14-21	05-13-21	05-13-21	2	2	2	1	05-13-21	fatigue, dizness	1		10.28	11.14	14.63	1	1	1	1	15.36	14.65
**53**	J070	76	2	05-14-21	05-10-21	05-10-21	5	8	8	1	05-07-21	fever and chills	2		36.74	35.49	37.58	1	2	2	3	negative	35.02
**54**	J071	30	1	05-14-21	05-09-21	05-09-21	6	6	6	1	05-09-21	cough, fever	2		25.33	26.20	25.07	1	1	1	1	15.55	15.55
**55**	J072	42	1	05-14-21	05-13-21	05-13-21	2	2	2	1	05-13-21	sore throat, rhinorrhea	2		15.48	15.30	18.6	1	1	1	1	14.03	13.2
**56**	J073	39	1	05-14-21	05-13-21	05-13-21	2	2	2	1	05-13-21	headache	1		19.97	19.48	22.45	1	1	1	1	12.83	12.35
**57**	J074	36	1	05-14-21	05-13-21	05-13-21	2	2	2	1	05-13-21	cough	1		16.2	16.51	19.09	1	1	1	1	23.34	21.82
**58**	J075	37	1	05-14-21	05-13-21	05-13-21	2	2	2	1	05-13-21	cough, muscle ache	1		15.75	15.74	18.53	1	1	1	1	17.21	15.74
**59**	J081	52	1	05-14-21	05-11-21	05-11-21	4	8	8	1	05-07-21	cough	2		21.03	20.54	24.21	1	1	1	1	23.38	23
**60**	J086	31	2	05-27-21	05-21-21	05-21-21	7	8	8	1	05-20-21	rhinorrhea, headache	1		35.56	38.91	33.52	1	2	2	3	negative	34.49
**61**	J093	20	1	05-27-21	05-22-21	05-22-21	6	6	6	1	05-22-21	headache, sore throat, cough	1		18.16	19.12	18.61	1	1	1	1	18.36	16.99
**62**	J094	72	1	05-27-21	05-21-21	05-22-21	6	6	6	2			1		24.00	24.72	23.71	1	1	1	1	18.21	17.00
**63**	J096	44	2	05-27-21	05-22-21	05-22-21	6	6	6	1	05-22-21	chills, cough	1		16.64	17.81	16.74	1	1	1	1	18.28	17.07
**64**	J097	48	2	05-27-21	05-22-21	05-22-21	6	9	9	1	05-19-21	headache, chills, fever	1		20.17	21.01	20.41	1	1	1	1	19.80	18.63
**65**	J101	43	2	05-27-21	05-25-21	05-25-21	3	8	8	1	05-20-21	cough, sputum, rhinorrhea	1		18.74	18.81	22.24	1	1	1	1	27.38	25.54
**66**	J105	24	2	05-27-21	05-20-21	05-21-21	7	10	10	1	05-18-21	sore throat, cough, nasal stuffness	1		31.35	33.08	31.21	1	2	2	2	negative	negative
**67**	J107	59	1	05-27-21	05-18-21	05-19-21	9	>10	10	2			1		28.79	30.09	28.73	1	2	2	1	31.51	29.40
**68**	J116	26	2	06-02-21	05-30-21	05-31-21	3	4	4	1	05-30-21	chills, cough, headache	1		10.64	11.84	16.87	1	1	1	1	21.11	20.68
**69**	J117	23	2	06-02-21	05-30-21	05-31-21	3	6	6	1	05-28-21	sore throat, abdominal pain, diarrhea	1		20.61	20.37	24.58	1	1	1	1	12.74	12.79
**70**	J121	36	1	06-02-21	05-26-21	05-27-21	7	9	9	1	05-25-21	cough, muscle ache	1		18.48	19.54	18.65	1	1	1	1	24.50	24.05
**71**	J122	43	2	06-02-21	05-29-21	05-29-21	5	5	5	1		dizness, sputum, fever, nasal stuffness	2		26.74	27.51	30.08	1	1	1	1	20.72	19.70
**72**	J123	29	1	06-08-21	06-02-21	06-03-21	6		7	2		4/12, AstraZeneca first shot	1		34.47	34.01	36.96	1	2	2	3	negative	34.67
**73**	J126	57	1	06-08-21	06-04-21	06-05-21	4	6	6	1	06-03-21	chills, cough, sore throat	1		20.06	19.95	22.49	1	1	1	1	16.53	15.71
**74**	J127	52	1	06-08-21	06-04-21	06-04-21	5	6	6	1	06-03-21	cough	1		19.29	18.98	22.25	1	1	1	1	12.99	12.39

Supplementary Appendix 1. Raw Data from 102 Patients with COVID-19

1	2	3	4	5	6	7	8	9	10	11	12	13	14	15	16	17	18	19	20	21	22	23	24
**75**	J129	23	2	06-08-21	05-30-21	05-31-21	9	12	12	1	05-28-21	sore throat, abdominal pain, diarrhea	1		29.72	29.58	32.44	1	2	2	1	26.65	25.62
**76**	J130	57	2	06-08-21	06-01-21	06-02-21	7	10	10	1	05-29-21	chills, muscle ache	1		34.88	35.04	32.97	1	2	2	2	negative	negative
**77**	J131	33	2	06-08-21	06-02-21	06-03-21	6	7	7	1	06-02-21	headache, muscle ache	1		27.17	27.18	26.31	1	1	1	1	16.13	15.62
**78**	J144	48	1	06-15-21	06-07-21	06-07-21	9	10	10	1	06-06-21	cough, muscle ache, fever	1		29.95	30.70	31.90	1	2	2	1	32.10	30.09
**79**	J145	57	1	06-15-21	06-08-21	06-09-21	7	13	13	1	06-03-21	sputum, sore throat	1		24.73	34.37	28.64	1	1	1	1	24.51	22.89
**80**	J146	69	1	06-15-21	06-04-21	06-05-21	11	12	12	1	06-04-21	muscle ache	1		24.71	24.56	27.66	1	2	2	1	29.52	27.01
**81**	J147	46	1	06-15-21	06-04-21	06-05-21	11	12	12	1	06-04-21	fever, entry from Cambodia	2		35.82	38.11	35.94	1	2	2	1	34.44	32.41
**82**	J148	42	1	06-15-21	06-03-21	06-03-21	13	14	14	1	06-02-21	fever, sputum, 5/27, AstraZeneca first shot	1		28.85	29.17	32.65	1	1	1	1	17.40	16.06
**83**	J149	27	2	06-15-21	06-03-21	06-04-21	12	12	12	1	06-03-21	cough, sputum, nasal stufness, diarrhea, muscle ache, fever	1		31.66	31.25	33.91	1	2	2	2	negative	negative
**84**	J150	52	2	06-15-21	06-09-21	06-10-21	6	6	6	1	06-10-21	cough, sputum, fever	1		22.71	23.11	24.70	1	1	1	1	24.10	21.82
**85**	J151	48	2	06-15-21	06-07-21	06-11-21	5	9	9	2			1		30.90	31.30	33.10	1	2	2	1	27.34	25.61
**86**	J152	48	2	06-15-21	06-04-21	06-05-21	11	13	13	1	06-03-21	headache, cough	1		31.25	31.79	34.32	1	2	2	1	27.11	25.20
**87**	J153	22	1	06-15-21	06-02-21	06-03-21	13	15	15	1	06-01-21	fever	1		31.78	32.64	31.67	1	2	2	2	negative	negative
**88**	J154	58	1	06-15-21	06-04-21	06-04-21	12	15	15	1	06-01-21	common cold sense	2		34.65	37.05	36.33	1	2	2	2	negative	negative
**89**	J155	46	2	06-15-21	06-02-21	06-02-21	14	14	14	1	06-02-21	fever	1		27.67	28.67	27.53	1	2	2	1	30.20	28.40
**90**	J157	40	1	06-15-21	06-08-21	06-09-21	7	12	12	1	06-04-21	chills, common cold sense	1		28.92	29.12	31.42	1	2	2	1	24.79	23.17
**91**	J158	61	1	07-07-21	07-04-21	07-05-21	3	13	13	2			1		34.66	34.20	32.29	1	2	2	3	negative	33.28
**92**	J159	22	1	07-07-21	07-04-21	07-05-21	3	10	10	1	06-28-21	fever, cough, diarrhea, body weght loss (5kg)	1		33.68	34.87	32.95	1	2	2	3	negative	33.23
**93**	J160	64	2	07-07-21	07-06-21	07-06-21	2	9	9	1	06-29-21	chills, muscle ache, anosmia, headache	2		27.18	27.79	30.50	1	2	2	1	27.30	26.19
**94**	J161	46	1	07-07-21	07-06-21	07-06-21	2	11	11	1	06-27-21	nausea, anosmia	1		30.82	31.94	30.61	1	2	2	1	33.84	33.28
**95**	J162	39	1	07-07-21	07-03-21	07-04-21	4	6	6	1	07-02-21	muscle ache, fever, cold sweating, entry from Russia	2	2	18.84	19.25	19.37	1	1	1	1	17.85	17.52
**96**	J165	37	1	07-07-21	07-02-21	07-02-21	6	12	12	1	06-30-21	fever, cough, headache	1		21.79	22.62	21.87	1	2	2	1	23.85	23.36

Supplementary Appendix 1. Raw Data from 102 Patients with COVID-19

1	2	3	4	5	6	7	8	9	10	11	12	13	14	15	16	17	18	19	20	21	22	23	24
**97**	J166	46	1	07-07-21	07-01-21	07-02-21	6	12	12	2			1		22.95	24.28	22.47	1	2	2	1	30.24	29.53
**98**	J167	25	2	07-07-21	07-05-21	07-05-21	3	6	6	1	07-02-21	chills, cough	2		25.39	26.59	24.99	1	2	2	1	29.18	28.42
**99**	J168	18	1	07-07-21	07-01-21	07-02-21	6	9	9	2			1		19.15	18.92	22.07	1	2	2	1	26.01	25.14
**100**	J171	18	1	07-07-21	06-30-21	07-01-21	7	8	8	1	06-30-21	fever, rhinorrhea	1		22.62	23.98	23.06	1	2	2	1	27.46	26.73
**101**	J173	45	2	07-07-21	07-04-21	07-04-21	4	5	5	1	07-03-21	headache, sore throat, fever sensation	1	2	16.93	17.41	17.21	1	1	1	1	18.82	18.64
**102**	J174	24	2	07-07-21	07-05-21	07-05-21	3	3	3	1	07-05-21	headache, eye pain, fever, cough	1	2	12.59	12.94	17.74	1	1	1	1	16.42	16.35

Supplementary Appendix 1. Raw Data from 100 Healthy Individuals as Negative Control

No.	Clinical Trial Healthy Control Number	Sex (Man=1, Woman=2)	Age (years)	NPS_PCR _Ct value E gene	NPS_PCR _Ct value RdRP gene	NPS_PCR _Ct value N gene	NPS_PCR_Result (Positive=1, Negative=2)	Antigen RDT [STANDARD Q COVID-19 Ag Saliva test (SD BIOSENSOR)] (Positive=1, Negative=2)	Antigen RDT [Gmate® COVID-19 Ag Saliva (Philosys)] (Positive=1, Negative=2)	Saliva_PCR _Result (Positive=1, Negative=2, Inconclusive=3)	Saliva_PCR _Ct value RdRP gene	Saliva_PCR _Ct value E gene
1	N001	2	57	negative	negative	negative	2	2	2	2	negative	negative
2	N002	1	39	negative	negative	negative	2	2	2	2	negative	negative
3	N003	2	46	negative	negative	negative	2	2	2	2	negative	negative
4	N004	2	25	negative	negative	negative	2	2	2	2	negative	negative
5	N005	2	47	negative	negative	negative	2	2	2	2	negative	negative
6	N006	1	56	negative	negative	negative	2	2	2	2	negative	negative
7	N007	2	56	negative	negative	negative	2	2	2	2	negative	negative
8	N008	2	41	negative	negative	negative	2	2	2	2	negative	negative
9	N009	2	25	negative	negative	negative	2	2	2	2	negative	negative
10	N010	2	52	negative	negative	negative	2	2	2	2	negative	negative
11	N011	2	53	negative	negative	negative	2	2	2	2	negative	negative
12	N012	2	24	negative	negative	negative	2	2	2	2	negative	negative
13	N013	2	50	negative	negative	negative	2	2	2	2	negative	negative
14	N014	1	44	negative	negative	negative	2	2	2	2	negative	negative
15	N015	2	31	negative	negative	negative	2	2	2	2	negative	negative
16	N016	2	30	negative	negative	negative	2	2	2	2	negative	negative
17	N017	1	51	negative	negative	negative	2	2	2	2	negative	negative
18	N018	2	51	negative	negative	negative	2	2	2	2	negative	negative
19	N019	2	48	negative	negative	negative	2	2	2	2	negative	negative
20	N020	2	50	negative	negative	negative	2	2	2	2	negative	negative
21	N021	2	25	negative	negative	negative	2	2	2	2	negative	negative
22	N022	2	26	negative	negative	negative	2	2	2	2	negative	negative
23	N023	2	43	negative	negative	negative	2	2	2	2	negative	negative

Supplementary Appendix 1. Raw Data from 100 Healthy Individuals as Negative Control

No.	Clinical Trial Healthy Control Number	Sex (Man=1, Woman=2)	Age (years)	NPS_PCR _Ct value E gene	NPS_PCR _Ct value RdRP gene	NPS_PCR _Ct value N gene	NPS_PCR_Result (Positive=1, Negative=2)	Antigen RDT [STANDARD Q COVID-19 Ag Saliva test (SD BIOSENSOR)] (Positive=1, Negative=2)	Antigen RDT [Gmate® COVID-19 Ag Saliva (Philosys)] (Positive=1, Negative=2)	Saliva_PCR _Result (Positive=1, Negative=2, Inconclusive=3)	Saliva_PCR _Ct value RdRP gene	Saliva_PCR _Ct value E gene
24	N024	2	48	negative	negative	negative	2	2	2	2	negative	negative
25	N025	1	48	negative	negative	negative	2	2	2	2	negative	negative
26	N026	2	43	negative	negative	negative	2	2	2	2	negative	negative
27	N027	2	25	negative	negative	negative	2	2	2	2	negative	negative
28	N028	2	49	negative	negative	negative	2	2	2	2	negative	negative
29	N029	2	24	negative	negative	negative	2	2	2	2	negative	negative
30	N030	2	47	negative	negative	negative	2	2	2	2	negative	negative
31	N031	2	40	negative	negative	negative	2	2	2	2	negative	negative
32	N032	2	47	negative	negative	negative	2	2	2	2	negative	negative
33	N033	2	56	negative	negative	negative	2	2	2	2	negative	negative
34	N034	1	53	negative	negative	negative	2	2	2	2	negative	negative
35	N035	2	56	negative	negative	negative	2	2	2	2	negative	negative
36	N036	2	52	negative	negative	negative	2	2	2	2	negative	negative
37	N037	2	50	negative	negative	negative	2	2	2	2	negative	negative
38	N038	2	41	negative	negative	negative	2	2	2	2	negative	negative
39	N039	2	43	negative	negative	negative	2	2	2	2	negative	negative
40	N040	2	40	negative	negative	negative	2	2	2	2	negative	negative
41	N041	2	43	negative	negative	negative	2	2	2	2	negative	negative
42	N042	2	49	negative	negative	negative	2	2	2	2	negative	negative
43	N043	2	43	negative	negative	negative	2	2	2	2	negative	negative
44	N044	1	44	negative	negative	negative	2	2	2	2	negative	negative
45	N045	1	36	negative	negative	negative	2	2	2	2	negative	negative
46	N046	2	25	negative	negative	negative	2	2	2	2	negative	negative
47	N047	2	24	negative	negative	negative	2	2	2	2	negative	negative

Supplementary Appendix 1. Raw Data from 100 Healthy Individuals as Negative Control

No.	Clinical Trial Healthy Control Number	Sex (Man=1, Woman=2)	Age (years)	NPS_PCR _Ct value E gene	NPS_PCR _Ct value RdRP gene	NPS_PCR _Ct value N gene	NPS_PCR_Result (Positive=1, Negative=2)	Antigen RDT [STANDARD Q COVID-19 Ag Saliva test (SD BIOSENSOR)] (Positive=1, Negative=2)	Antigen RDT [Gmate® COVID-19 Ag Saliva (Philosys)] (Positive=1, Negative=2)	Saliva_PCR _Result (Positive=1, Negative=2, Inconclusive=3)	Saliva_PCR _Ct value RdRP gene	Saliva_PCR _Ct value E gene
48	N048	1	29	negative	negative	negative	2	2	2	2	negative	negative
49	N049	2	24	negative	negative	negative	2	2	2	2	negative	negative
50	N050	1	26	negative	negative	negative	2	2	2	2	negative	negative
51	N051	1	26	negative	negative	negative	2	2	2	2	negative	negative
52	N052	2	32	negative	negative	negative	2	2	2	2	negative	negative
53	N053	2	25	negative	negative	negative	2	2	2	2	negative	negative
54	N054	2	28	negative	negative	negative	2	2	2	2	negative	negative
55	N055	1	55	negative	negative	negative	2	2	2	2	negative	negative
56	N056	1	30	negative	negative	negative	2	2	2	2	negative	negative
57	N057	1	31	negative	negative	negative	2	2	2	2	negative	negative
58	N058	1	31	negative	negative	negative	2	2	2	2	negative	negative
59	N059	1	58	negative	negative	negative	2	2	2	2	negative	negative
60	N060	2	59	negative	negative	negative	2	2	2	2	negative	negative
61	N061	2	41	negative	negative	negative	2	2	2	2	negative	negative
62	N062	2	30	negative	negative	negative	2	2	2	2	negative	negative
63	N063	1	39	negative	negative	negative	2	2	2	2	negative	negative
64	N064	1	29	negative	negative	negative	2	2	2	2	negative	negative
65	N065	1	38	negative	negative	negative	2	2	2	2	negative	negative
66	N066	1	32	negative	negative	negative	2	2	2	2	negative	negative
67	N067	2	27	negative	negative	negative	2	2	2	2	negative	negative
68	N068	1	31	negative	negative	negative	2	2	2	2	negative	negative
69	N069	2	55	negative	negative	negative	2	2	2	2	negative	negative
70	N070	2	33	negative	negative	negative	2	2	2	2	negative	negative
71	N071	1	39	negative	negative	negative	2	2	2	2	negative	negative

Supplementary Appendix 1. Raw Data from 100 Healthy Individuals as Negative Control

No.	Clinical Trial Healthy Control Number	Sex (Man=1, Woman=2)	Age (years)	NPS_PCR _Ct value E gene	NPS_PCR _Ct value RdRP gene	NPS_PCR _Ct value N gene	NPS_PCR_Result (Positive=1, Negative=2)	Antigen RDT [STANDARD Q COVID-19 Ag Saliva test (SD BIOSENSOR)] (Positive=1, Negative=2)	Antigen RDT [Gmate® COVID-19 Ag Saliva (Philosys)] (Positive=1, Negative=2)	Saliva_PCR _Result (Positive=1, Negative=2, Inconclusive=3)	Saliva_PCR _Ct value RdRP gene	Saliva_PCR _Ct value E gene
72	N072	2	40	negative	negative	negative	2	2	2	2	negative	negative
73	N073	2	24	negative	negative	negative	2	2	2	2	negative	negative
74	N074	2	38	negative	negative	negative	2	2	2	2	negative	negative
75	N075	1	54	negative	negative	negative	2	2	2	2	negative	negative
76	N076	2	45	negative	negative	negative	2	2	2	2	negative	negative
77	N077	2	45	negative	negative	negative	2	2	2	2	negative	negative
78	N078	2	33	negative	negative	negative	2	2	2	2	negative	negative
79	N079	1	58	negative	negative	negative	2	2	2	2	negative	negative
80	N080	2	31	negative	negative	negative	2	2	2	2	negative	negative
81	N081	2	48	negative	negative	negative	2	2	2	2	negative	negative
82	N082	2	48	negative	negative	negative	2	2	2	2	negative	negative
83	N083	1	50	negative	negative	negative	2	2	2	2	negative	negative
84	N084	2	39	negative	negative	negative	2	2	2	2	negative	negative
85	N085	2	38	negative	negative	negative	2	2	2	2	negative	negative
86	N086	2	27	negative	negative	negative	2	2	2	2	negative	negative
87	N087	2	23	negative	negative	negative	2	2	2	2	negative	negative
88	N088	2	36	negative	negative	negative	2	2	2	2	negative	negative
89	N089	2	26	negative	negative	negative	2	2	2	2	negative	negative
90	N090	1	44	negative	negative	negative	2	2	2	2	negative	negative
91	N091	2	28	negative	negative	negative	2	2	2	2	negative	negative
92	N092	2	26	negative	negative	negative	2	2	2	2	negative	negative
93	N093	1	28	negative	negative	negative	2	2	2	2	negative	negative
94	N094	1	60	negative	negative	negative	2	2	2	2	negative	negative
95	N095	2	60	negative	negative	negative	2	2	2	2	negative	negative

Supplementary Appendix 1. Raw Data from 100 Healthy Individuals as Negative Control

No.	Clinical Trial Healthy Control Number	Sex (Man=1, Woman=2)	Age (years)	NPS_PCR _Ct value E gene	NPS_PCR _Ct value RdRP gene	NPS_PCR _Ct value N gene	NPS_PCR_Result (Positive=1, Negative=2)	Antigen RDT [STANDARD Q COVID-19 Ag Saliva test (SD BIOSENSOR)] (Positive=1, Negative=2)	Antigen RDT [Gmate® COVID-19 Ag Saliva (Philosys)] (Positive=1, Negative=2)	Saliva_PCR _Result (Positive=1, Negative=2, Inconclusive=3)	Saliva_PCR _Ct value RdRP gene	Saliva_PCR _Ct value E gene
96	N096	2	51	negative	negative	negative	2	2	2	2	negative	negative
97	N097	1	58	negative	negative	negative	2	2	2	2	negative	negative
98	N098	1	40	negative	negative	negative	2	2	2	2	negative	negative
99	N099	1	42	negative	negative	negative	2	2	2	2	negative	negative
100	N100	2	58	negative	negative	negative	2	2	2	2	negative	negative

Supporting Information

Bean extract-based gargle for efficient diagnosis of active COVID-19 infection using rapid antigen tests

This appendix has been provided by the authors to give readers additional information about their work.

Supplement to: Joseph Kwon, Euna Ko, Se-Young Cho, et al. Bean extract-based gargle for efficient diagnosis of active COVID-19 infection using rapid antigen tests

TABLE OF CONTENTS

Study design ········ 4

Inclusion and exclusion criteria of study participants ········ 5

Supplementary Methods ········ 6

Chemicals and reagents ········ 6
Cryo-electron microscopy ········ 6
Enzyme-linked immunosorbent assay (ELISA) to test RBD binding inhibition to hACE2 receptors ········ 6
Preparation and Purification of TCan ········ 6
On-line Top-down Analysis of TCan using UPLC-ESI-QTOF (top-down) ········ 6
Protein analysis using Nano UPLC-QTOF for TCan analysis (bottom-up) ········ 7
N-Terminal sequencing of TCan ········ 7
Preparation of SARS-CoV-2 spike protein ········ 7
Circular dichroism spectroscopy ········ 7
Dynamic and light scattering measurement ········ 8
Intrinsic and extrinsic fluorescence spectroscopy ········ 8
Differential scanning calorimetry ········ 8
Isothermal titration calorimetry ········ 8
Size exclusion chromatography-combined multi-angle light scattering measurement ········ 9
Cell culture, viruses, and treatment ········ 9
Determination of cell viability ········ 9
Measurement of reactive oxygen species (ROS) levels ········ 9
Primary splenocyte preparation and culture ········ 9
Animals ········ 10
Experimental design for *in vivo* study ········ 10
Intratracheal and intravenous instillation ········ 10
Bronchoalveolar lavage fluid preparation ········ 10
Measurement of cytokine levels ········ 10
Histological analysis ········ 11

Supplementary Study Results and Discussion ········ 12

Supplementary Figures ········ 17

Figure S1. Comparison of Ct values from Beanguard gargle™- based RT-PCR (BG-RT-PCR) with NPS-based RT-PCR (NPS-RT-PCR) analysis in asymptomatic cases ········ 17
Figure S2. Comparison of the Ct values for BG-RT-PCR with those for NPS-RT-PCR ········ 18
Figure S3. Comparison of the sensitivity of BG-RT-PCR (A) and BG-Ag-RDT (B) depending on the Ct values of NPS-RT-PCR. ········ 19
Figure S4. Transmission electron micrographs (TEM) of HCoV-229E and SARS-CoV-2 with bean extract (BE) treatment ········ 20
Figure S5. Intermolecular interaction of bean extract (BE) and truncated canavalin (TCan) with SARS-CoV-2 ········ 21
Figure S6. HPLC profile of the purified TCan ········ 22
Figure S7. Multiple-charge electrospray mass spectra of TCan ········ 23
Figure S8. Molecular characterization of TCan using various biophysical approaches ········ 24
Figure S9. Structural characterization of TCan using spectroscopies ········ 25
Figure S10. Intramolecular characterization of TCan ········ 26
Figure S11. Irreversibility of the unfolding reaction of TCan after heat scanning ········ 27
Figure S12. Changes in the expression levels of inflammatory cytokines ········ 28
Figure S13. Effect of Con A and BE instillation on survival rate of C57BL/6 mice ········ 29
Figure S14. Changes in the levels of hepatic biomarker (AST, ALT, TBIL, and GGT) in response to Con A and BE instillation in mice ········ 30

Figure S15. Changes in lung and spleen weight-to-body weight ratio after Con A and BE instillation in mice ········ **31**
Figure S16. Changes of inflammatory cell numbers in bronchoalveolar lavage fluid (BALF) of mice following Con A and BE instillation ········ **32**
Figure S17. Changes in the expression of inflammatory cytokines ········ **33**
Figure S18. *In vitro* cytotoxicity of Con A and BE ········ **34**
Figure S19. Reactive oxygen species (ROS) level in cells after Con A and BE treatment ········ **35**

Supplementary Tables ········ **36**

Table S1. Evaluation of the diagnostic performance of BG-RT-PCR and BG-Ag-RDTs for patients with COVID-19 throughout the infection duration (n = 102) ········ **36**
Table S2. Results for the BG-RT-PCR and BG-Ag-RDTs from negative controls (n = 100) and COVID-19 patients within 6 days of illness (n = 45) ········ **37**
Table S3. Evaluation of the sensitivity of BG-RT-PCR and BG-Ag-RDTs for patients with SARS-CoV-2 Alpha and Delta variants within 6 days of illness ········ **38**
Table S4. Evaluation of the diagnostic performance of BG-RT-PCR and BG-Ag-RDTs for patients of SARS-CoV-2 variants within 6 days of illness ········ **39**
Table S5. Sensitivity of BG-Ag-RDTs depending on Ct value of positive BG-RT-PCR samples ········ **40**
Table S6. Summary of thermodynamic parameters for the structural stability of TCan using circular dichroism spectroscopy ········ **41**
Table S7. Summary of thermodynamic parameters for the structural stability of TCan using differential scanning calorimetry ········ **42**

Supplementary References ········ **43**

S1 text. Study design

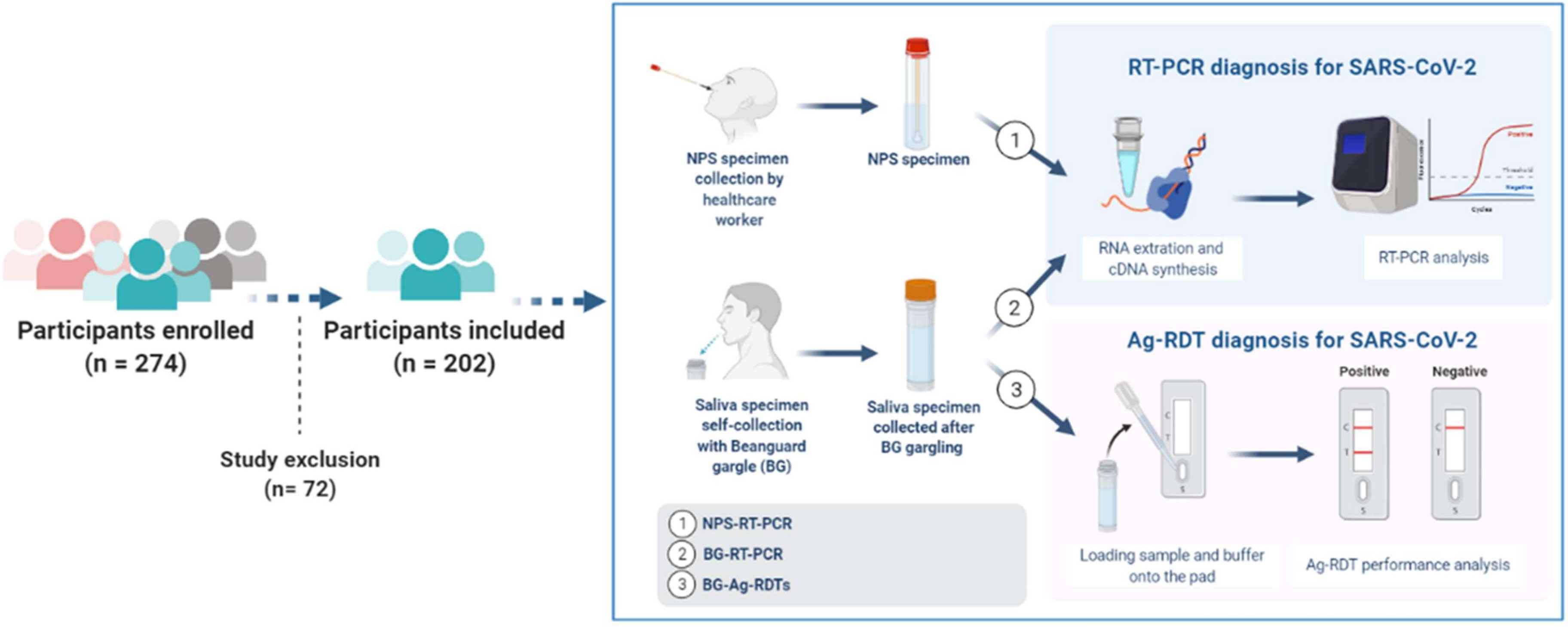

S2 text. Inclusion and exclusion criteria of study participants

Inclusion criteria

(a) Adults aged ≥ 18 years

(i) Patients hospitalized with COVID-19 confirmation in the screening center.

(ii) Healthy subjects with no previous contact with confirmed or suspected cases in the two weeks prior to the trial.

(b) Agree to participate in this trial and provide written informed consent.

(c) Permit the investigator to access the patient's medical records relevant to study procedures.

(d) Provide paired NPS and BG-based saliva specimen. Medical professionals collected NPS specimens from participants by having them spit into a tube after swirling and gargling 5 mL of BG for 2 min.

Exclusion criteria

(a) Healthy subjects with a body temperature higher than 37.0 ºC

(b) Healthy subjects with respiratory symptoms

(c) Not willing to provide sufficient BG-based saliva specimen.

(d) Not willing to collect NPS specimen.

S3 text. Supplementary Methods

Chemicals and reagents

Beanguard™ gargle (BG) was supplied by BIO3S, Inc. (Gwangju, Republic of Korea). Roswell Park Memorial Institute (RPMI) 1640, Dulbecco's modified Eagle's medium (DMEM), fetal bovine serum (FBS), trypsin-EDTA, phosphate-buffered saline (PBS), and streptomycin/penicillin were purchased from Gibco (New York, USA); 2′,7′ -dichloro-fluorescein diacetate (DCF-DA) was purchased from Thermo Fisher Scientific (California, USA); 1-anilinonaphthalene-8-sulfonate ammonium salt (ANS) was purchased from Sigma-Aldrich (St. Louis, MO, USA). Heat inactivated SARS-CoV-2 culture fluid (Isolate: Italy-INMI1) virus particles were obtained from Zeptometrix (Buffalo, NY). The SARS-CoV-2 spike (S) protein S1 was obtained from ACROBiosystems (Delaware, USA). Recombinant S2 was expressed and purified as described below. The concentrations of S1 and S2 were determined based on the UV absorbance at 280 nm with molar extinction coefficients of 91,775 and 42,455/ M cm, respectively, and were calculated using the ExPASy ProtParam tool (1).

Cryo-electron microscopy (cryo-EM)

The purified whole virus, SARS-CoV-2, and a mixture of SARS-CoV-2 and bean extract (BE) samples were inactivated with 2% paraformaldehyde overnight at 4 °C. Four microliters of each sample was applied to R1.2/1.3 Quantifoil holey carbon EM grids (200 mesh), which were glow-discharged for 60 s at 20 mA. The grids were then plunge-frozen in liquid ethane using a Vitrobot Mark IV (ThermoFisher Scientific) with 5 s of blotting in 100% humidity at 4 °C. Cryo-EM images were acquired using a Titan Krios (ThermoFisher Scientific), operated at 300 kV, and equipped with a Falcon III direct detector.

Enzyme-linked immunosorbent assay (ELISA) to test inhibition of RBD binding to hACE2 receptors

To assess the dissociation activity of the receptor binding domain (RBD)–human version of angiotensin-converting enzyme 2 (hACE2) complex by BE, COVID-19 Neutralizing Antibody ELISA Kit (Abnova, Taiwan) was used according to the manufacturer's instructions, with slight modifications. Briefly, the hACE2 protein was coated on 96-well ELISA plates at 4 °C overnight and unbound proteins were removed with washing buffer, followed by blocking for 1 h at 37 °C. For pre-treatment, RBD and BE solutions were incubated for 2 h at 37 °C before reaction with hACE2-coated plate. For post-treatment, COVID-19 Spike RBD Rabbit Fc-Tag protein was added to the plates and incubated for 2 h at 37 °C, followed by washing two times with each successive 2-fold dilution of BE solution. To detect bound RBD-Fc proteins in the plate, it was washed with a wash buffer provided in the ELISA kit, and horseradish peroxidase (HRP)-conjugated goat anti-rabbit antibody was added to each well and the plate was incubated for 1 h at 37 °C. The plate was then washed with wash buffer and subsequently treated with 3,3′,5,5′-tetramethylbenzidine (TMB). Color development was terminated using the stop solution in the ELISA kit. Absorbance at 450 nm was measured using a microplate reader (Bio Tek Co., USA). The percentage of RBD–hACE2 binding was calculated by measuring the differences in the amount of labeled RBD between the test and control samples.

Preparation and purification of TCan

For the preparation of truncated canavalin (TCan), sword bean (*Canavalia gladiata*) was proteolyzed by *Bacillus subtilis* at 33 °C for 4 days under conditions of dissolved oxygen (20–40% saturation) and pH 7.0. After proteolysis, the product was centrifuged (12,000 × g) at 4 °C for 15 min, and supernatant was collected and lyophilized. The final purification was performed by size exclusion chromatography (SEC) using a WTC-050S5 column (Wyatt Technology, Santa Barbara, CA) that was equilibrated with PBS (140 mM NaCl, 2.7 mM KCl, 6.5 mM Na_2HPO_4, 1.5 mM KH_2PO_4, pH 7.4). Pure protein was frozen in liquid nitrogen and stored at −80 °C. The SEC profile of TCan is shown in S5 Figure.

On-line top-down analysis of TCan using UPLC-ESI-QTOF (top-down)

On-line top-down MS was carried out on a waters ACQUITY UPLC I-Class system coupled to Synapt G2-S QTOF mass spectrometer (Waters Corp., USA). Briefly, 2 μL of purified TCan was injected into a 2.1 × 50 mm ACQUITY UPLC BEH300 C4 column (particle size 1.7 μm). The gradient was delivered at 400 μL/min, starting at 5% buffer B (0.1% formic acid in acetonitrile), then increased to 95% B at 6.7 min, and 5% B at 9.0 min. The mass spectrometer was operated in the ESI-Positive MS^E continuum data acquisition mode with the following conditions: capillary voltage 3.0 kV, cone voltage 50 V, desolvation gas 800 L/h, source temperature 120 °C, and desolvation temperature 300 °C. Data were collected at 1 spectra/second from 500–4,000 m/z. A maximum entropy algorithm (MaxEnt1) was used for mass deconvolution. Deconvolution was performed with a range of 1,500–2,600 Da around the expected mass, a target resolution of 0.5 Da, with 100 iterations of MaxEnt1. The

electrospray spectra of TCan is shown in Supplementary S6 Figure.

Protein analysis using Nano UPLC-QTOF for TCan analysis (bottom-up)

The purified TCan was digested using the "Tube-Gel digestion" protocol as described previously (2). LC-MS/MS was conducted according to a previous procedure (3). The tryptic peptide mixtures were dissolved with 0·5% trifluoroacetic acid prior to further analysis. A 5 μL dissolved sample was added onto a 100 μm × 2 cm nanoviper trap column and 15 cm × 75 μm nanoviper analysis column (Thermo Fisher Scientific) at a flow rate of 300 nL/min and were eluted with a gradient of 5%–40% acetonitrile over 95 min. All MS and MS/MS spectra capturedby the Q Exative Plus mass spectrometer (Thermo Fisher Scientific) were acquired in the data-dependent top 12 mode. The MS/MS data were analyzed using MASCOT 2·7, with a parameter corresponding to a false discoveryrate (FDR) of 1%. One missed cleavage was allowed for identifying the peptides from TCan.

N-Terminal sequencing of TCan

For N-terminal amino acid sequencing, the protein samples were transferred from an SDS–PAGE gel to a PVDF membrane and the transferred protein band on the membrane was analyzed using the LC 492 Protein Sequencing System (Applied Biosystems Instruments, USA).

Preparation of SARS-CoV-2 spike protein

The gene encoding S2 of the SARS-CoV-2 spike protein (accession number QKU5385.1, amino acid residues 686–1213) was codon-optimized for protein expression in *Nicotiana benthamiana*, synthesized by GenScript Biotechnology Corp. (Nanjing, China) and cloned into a pCAMBIA1300 vector with an 8 histidine tag, an ER retention signal HDEL, and a Cor1 sequence. *N. benthamiana* leaves (fresh weight) expressing recombinant S2 were harvested and homogenized using a blender in the presence of an extraction buffer (25 mM Tris-HCl [pH 8.0], 300 mM NaCl, 10 mM imidazole, 5% glycerol, 1% Sarkosyl, 10 mM H_2O_2, and 1 mM PMSF). To remove debris, the extracts were centrifuged at 20,000 g for 40 min, and supernatants were filtered through Miracloth (EMD Millipore Corp., Billerica, MA, USA). The resulting supernatant was loaded onto a 100-mL Ni-IDA agarose column (Bio-Rad, Seoul, Republic of Korea). The column was washed with a washing buffer consisting of 25 mM Tris-HCl (pH 8.0), 300 mM NaCl, and 10 mM imidazole. S2 was eluted with the same buffer containing 30–300 mM imidazole. The proteins were further purified using gel-filtration chromatography (Superdex 200 Increase 10/300 GL, GE Healthcare, USA), pre-equilibrated with phosphate-buffered saline (PBS), and stored at -80 °C.

Circular dichroism spectroscopy

Circular dichroism (CD) measurements were performed on a JASCO J-1500 spectrophotometer (JASCO, Japan) using a quartz cuvette with 0·1-cm path length. Samples were prepared using 0.25 mg/mL of TCan in 20 mM sodium phosphate buffer (pH 7·5). Far-UV CD spectra from 195 to 250 nm were obtained at a scanning rate of 200 nm/min. CD signals were expressed as mean residue ellipticity [θ] (degrees cm^2/dmol) (4). For the thermal scans, far-UV CD spectra were acquired every 5 °C from 5 to 100 °C at a heating rate of 1 °C min^{-1}. Then, monitoring CD signals at 204 nm was carried out by decreasing the temperature from 100 to 5 °C at a rate of 10 °C/ /min. The temperature was controlled using an RW-0525G low-temperature bath circulator (Lab Companion, Republic of Korea).

The melting temperature (${}^{CD}T_m$), enthalpy change (${}^{CD}\Delta H$), and heat capacity change (ΔC_p) for the heat denaturation of folded TCan were obtained by regression analysis as follows (5):

$$\theta = \frac{(a - c) + (b - d)}{1 + \exp\left(-\frac{{}^{CD}\Delta H_m({}^{CD}T_m)\left(1 - \frac{T}{{}^{CD}T_m}\right) - \Delta C_p\left({}^{CD}T_m - T + T\ln\frac{T}{{}^{CD}T_m}\right)}{RT}\right)} + (c + dT)$$

where θ is the signal intensity monitored by CD. The pre- and post-unfolding baselines are described by a + bT and c + dT, respectively. T is the temperature in Kelvin, and R indicates the gas constant. ${}^{CD}T_m$ is the midpoint temperature of the thermal denaturation of TCan, ΔC_p is the heat capacity change between pre- and post-unfolded TCan, and ${}^{CD}\Delta H$ (${}^{CD}T_m$) is the enthalpy change at ${}^{CD}T_m$. CD spectra were analyzed using the BeStSel algorithm (6) to estimate the content of the secondary structure. The concentration of TCan was determined using the UV absorbance and a molar extinction coefficient of 8,940/M cm at 280 nm.

Dynamic and light scattering measurement

Dynamic light scattering (DLS) and static light scattering measurements of TCan in 20 mM sodium phosphate buffer (pH 7.5) were performed using a DynaPro Plate Reader II (Wyatt Technology Corp., USA) and a Cary Eclipse fluorescence spectrophotometer (Agilent Technologies, USA) at 25 °C, respectively. For DLS measurements, sample solutions containing 11·7 μM TCan before and after heat treatment were subjected to centrifugation at 10,000 rpm for 5 min, and the supernatants were loaded into a 384-well plate. The average hydrodynamic radius (R_h) of the 20 measurements was obtained. Experimental data were processed and analyzed using the DYNAMICS software (Wyatt Technology Corp., ver. 7.0). The emission spectrum of light scattering of approximately 12 μM TCan was recorded from 300 to 400 nm with an excitation wavelength of 350 nm. The slit widths of the excitation and emission wavelengths were 10 and 5 nm, respectively. Scattering of 20 mM sodium phosphate buffer (pH 7.5) was also performed as a control using the same method.

Intrinsic and extrinsic fluorescence spectroscopy

All fluorescence measurements were carried out in the absence and presence of 6.0 M Urea using a Cary Eclipse fluorescence spectrophotometer (Agilent Technologies, USA) and approximately 11.7 μM TCan in 20 mM sodium phosphate buffer (pH 7·5) at 25 °C. Intrinsic fluorescence spectra were obtained by exciting phenylalanine and tyrosine/tryptophan at 240 and 280 nm, respectively. An excitation slit of 10 nm and an emission slit of 20 nm were used. ANS (50 μM) was utilized as an extrinsic fluorophore, and its emission spectrum was recorded from 370 to 600 nm with an excitation wavelength of 370 nm. The concentration of ANS was determined using UV absorbance at 350 nm and a molar extinction coefficient of 4,950/M cm. The emission spectra were monitoredfrom 370 nm to 600 nm. The excitation and emission slit widths for intrinsic and extrinsic fluorescence were bothset to 20 nm. Fluorescence measurements of 20 mM sodium phosphate buffer (pH 7·5) were carried out as a control using the same method.

Differential scanning calorimetry

Differential scanning calorimetry (DSC) experiments of 17.6 μM TCan in 20 mM sodium phosphate buffer (pH 7·5) were carried out using a PEAQ-DSC instrument (Malvern Panalytical, UK). The TCan solution was heated from 20 °C to 100 °C at a heating rate of 1 °C/min. After subtraction of the buffer baseline and normalization with protein concentrations, fitting analyses were performed based on the non-two-state transition using PEAQ-DSC analysis software (Malvern Panalytical, UK). The fit equation used was based on the Levenberg-Marquardt nonlinear least-squares method (7).

$$C_p(T) = \frac{K(T)\,{}^{DSC}\Delta H_{VH}\,{}^{DSC}\Delta H_{cal}}{(1 + K(T))^2 RT^2} + \cdots$$

$$(T) = \exp\left[\frac{-{}^{DSC}\Delta H_{VH}}{RT}\left(1 - \frac{T}{{}^{DSC}T_m}\right)\right]$$

where T_m is the thermal midpoint of a transition, as described above. The change in calorimetric enthalpy (${}^{DSC}\Delta H_{cal}$) was determined using the DSC peak area, and the enthalpy change was based on the van't Hoff method (${}^{DSC}\Delta H_{VH}$) and was estimated by exploiting the shape of the DSC peak, that is, the slope of the transition curve between folded and unfolded proteins. $C_p(T)$ was calculated at any temperature T in Kelvin. $K(T)$ indicates the equilibrium constant as a function of the temperature.

Isothermal titration calorimetry

Isothermal titration calorimetry (ITC) was performed using a MicroCal PEAQ-ITC (Malvern Panalytical, UK) at 37 °C. All samples, TCan, S1, and S2, were prepared in 20 mM sodium phosphate buffer (pH 7·5) containing 100 mM NaCl. TCan (287.6 μM) in an ITC syringe was titrated to solutions containing either S1 or S2 at 17 μM in anITC cell with continuous stirring at 750 rpm. The total number of titrations was 19 with 150-s intervals between titrations. The initial delay and reference power were set to 60 s and 10 μcal/s, respectively. The dilution heat of TCan was also measured using the same method and the ITC parameters. Binding isotherms after the subtraction of dilution heat and the baseline correction were fitted to a one-set of sites binding model (8):

$$Q = \frac{n[\text{S protein}]_t\,{}^{ITC}\Delta H_{bind} V_0}{2}\left[1 + \frac{[\text{TCan}]_t}{n[\text{S protein}]_t} + \frac{K_d}{n[\text{S protein}]_t} - \sqrt{\left(1 + \frac{[\text{TCan}]_t}{n[\text{S protein}]_t} + \frac{K_d}{nK[\text{S protein}]_t}\right)^2 - \frac{4[\text{TCan}]_t}{n[\text{S protein}]_t}}\right]$$

where Q is the total heat constant, and n indicates the number of TCan molecules which bind to one of the S proteins (*i.e.*, S1 and S2). ${}^{ITC}\Delta H_{bind}$ is the change in enthalpy for the intermolecular interactions. V_0 is an active cell volume which considers the volume of the ITC cell and the volume increased by titration. The total concentrations of S proteins and TCan are displayed as $[\text{S protein}]_t$ and $[\text{TCan}]_t$, respectively, at any given time (t). The dissociation constant is denoted as K_d. In binding isotherms of TCan-S1 and TCan-S2, a best fit was obtained without fixation of variable, and fit by fixing a value of K_d was also performed.

Size exclusion chromatography-combined multi-angle light scattering measurement

Size exclusion chromatography with inline multi-angle light scattering (SEC-MALS) measurements were conducted using an instrument from Wyatt Technology (USA). Freshly purified 94·4 μM TCan solution was loaded onto a WTC-050S5 column (Wyatt Technology Corp., USA) with PBS running buffer. The flow rate was set to 0·5 mL/min. MALS and UV spectra were recorded using a DAWN HELEOS-II (Wyatt Technology Corp., USA), and Optilab T-rEX (Wyatt Technology Corp., USA) was used to determine the differential refractive index. SEC-MALS results were analyzed using Astra 6·1 software (Wyatt Technologies Corp., USA). A differential refractive index increment (dn/dc) value of 0.185 was used.

Cell culture, viruses, and treatment

To compare the cytotoxicity of BE on different cell types, two human hepatocellular carcinoma cell lines (HepG2 and Huh7 cells) and two lung origin cell lines (adenocarcinomic human alveolar basal epithelial cells, A549, and alveolar macrophages, MH-S), were purchased from the American Type Culture Collection (ATCC, Manassas, VA, USA). A549 cells were cultured in RPMI 1640 containing 5% FBS and 1% streptomycin/penicillin at 37 °C in an atmosphere containing 5% CO_2. MH-S cells were cultured in RPMI 1640 containing 10% FBS and 1% streptomycin/penicillin at 37 °C in an atmosphere containing 5% CO_2. HepG2 and Huh7 cells were cultured in DMEM containing 10% FBS and 1% streptomycin/penicillin at 37 °C in an atmosphere containing 5% CO_2. Using sterile 24-well culture plates, cells were seeded at a concentration of 2×10^5 in 500 μL of each medium and treated with Con A or BE at 5, 10, 25, 50, 100, 200, and 400 ppm.

Determination of cell viability

After 6 h of BE treatment, A549, HepG2, MH-S, and Huh7 cells were harvested using 0.25% trypsin-EDTA and counted using a NucleoCounter (NC-250, ChemoMetec, Gydevang, Denmark), according to the manufacturer's protocol. Cell viability was calculated as the ratio of live cells to total cells. Inhibitory concentration (IC) was also calculated from nonlinear regression of concentration-response inhibition curves using GraphPad Prism v·7 (GraphPad Software, San Diego, CA).

Measurement of reactive oxygen species (ROS) levels

A549, HepG2, MH-S, and Huh7 cells were seeded and treated with BE, as previously described. The treated cells were incubated for 30 min at 37 °C in culture medium containing 3.3 μmol/L DCF-DA for ROS detection, and the cells were washed with PBS. DCF-DA intensity in the cells was immediately measured using flow cytometry (CytoFLEX, Beckman Coulter, Brea, CA, USA). ROS production in the cells is represented as a percentage of DCF-DA intensity relative to the naïve control, which was defined as 100%.

Primary splenocyte preparation and culture

Primary splenocytes were isolated from BALB/c and C57BL/6 mice. Single cell suspensions were obtained by mincing the spleen and gently pressing the fragments through a 45 μm nylon cell strainer (BD Falcon, Bedford, MA, USA). The suspension was mixed with 1× RBC lysis buffer (eBioscience Inc., San Diego, CA, USA) for 5 min at room temperature. For primary culture, the spleen cells were resuspended in RPMI 1640 containing 5% FBS, 100 U/mL penicillin, and 100 μg/mL streptomycin. Using sterile 24-well culture plates, cells were seeded at a density of 3×10^6 cells in 500 μL of medium. Spleen cells were cultured for 24 or 48 h in the presence of 2.5 μg/mL Con A in a humidified atmosphere of 5% CO_2 and 95% air. Cellular morphology was observed under a light microscope (20×; Leica Microsystems, Wetzlar, Germany). Cells were harvested and centrifuged at 2,000 × *g* for 5 min; thereafter, the supernatants were collected and stored at −70 °C until measurement of inflammatory

cytokine levels by ELISA.

Animals
Male C57BL/6 mice (Orient Bio Ltd, Seongnam, Korea) were housed under 12 h light/dark cycles in temperature-controlled (22 ± 30 °C) rooms. Throughout the experiments, they had *ad libitum* access to standard laboratory chow and tap water. The experiments were performed in accordance with the protocols approved by the Institutional Animal Care and Use Committee of the Korea Institute of Toxicology (No. 2001–0007, 2005–0163, and 2008–0244).

Experimental design for *in vivo* study
The following three studies were conducted to compare the toxic effects of BE:
Study 1) Effect of high-dose BE acute toxicity on survival rate
Study 2) Comparison of BE hepatotoxicity
Study 3) Comparison of BE respiratory toxicity to determine the possibility of intratracheal instillation as a mode of administration to measure the antiviral efficacy of BE

Study 1: To examine and compare the effect of BE on survival rate, seven groups were created (n = 3 per group), *i.e.*, vehicle control group, Con A treatment groups (40, 80, and 160 mg/kg), and BE treatment groups (75, 150, and 200 mg/kg). BE was diluted in saline to create equivalent doses, which were intratracheally administered using a modified automatic video instillator (Doobae System, Seoul, South Korea). Following Con A and BE administration, the survival rate was monitored for 24 h.
Study 2: To examine and compare the hepatotoxicity of Con A and BE, mice were administered Con A and BE intravenously (5 and 15 mg/kg) and intratracheally (10, 20, and 40 mg/kg). Several parameters were then evaluated, including serum alanine aminotransferase (ALT), aspartate aminotransferase (AST), total bilirubin (TBIL), and γ-glutamyl transferase (GGT) levels in the blood, as well as histological changes in the liver.
Study 3: To examine and compare the toxicity of Con A and BE on the lungs, mice were intratracheally administered different concentrations of Con A or BE, *i.e.*, 1, 2.5, 5, and 10 mg/kg. Organ weight, cellular changes, and protein levels of inflammatory cytokines in bronchoalveolar lavage fluid (BALF), as well as histological changes in the lung and spleen tissues were assessed 24 h after the single administration of Con A or BE.

Intratracheal and intravenous instillation
For intratracheal instillation, mice were anesthetized by inhalation. Prior to instillation, isoflurane was delivered into the induction chamber using a small animal porTable anesthesia system (L-PAS-02, LMSKOREA, Inc., Seongnam, South Korea) equipped with an isoflurane vaporizer. The mice were then exposed to 2·5% isoflurane delivered in O_2 (2 L/min) within the induction chamber until a sleep-like state was reached. Mice receiving isoflurane anesthesia were removed from the induction chamber, and instillation was performed immediately. For intravenous instillation, mice were placed in a murine restraining device. The tail vein was dilated by soaking the tail in warm (e.g., at 30–35 °C) water for 1 to 2 min. After swabbing with 70% alcohol, the tail veins were injected with the designated concentration. Mice were subsequently transferred to their cages.

Bronchoalveolar lavage fluid preparation
Twenty-four hours after Con A and BE instillation, mice were anesthetized and the left lung was ligated, while the right lungs were gently lavaged three times via a tracheal tube with a total volume of 0.7 mL PBS. The total number of cells in the collected bronchoalveolar lavage fluid (BALF) was counted using a NucleoCounter. For differential cell counts, BALF cell smears were prepared using Cytospin (Thermo Fisher Scientific) and stained with the Diff-Quik solution (Dade Diagnostics, Aguada, Puerto Rico), followed by counting of different cell types (n = 200/slide). BALF was immediately centrifuged at 2,000 × *g* for 5 min, and the collected supernatant was stored at −70 °C until measurement of cytokine levels by ELISA.

Measurement of cytokine levels
Interleukin (IL)-4, IL-5, IL-13, IL-17, IL-1β, IFN-γ, and tumor necrosis factor alpha (TNF-α) levels in BALF and cell supernatants were quantified by ELISA using commercial kits (Thermo Fisher Scientific), according to the manufacturer's protocols. The sensitivity for IL-4, IL-5, IL-13, IL-17, IL-1β, IFN-γ, and TNF-α assays were 0.32, 3.3, 2.8, 1.2, 5.3, and 3.7 pg/mL, respectively. The intra- and inter-assay coefficients of variation for IL-4, IL-5, IL-13, IL-17, IL-1β, IFN-γ and TNF-α were 7.1% 7.9%, 5%, 4.7%, <5.0% and 6.5%, and 10%, 4.6%, 5%, 5.7%, < 10% and 5.7%, respectively.

Histological analysis

Twenty-four hours after BE instillation, the mice were euthanized for histological analysis. Lung and liver tissues were removed and fixed in 10% (v/v) neutral-buffered formalin, dehydrated, embedded in paraffin, and cut into 4 μm sections. They were then deparaffinized with xylene and stained with hematoxylin and eosin (Sigma-Aldrich). Stained sections were analyzed using a light microscope (Axio Imager M1; Carl Zeiss, Oberkochen, Germany). The degree of inflammation was scored on a scale of 0–4.

S4 text. Supplementary Study Results and Discussion

Characterization of TCan

In this work, fermented sword bean (*Canavalia gladiata*) was used and the active ingredient TCan was successfully produced in culture medium during the cultivation. Interestingly, N-terminal sequence of the canavalin was determined by Edman degradation as ^{242}LSSQDKPFN^{251}, indicating that in *C. gladiata* truncation occurs at the N-terminal region of canavalin. To determine the mass value of the whole protein, the multiple-charge electrospray mass spectrum (S7 Fig) of TCan protein was deconvoluted to calculate protein molecular weight by MaxEnt1 software. The mass value of the TCan protein was observed to be 21,176 Da, which was predicted to be due to truncation of not only the N-terminal region but also the C-terminal lysine residue (190). The peptides of the protein were identified using Q Exative Plus mass spectrometer. A protein identification search revealed that the peptides present in the predicted TCan matched with those of canavalin (*C. gladiata*, P10562·1), providing 97% sequence coverage for the predicted TCan amino acid sequence using LC-MS/MS bottom-up analysis. In future, it is necessary to verify the biochemical characterization of TCan by designing a recombinant protein.

Structural characterization of TCan

To understand the molecular properties of TCan, we first performed a structural investigation using circular dichroism (CD) (S8A Fig) and fluorescence spectroscopic techniques (S8B–D Fig). Far-UV CD spectroscopy has been extensively used to obtain information on the secondary structures of proteins based on the intrinsic pattern of a spectrum (9). Intrinsic and extrinsic fluorescence have been shown to be capable of characterizing tertiary structures around fluorophores based on the wavelength at the maximum intensity of fluorescence emission and emission intensity (10).

Far-UV spectra at 25 and 37 °C showed a similar pattern, with a minimum at ~215 nm and a maximum at ~200 nm, suggesting a mixture of helical and β structures in a folded state at both temperatures (S8A and 10A Figs). Further analyses of the secondary structures indicated that TCan consists of several types of secondary structures, and β structures show a higher proportion than helical structures by ~20% (S8D Fig). These results are in line with the three-dimensional (3D) structure of TCan determined by X-ray crystallography (S9E and F Fig) (9,11): TCan is mainly composed of two antiparallel β-sheets containing 13 β-strands (β1–β13) and four helices (α1–α4) (S10A and D Fig). The 3D structure corresponding to TCan in an intact canavalin (PDB ID:6v7j) is shown in the figure.

Next, three-dimensionally folded structures of TCan were examined using 1-anilino-8-naphthalenesulfonate (ANS) as an extrinsic fluorophore and phenylalanine (Phe) and tyrosine (Tyr) as intrinsic fluorophores (10). ANS is a well-known probe for clarifying partially unfolded structures such as a molten-globule structure, which has the same secondary structure as a native structure without a solid 3D conformation. Thus, binding of ANS to a partially unfolded structure increases its fluorescence emission, that is, a red shift. Although a subtle change was observed, ANS spectra of TCan in the absence and presence of 6 M urea, a chemical denaturant, were similar to the spectrum of the buffer, indicating that ANS did not bind to TCan in either conditions and that TCan has a 3D structure (S9B Fig). The Phe fluorescence spectrum in the absence of urea showed a weak intensity at ~310 nm (S9C Fig), suggesting that the six Phe residues were not exposed to polar solvents and were buried in cores, as indicated in the 3D structure (S9E Fig). The similar spectra at 0 and 6 M urea implied that physicochemical environments around Phe residues are not remarkably different in the folded and urea-induced unfolded states. Similarly, the Tyr fluorescence spectrum without urea exhibited a weak maximum emission at ~310 nm (S9D Fig)—similar to that with 6 M urea—demonstrating that five Tyr residues are buried in the cores (S9F Fig) and are located in a similar environment, regardless of urea.

To explore the internal dynamic structures and core regions, we examined X-ray *B*-factors and the hydrophobicity of each residue in TCan (S10 Fig). Higher values of the *B*-factor have been interpreted as higher flexibility of atoms and/or residues, and residues with higher hydrophobicity are often buried in the core regions of folded proteins (9,12). Most *B*-factors ranged between 40 and 60 Å^2, and β-structures showed a tendency of lower *B*-factors than those of helical structures (S10A Fig). Random coil-like structures, including both terminal parts and loops between structured secondary structures, exhibited high *B*-factors. The loop region between the two β-sheets also showed a high number of *B*-factors. In contrast, N- (~β_1-~α_1) and C- terminal parts (~β_{12}-~β_{13}) and the loop region between the two β-sheets showed low hydropathy scores, whereas the two central regions, ~β_4-~β_7 and ~β_8-~β_{11}, showed high hydropathy scores (S10B and D Fig). Interestingly, these two regions individually consisted of a β-sheet and displayed low *B*-factors, revealing a hydrophobic core of TCan (S10D Fig).

Considering these results together, we can conclude that TCan, a truncated form of canavalin, has a secondary and tertiary structure similar to the native structure of the globular protein. Hydrophobic cores of the two β-sheets with low flexibility are capped by two helical regions (~α_1 in the N-terminal part and ~α_2~α_4 in the C-terminal part)

and most likely contribute to holding a stereo-structure.

Characterization of conformational stability of TCan

To utilize TCan as a biomaterial and diagnostic agent, it is vital to confirm conformational stability. Therefore, we examined the thermal stability of TCan using spectroscopy and calorimetry. Far-UV CD spectra did not change significantly in the temperature range between 10 and ~70 °C; however, at temperatures over ~70 °C, there were marked changes in the spectra (S8A Fig). The disappearance of a positive CD signal at ~200 nm and a simultaneous increase in the magnitude of the negative CD intensity at ~205 nm suggested the disruption of secondary structures, which is often accompanied by global or sub-global unfolding of 3D structures. Tracing of the CD intensity at 204 nm with an increase in temperature manifested a clear transition of heat-induced unfolding of TCan (S8B Fig). The CD intensity decreased from ~70 °C due to thermal denaturation and was saturated at ~80 °C, which indicated that all TCan thermally unfolded. A sharp transition was detected, indicating cooperative unfolding due to the high packing density interior of TCan, which comes from its high hydrophobicity and low flexibility. This revealed that TCan has a 3D structure as a stable globular protein.

In accordance with the CD results, differential scanning calorimetry (DSC) displayed a positive DSC peak in the thermogram, revealing thermal unfolding of TCan by uptake of heat for denaturation (S9C Fig). An endothermic DSC peak appeared from ~70 °C and was at the level of the baseline from ~70 °C. Temperature-dependent changes in the secondary structure suggested that decreases in the content of β-structures and increases in random coil conformations were followed by thermal unfolding of TCan (S8D Fig). We believe that the hydrophobic cores of β-sheets essential for structural stability were disrupted due to heat stress.

With the assumption of a reversible reaction, we further carried out quantitative analyses of the conformational stability of TCan based on two-state unfolding. The melting temperature ™, at which unfolded and folded populations of TCan were identical, and the change in enthalpy (ΔH) for unfolding were obtained using CD (${}^{CD}\Delta H$) and DSC (${}^{DSC}\Delta H$) (S6 and 7 Tables). Tm values calculated from CD (${}^{CD}T_m$) and DSC (${}^{DSC}T_m$) were ~75 and ~76 °C, respectively, revealing a relatively high T_m as a number of proteins showed T_m lower than 70 °C (13). CD analyses with the best fit also produced an endothermic ${}^{CD}\Delta H$ of ~100 kcal/mol and a change in heat capacity (ΔC_p) of ~2 kcal/mol (S6 Table), which falls into the range of values of globular proteins (14). Hydration of the hydrophobic cores would cause a positive ΔC_p value. ΔH obtained using the van't Hoff method and DSC thermogram (${}^{DSC}\Delta H_{VH}$) was ~160 kcal/mol (S7 Table), which was higher than that from CD; however, it was still in the range of ΔH for globular proteins.

The reversibility of the thermal unfolding of TCan was tested using several approaches (S11 Fig). The far-UV CD spectrum after heat scanning was not superimposed, and a spectrum indicating an unfolded structure remained (S11A Fig). The ANS spectrum of heat-treated TCan manifested an increased fluorescence emission with a red shift, indicative of the exposure of hydrophobic regions of TCan with partial unfolding (S11B Fig). The intrinsic fluorescence spectra of Phe (S11C Fig) and Tyr (S11D Fig) of TCan subjected to heat treatment did not exhibit appreciable changes. The distribution of the hydrodynamic radius (R_h) of TCan after heat incubation was wider than that before heat scanning (S11E Fig). The average Rh was 3.76 nm; however, new molecular species with higher and lower molecular weights appeared to form. A decrease in the light scattering intensity also signified different structural states of TCan depending on the thermal scan (S11F Fig). These results indicate that thermally unfolded TCan cannot refold to its original structure; that is, unfolding of TCan is irreversible.

In summary, a systematic investigation revealed the conformational stability of TCan with detailed thermodynamic properties. Although thermal unfolding of TCan is irreversible, it has been demonstrated that TCan with well-packed hydrophobic cores and low flexibility is a fully stable protein for biological and diagnostic applications.

Investigation on oligomeric states of TCan

The association states of proteins are important for understanding the underlying mechanism of the stability and intermolecular recognition of proteins for functional, biological, and diagnostic applications. In this regard, we focused on the colloidal and association states of TCan. Light scattering has been used to examine the association and colloidal states of proteins. The scattering intensity of TCan is larger than that of the buffer (S8E Fig), suggesting that TCan may exist as a multimer. Dynamic light scattering (DLS) revealed a monodisperse R_h with an average Rh of 3.95 nm (S8F Fig). Based on the relationship between the number of residues, protein volume, and Rh (15), it was calculated that approximately six TCan monomers form the oligomers. Size exclusion chromatography with inline multi-angle light scattering (SEC-MALS) also elucidated the oligomeric states of TCan (S8G Fig). Considering that the molecular weight of the TCan monomer is 21,100, the molecular weight of ~140,000 obtained using SEC-MALS indicated that approximately seven Tcan monomers interact intermolecularly to form an oligomer. Furthermore, consistent with DLS and MALS analyses, DSC also showed the oligomeric states of Tcan (S7 Table). We used the fact that the ratio of ${}^{DSC}\Delta H_{VH}$ / ${}^{DSC}\Delta H_{cal}$ can be a criterion

for the oligomeric state of proteins (16). As the ratio of $^{DSC}\Delta H_{VH}$ (158.0 ± 0.7) / $^{DSC}\Delta H_{cal}$ (19.3 ± 0.1) was 8.3 ± 0.1, it was conceived that a Tcan oligomer accommodates approximately eight monomers.
Taken together, TCan forms an oligomer containing ~6 to ~8 monomers under physiological conditions. We rationalized that Tcan efficiently uses spontaneous oligomerization by escaping misfolding and aggregation which inactivates TCan. Furthermore, because intermolecular associations such as dimerization and a higher order of oligomerization often strengthen conformational stability (17), oligomer formation contributes to the relatively high stability of TCan against external stresses in addition to internal stabilizing factors, as mentioned above. Oligomerization of Tcan may also be beneficial to protect itself from external perturbations, such as proteolysis, and be functional.

Intermolecular interactions between Tcan and spike proteins
The binding capability of Tcan for the two SARS-CoV-2 spike proteins, S1 and S2, was investigated using isothermal titration calorimetry (ITC). ITC is one of the most powerful and accurate methodologies for studying intermolecular interactions without labeling or immobilization (18–20). It can elegantly clarify both weak and strong intermolecular interactions by detecting even a small amount of reaction heat.
Titration of Tcan to either S1 (S5D Fig, left panel) or S2 (S5D Fig, right panel) produced energetically favorable exothermic heat, revealing interprotein interactions between TCan and spike proteins. The reaction heat of TCan-S1 was greater than that of TCan-S2, and, thus, the magnitude of the negative enthalpy change for binding ($^{ITC}\Delta H_{bind}$) of TCan-S1 (~-6—~-7 kcal/mol) seemed to be larger than the $^{ITC}\Delta H_{bind}$ of TCan-S2 (~-0·8 kcal/mol). The two binding systems did not show saturation at a molar ratio of 3, indicating that intermolecular interactions are not strong. Although binding isotherms were not saturated, we attempted to evaluate the apparent dissociation constant (K_d). The apparent K_d values for the TCan-S1 and TCan-S2 interactions were in the range of ~20—~100 μM and ~10—~80 μM, respectively. These findings suggest that TCan moderately binds to S1 and S2 with similar affinity. Given the similar interprotein affinity, TCan may bind and recognize a spike protein in a different thermodynamic way: the TCan-S1 interaction is more enthalpically driven, whereas entropy is a predominant player in the TCan-S2 interaction. It should be noted that ITC results, in addition to information on oligomeric states (S5D Fig and S7 Table), collaborate with the Cryo-EM and TEM image (Fig 2A–D and S4 Fig) which demonstrated binding of several TCan, that is, TCan oligomers, to the surfaces of HCoV-229E and SARS-CoV-2 through S1 and S2.

Mitogenic response induced by Con A and BE in mice splenocytes
Con A is well-known for inducing the mitogenic response in splenocytes that activate the immune system, recruit lymphocytes, and elicit cytokine production (21). To compare the mitogenic effects induced by Con A and BE, we investigated T helper (Th) cytokine (IFN-γ for Th1, IL-17 for Th17, and IL-4, IL-5, and IL-13 for Th2 cells) production by treating splenocytes with Con A or BE at a concentration of 2.5 μg/mL (S12 Fig). The results showed that compared to BE, Con A induced significantly higher production of all Th cytokines. Although production of IFN-γ, IL-17, IL-5 and IL-13 was increased in BE-treated splenocytes over the duration of the experiment, the production was significantly lower than that in the Con A-treated group.

Effects of Con A and BE on survival rate of mice
To examine and compare the toxic effects of Con A and BE in a living system, we primarily investigated the effects of Con A and BE on mortality after intratracheal instillation in C57BL/6 mice (S13 Fig). The survival rate was monitored every hour in C57BL/6 mice divided into seven groups: vehicle control (VC), Con A-treated at the concentration of 40, 80 and 160 mg/kg body weight (BW) (Con A 40, Con A 80, Con A 160 groups), and BE-treated at the concentration of 75, 160 and 200 mg/kg BW (BE 75, BE 160, BE 200 groups) for 24 h. The VC and BE-treated groups exhibited a survival rate of 100%, while the Con A-treated groups exhibited a decrease in survival. The survival rate for the mice in the Con A-treated groups began to decrease from the 17th h of treatment, and no survival of mice in the Con A 160 group was observed by the 24th h of treatment. Meanwhile, 60% survival was observed in mice in the Con A 40 and Con A 80 groups after 24 h of treatment. These results indicate that BE was not toxic to mice, which was consistent with the *in vitro* cytotoxicity results.

Effects of Con A and BE on liver toxicity of mice
Con A can activate T cells to secrete cytokines that cause liver injury (22). To compare the effects of Con A and BE on liver injury, the levels of serum AST, ALT, TBIL, and GGT, which are the biomarkers of liver function, in mice were evaluated at 24 h after Con A and BE administration (S14 Fig). The result showed that the mode of administration impacted hepatic biomarker levels. Although, administration by intratracheal instillation of both

Con A and BE-treated mice did not significantly affect the level of hepatic biomarkers, the high concentration of Con A (15 mg/kg BW) administered intravenously remarkably increased the expression of (p value < .05) all biomarkers.

Effects of Con A and BE on lung injury of mice

Animal experiments were conducted to compare lung toxicity by direct respiratory exposure to Con A and BE. The following parameters were analyzed to assess lung injury: (i) lung and spleen organ weight; (ii) histological analysis of lung and spleen tissue; and (iii) differential cell counts and cytokine expression in the BALF. Increasing concentrations of intratracheal Con A instillation gradually increased % lung weight per BW compared with the VC. However, BE did not impact % lung weight compared to VC. In fact, increased lung weight indicates a significant increase in pulmonary vascular permeability and inflammatory cell infiltration into damaged lung regions (S15 Fig). This result may be related to the increase in various types of inflammatory cells, including macrophages, neutrophils, and lymphocytes, in the BALF of Con A-instilled groups. In contrast to the lung weight, neither Con A nor BE altered the spleen weight up to doses of 5 mg/kg BW. A slight decrease in the spleen weight of mice was observed at the highest level (10 mg/kg BW) of Con A instillation. Spleen is a source of inflammatory cells that rapidly accumulate at sites of tissue injury. Decreased spleen weight indicates that inflammatory cells in the spleen are rapidly deployed to sites of lung injury.

Additionally, changes in the abundance of different inflammatory cell types were assessed in the BALF after Con A or BE instillation in mice to determine the pulmonary inflammatory response. The results revealed a significant increase in the total number of cells, as well as in the counts of macrophages, eosinophils, and neutrophils in the BALF of Con A-instilled mice as compared to the respective VC group (S16 Fig). However, an increase in cell numbers was not observed with increasing concentration of Con A (1–10 mg/kg BW) instillation. In contrast, the abundance of different cell types did not differ in BE-instilled mice compared to the VC. Moreover, lymphocytes were not observed in the BALF of Con A- or BE-instilled mice. Meanwhile, the spleen weight was also not increased by Con A or BE instillation at the tested concentration levels. An increase in spleen weight could indicate the mitotic transformation of lymphocytes from small to large cells to counteract lectin administration, as previously observed in case of plant lectin toxicity (23). In contrast, lung weight was increased in Con A-instilled mice, which may have been caused in response to water accumulation in the lungs after activated pulmonary neutrophil-induced pulmonary permeability. Alternatively, BE may control neutrophil activation in the lung and reduce ROS production, thereby, reducing pulmonary damage (24).

The pathological changes in organ and inflammatory cells induced in response to lectins resulted in increased expression of inflammatory cytokines (S17 Fig). Proinflammatory Th1, Th2, and Th17 cytokines play important roles in triggering lung inflammatory responses (25–27). Therefore, different cytokines, including IL-1β, IFNγ, and TNFα secreted by Th1, IL-4, IL-5 and IL-13 secreted by Th2, and IL-17 secreted by Th17 cells were measured in the BALF of Con A- or BE-instilled mice. Proinflammatory cytokine levels in BALF were highly increased in the Con A-instilled group (compared to the VC). Th2 cytokine (IL-4, IL-5, and IL-13) levels in the BALF were significantly increased in the Con A-instilled group (compared to the VC); however, the levels of these cytokines appeared to decrease in the Con A-instilled group in a dose-dependent manner. These results are similar to those observed for cellular changes in BAL cells and are more likely to be related to a decrease in eosinophils. However, no change was observed in cytokine levels within the BALF of the BE-instilled group. Consistently, Con A treatment has been reported to induce proinflammatory cytokine expression in human peripheral blood mononuclear cells (28). Meanwhile, griffithsin, which exhibits effects similar to those of BE, does not induce the production of such cytokines (28). Moreover, a range of cytokines were expressed following Con A treatment with the highest expression being of IFNγ followed by that of IL-17, IL-5, IL-13, TNF-a, IL-4, and IL-1β. A previous study showed that IFNγ can induce ROS generation and ferroptosis in tumor cells (29). Thus, Con A treatment may induce ferroptosis by upregulating IFNγ. Moreover, cytokine storms reportedly induce the dysfunction of multiple organs, including the liver, and ultimately can cause death (30). Therefore, high expression of proinflammatory cytokines and pathological changes within organs, such as the liver and lung, following Con A treatment could explain the high mortality observed in these mice. The results further demonstrated that Con A can mimic the conditions observed in autoimmune hepatitis by inducing acute liver injury as indicated by an increase in the levels of hepatic biomarkers and several proinflammatory cytokines (31). Thus, unlike the regular modes of programmed cell death, such as apoptosis and pyroptosis, the observation of cytotoxicity, ROS generation and inflammatory cytokine production, may indicate the occurrence of ferroptosis in Con A-treated cells (32). Indeed, further analysis is required to verify the ferroptosis-related pathological changes in response to Con A treatment.

Effect of Con A and BE on cytotoxicity and ROS generation

Relative cell viability was gradually reduced with increasing concentrations of Con A or BE in all cell lines in a dose-dependent manner (S18 Fig). However, BE induced a lower rate of cytotoxicity. Concordantly, previous studies have shown that Con A is highly cytotoxic, while griffithsin exhibits low cytotoxicity, comparable to that of BE (28). The IC_{20} of Con A in HepG2, MH-S, A549, and Huh7 cells treated for 6 h was determined to be 22.44, 35.95, 38.05, and 50.15 μg/mL, respectively. The IC_{20} of BE for HepG2, MH-S, A549, and Huh7 cells treated for 6 h was determined to be 86·95, 167.69, 110.04, and 232.01 μg/mL, respectively. The higher IC_{20} of BE indicated the lower cytotoxicity of BE (compared to Con A). In addition, different cells lines exhibited different sensitivities to Con A and BE, which may be attributed to the differences in protein expression during exposure to Con A (33). Similar to the cytotoxic effect of Con A, a lower tolerance of HepG2 to the cytotoxic potency of doxorubicin has also been observed previously (33).

Reactive oxygen species (ROS) levels in cells in response to Con A and BE treatment were measured using DCFH- DA staining (34). As observed for the cytotoxic effects, ROS production was observed in a concentration- dependent manner in all cell types, including A549, MH-S, HepG2, and Huh7 cells (S17 Fig). A comparably highand low level of ROS was observed in A549 and Huh7 cells, respectively, in response to Con A treatment. Similarly, a previous study reported that Huh7 cells have a high tolerance to hypoxic conditions (33). Consistentwith the cytotoxicity assay results, BE treatment resulted in remarkably lower ROS production than Con A.

Supplementary Figures

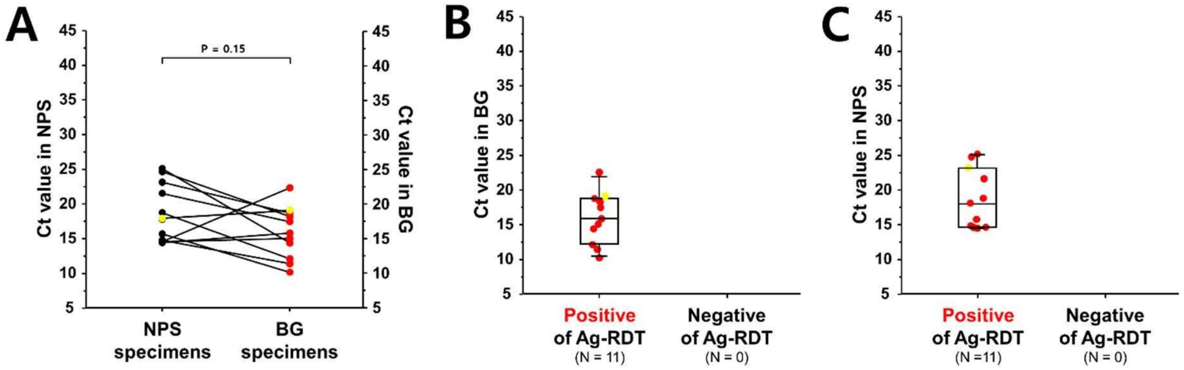

Figure S1. Comparison of Ct values from Beanguard gargle™-based RT-PCR (BG-RT-PCR) with NPS-based RT-PCR (NPS-RT-PCR) analysis in asymptomatic cases. (A) Asymptomatic patients within 6 days of initial COVID-19 confirmation. Among the positive COVID-19 cases within 6 days of illness, the relationship between BG-based antigen rapid detection test results and Ct values of (B) BG-RT-PCR and (C) NPS-RT-PCR with the RdRP gene for asymptomatic patients is shown. Yellow and green circles represent the data points for the Alpha and Delta variants, respectively.

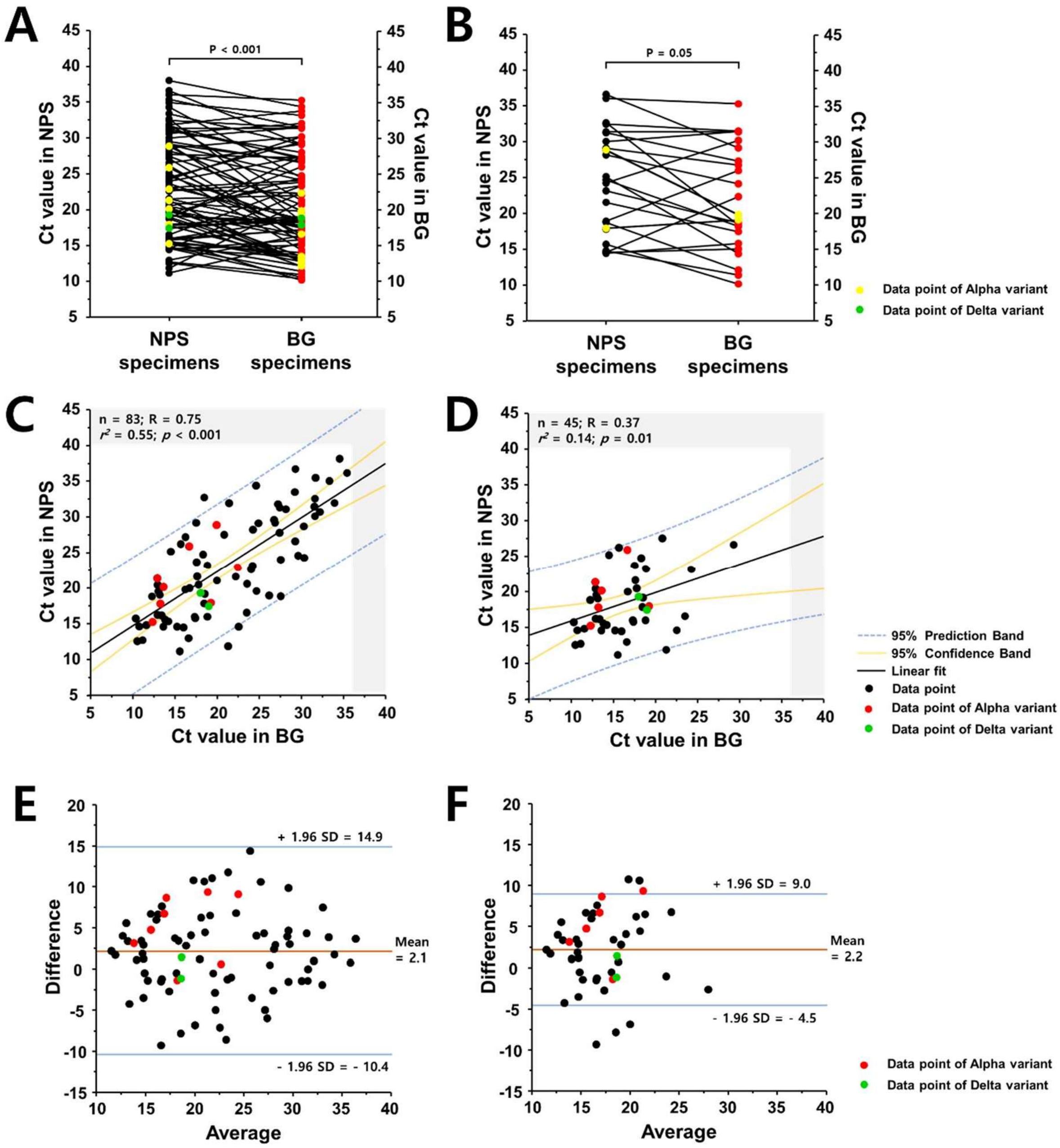

Figure S2. Comparison of the Ct values for BG-RT-PCR with those for NPS-RT-PCR. In the case of all patients (A), the mean and median Ct values, which ranged from 10.1 to 35.3, for Beanguard gargle^{TM} (BG) specimens were 20.8 (95% CI, 19.3–22.3) and 18.8 [IQR, 15.5 to 26.9], respectively, whereas the mean and median Ct values for nasopharyngeal swab (NPS) specimens, which ranged from 11.1 to 39.3, were 22.9 (95% CI, 21.4–24.5) and 21.5 (IQR, 17.0–29.0), respectively. (B) The mean and median Ct values for BG specimens for asymptomatic cases were 22.0 (95% CI, 18.8–25.2) and 19.8 [IQR, 16.6–28.3], respectively, whereas Ct values for NPS specimens were 24.6 (95% CI, 21.4–27.8) and 24.7 [IQR, 18.3–30.7], respectively. Correlation of the C t values for BG-RT-PCR and NPS-RT-PCR with the RdRP gene over the entire period (C) and within 6 days of symptom onset or initial confirmation of COVID-19 (D). Bland-Altman plots for the relationship of Ct values between BG-RT-PCR and NPS-RT-PCR over the entire period (E) and within 6 days of infection (F).

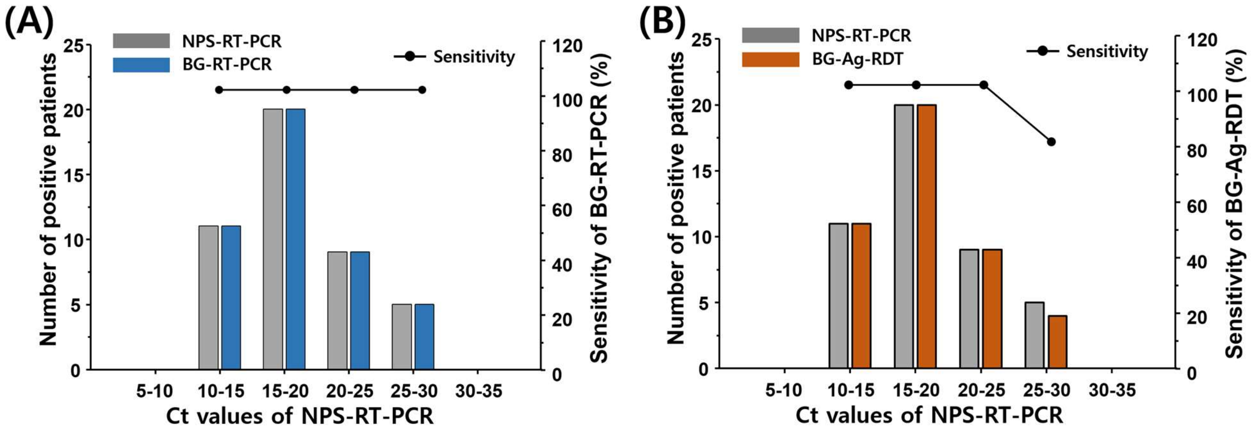

Figure S3. Comparison of the sensitivity of BG-RT-PCR (A) and BG-Ag-RDT (B) depending on the Ct values of NPS-RT-PCR. In the case within 6 days of symptom onset or initial confirmation of COVID-19, the sensitivities of BG-RT-PCR and BG-Ag-RDT were higher than 100% and 80%, respectively, with the increase of Ct values of NPS-RT-PCR.

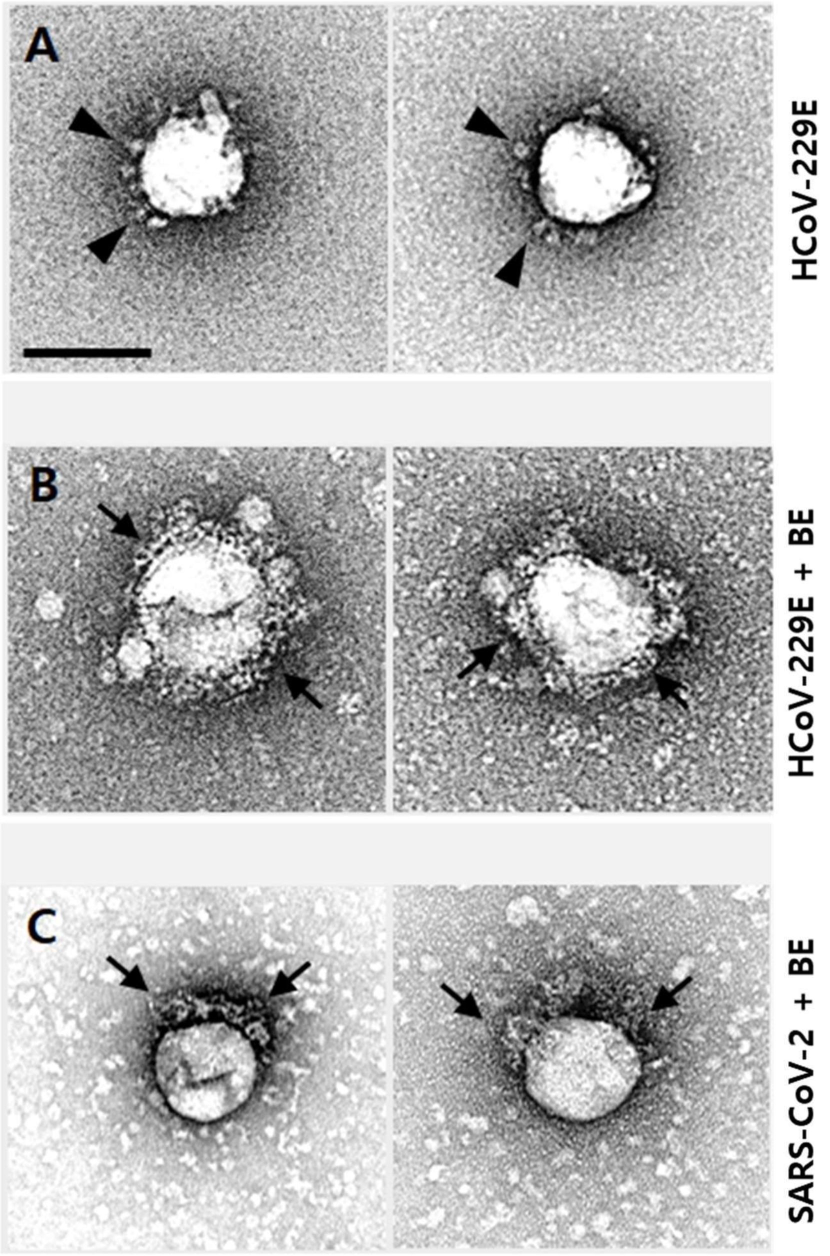

Figure S4. Transmission electron micrographs (TEM) of HCoV-229E and SARS-CoV-2 with bean extract (BE) treatment. TEM images of (A) HCoV-229E before BE treatment, and (B) after treatment and (C) SARS-CoV-2 with BE treatment were obtained. The arrowheads and arrows indicate spikes and BE, respectively. Scale bar = 100 nm.

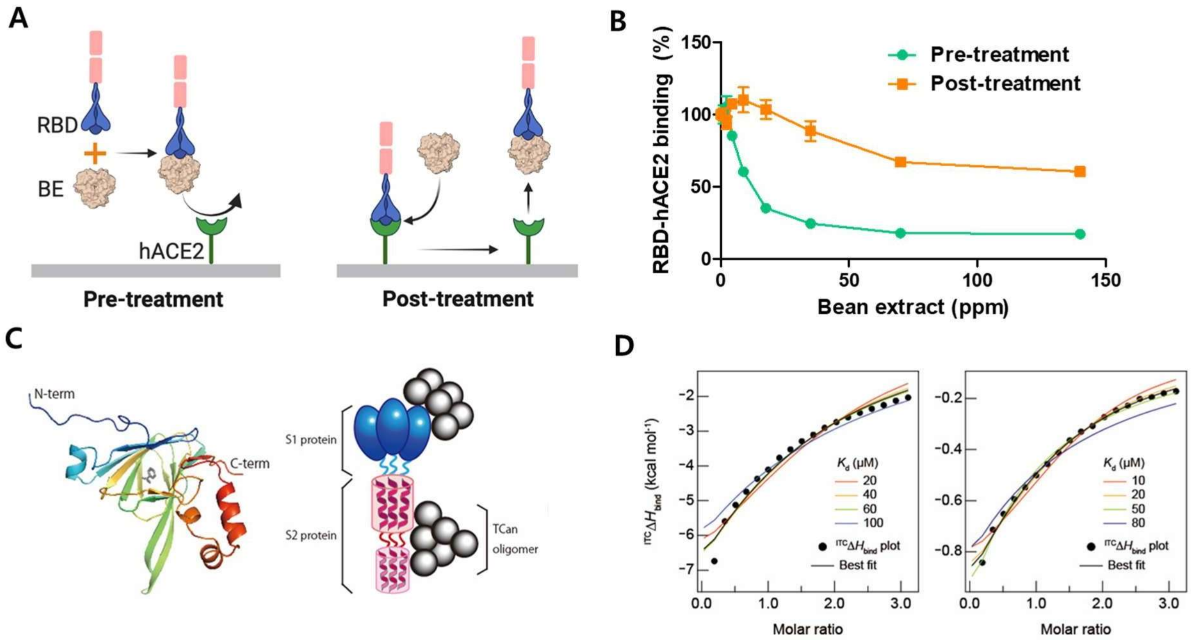

Figure S5. Intermolecular interaction of bean extract (BE) and truncated canavalin (TCan) with SARS-CoV-2. (A) The schematic diagram shows binding inhibition of the recombinant SARS-CoV-2 RBD-Fc-tagged protein to human angiotensin-converting enzyme 2 (hACE2) pre- and post-treatment with BE. The illustrations were created using BioRender. (B) The inhibition of RBD binding to hACE2 receptor was quantified by the difference in absorbance of labeled RBD between the test and control. (C) The three-dimensional structure corresponding to TCan in an intact canavalin generated using a crystal structure of canavalin (PDB ID: 6v7j) (left panel). Binding of TCan oligomers (gray spheres) to S1 (blue ellipses) and S2 proteins (red helical structures) is schematically represented (right panel). (D) Binding isotherms of TCan for S1 (left panel) and S2 (right panel) obtained from isothermal titrationcalorimetry. Colored fit curves in (D) were obtained with a fixed dissociation constant (Kd), and black curves show the best fit.

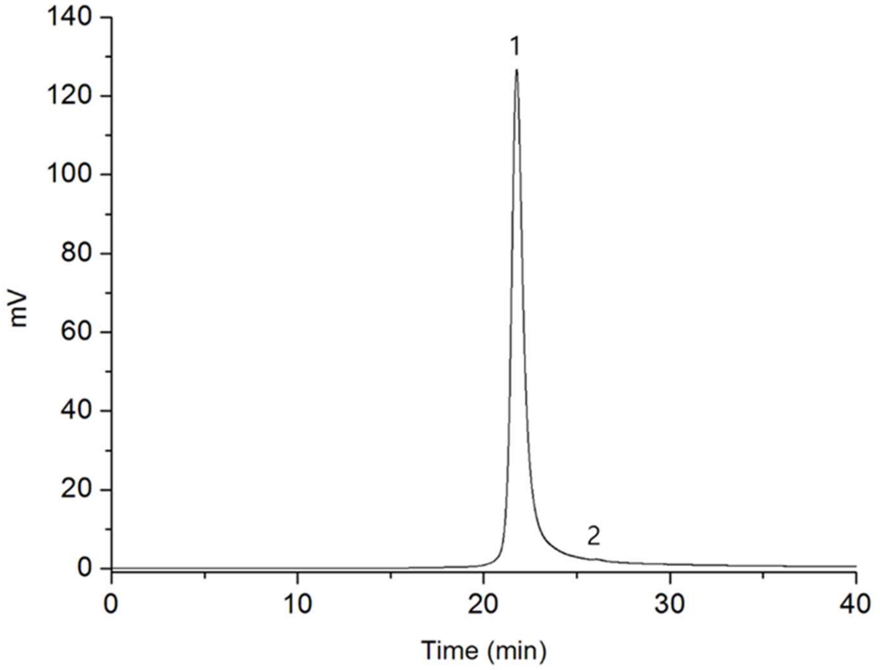

Peak#	Retention Time	Area	Area (%)	Height	Height (%)
1	21.779	6917569	99.91	126173	99.84
2	26.006	6400	0.09	197	0.16

Figure S6. HPLC profile of the purified TCan. The elution profile of the TCan from size-exclusion chromatography in PBS (140 mM NaCl, 2.7 mM KCl, 6.5 mM Na_2HPO_4, 1.5 mM KH_2PO_4, pH7.4) buffer. The calculation of the peak area and height of the protein was performed using LP solution version 1.25 SP4 software (Shimadzu).

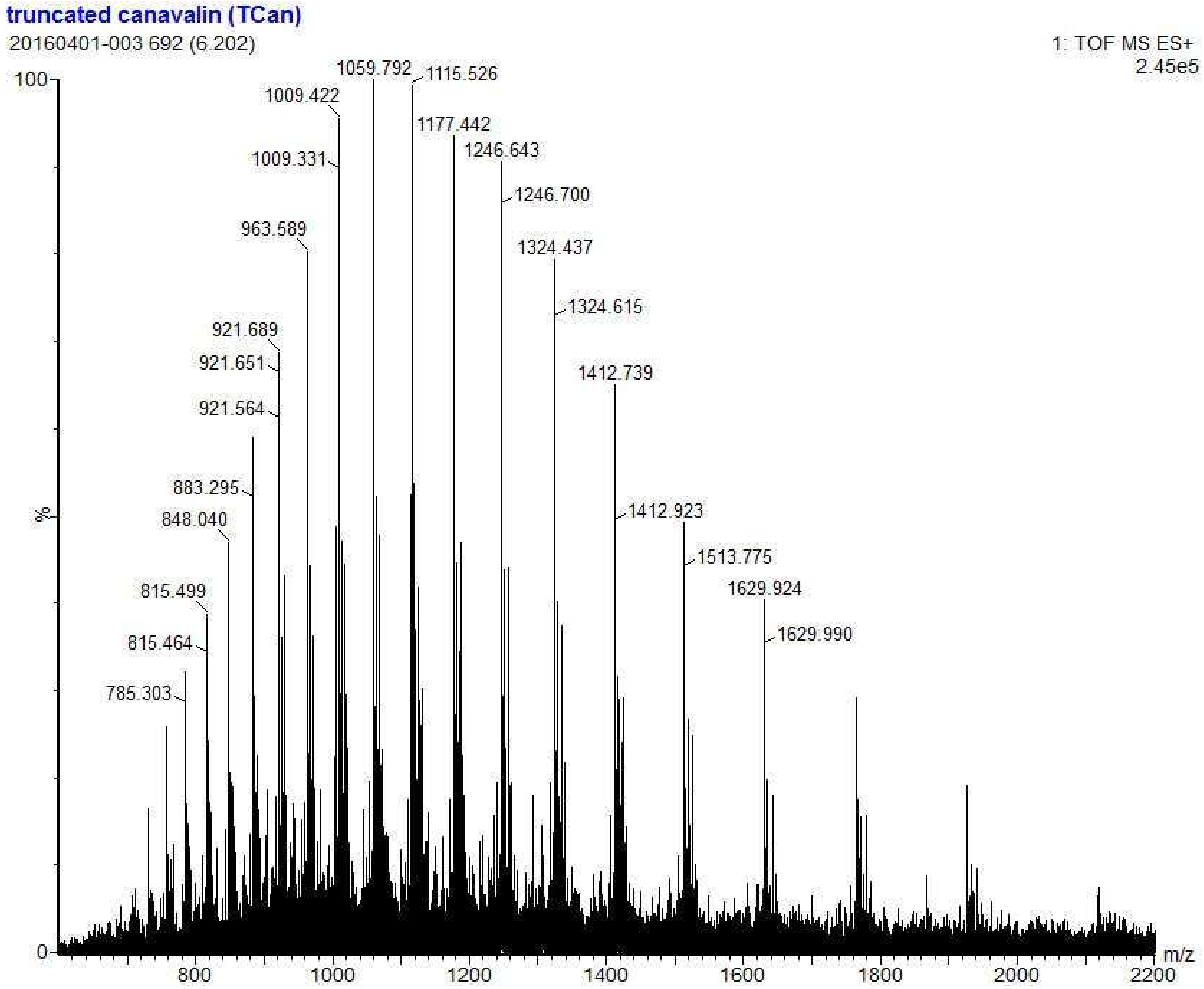

Figure S7. Multiple-charge electrospray mass spectra of TCan. 10 μM of TCan with 0.1% formic acid in dielectric water by ESI-QTOF-mass spectrometry.

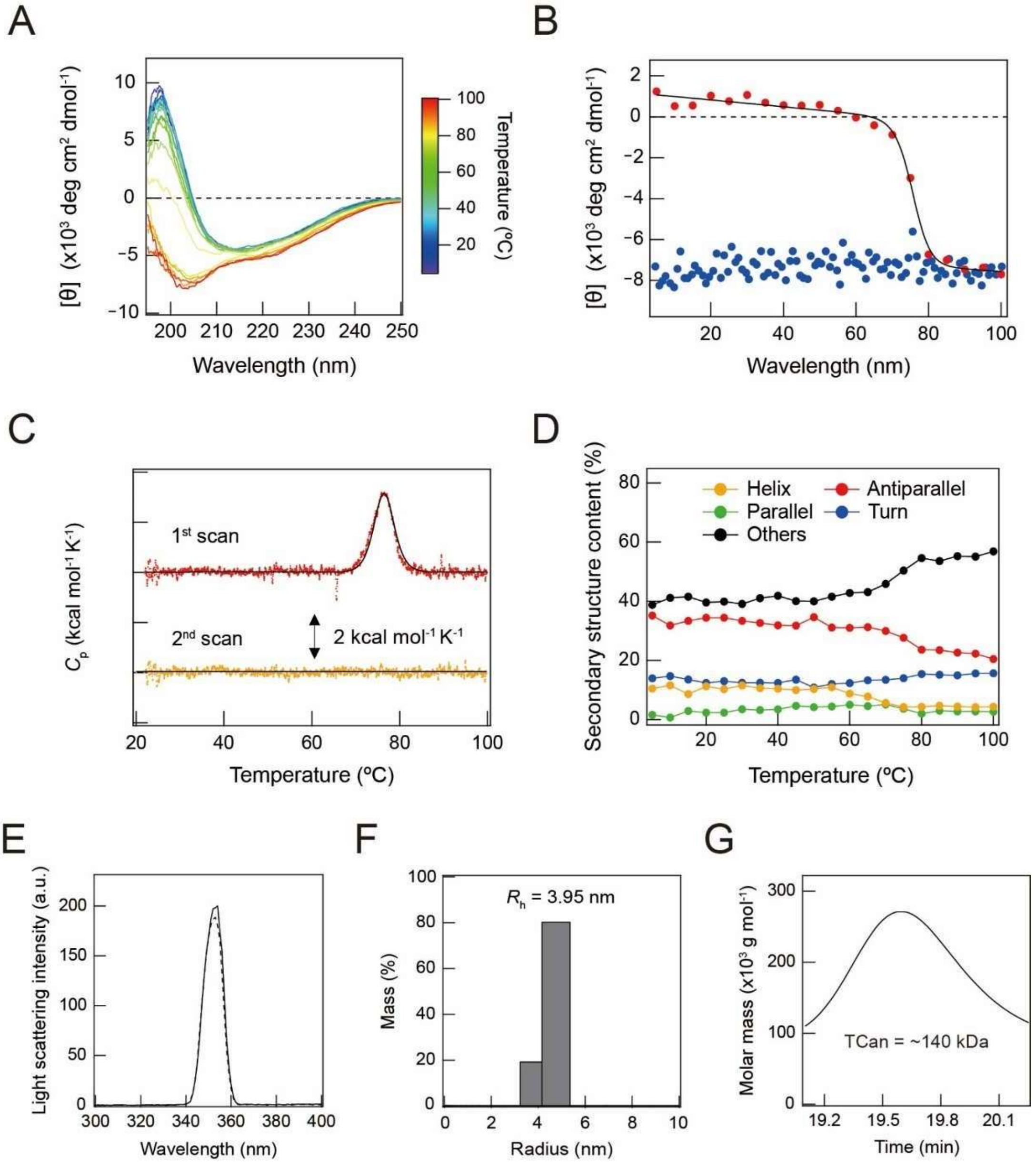

Figure S8. Molecular characterization of TCan using various biophysical approaches. (A–D) Structure and stability of TCan examined by spectroscopy and calorimetry. (A) Far-UV circular dichroism (CD) spectra from 5 to 100 °C are shown with a color code on the right. (B) Heat scanning of CD intensities at 204 nm from 5 to 100 °C (red) and cooling down from 100 to 5 °C (blue). Fit curve is displayed with a continuous line (black). (C)The first (red) and second (yellow) differential scanning calorimetric thermograms of TCan. A continuous line indicates a fit curve. (D) Temperature-dependent changes in the content of the secondary structure of TCan: helical structure (yellow), antiparallel (red) and parallel (green) structure, turn structure (blue), and the other types of structures (black). (E–G) Characterization of oligomeric and colloidal states of TCan. (E) Light scattering spectra of TCan (continuous line) and 20 mM sodium phosphate buffer (dotted line) are shown. (F) Distribution of the hydrodynamic radius (Rh) of TCan obtained by dynamic light scattering before a heat scan. (G) Profile of multi-angle light scattering of TCan before a heat scan.

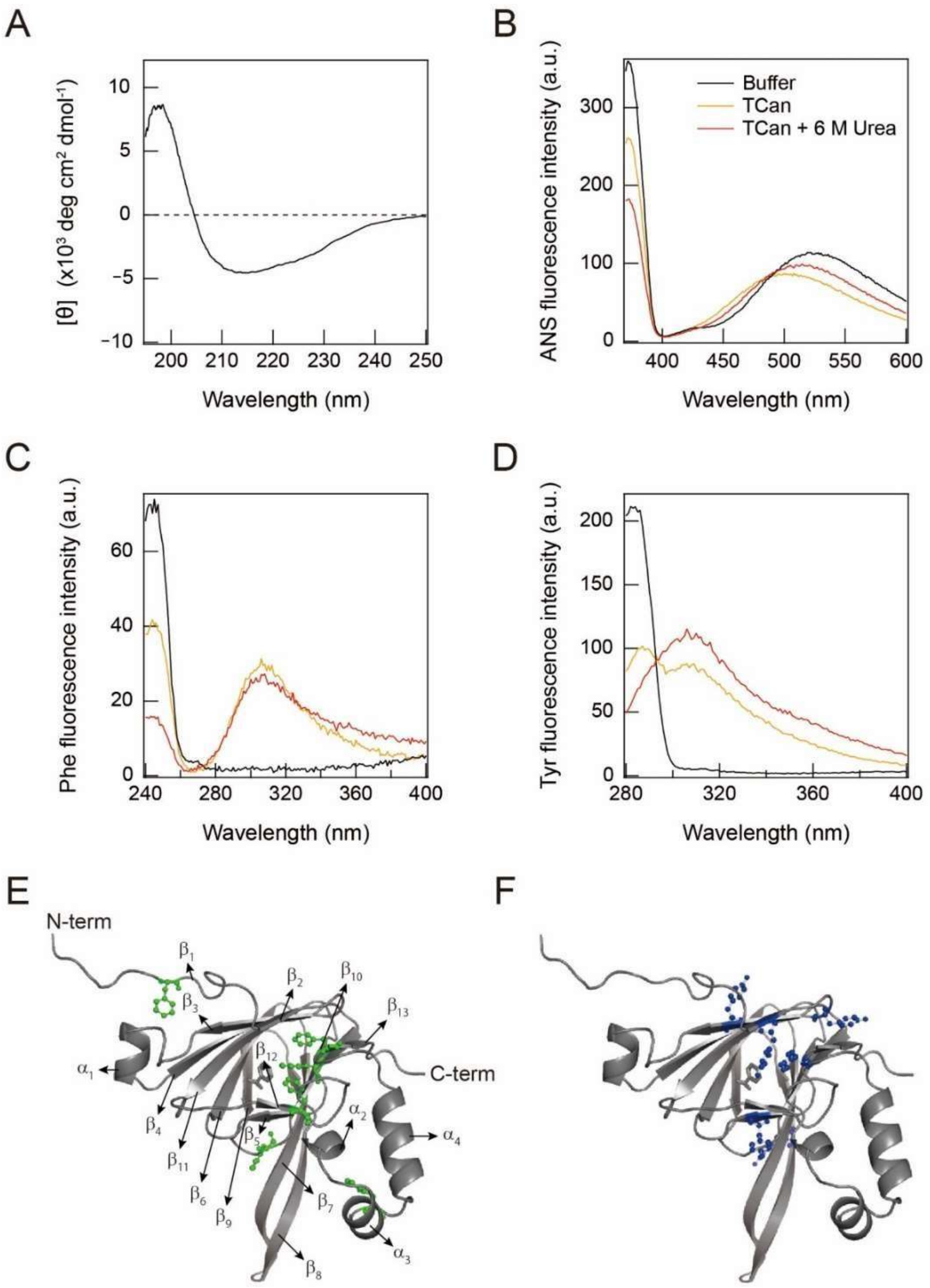

Figure S9. Structural characterization of TCan using spectroscopies. (A) Far-UV circular dichroism spectrum at 25 °C. (B–D) Fluorescence emission spectra of ANS (B), Phe (C), and Tyr (D) of TCan in the absence (orange) and presence (red) of 6 M urea and 20 mM sodium phosphate buffer (black) are shown. (E and F) The three-dimensional structure corresponding to TCan in an intact canavalin generated using a crystal structure of canavalin (PDB ID: 6v7j) (11) are shown. Phe (green) (E) and Tyr (blue) (F) with a ball and stick model and secondary structures are indicated.

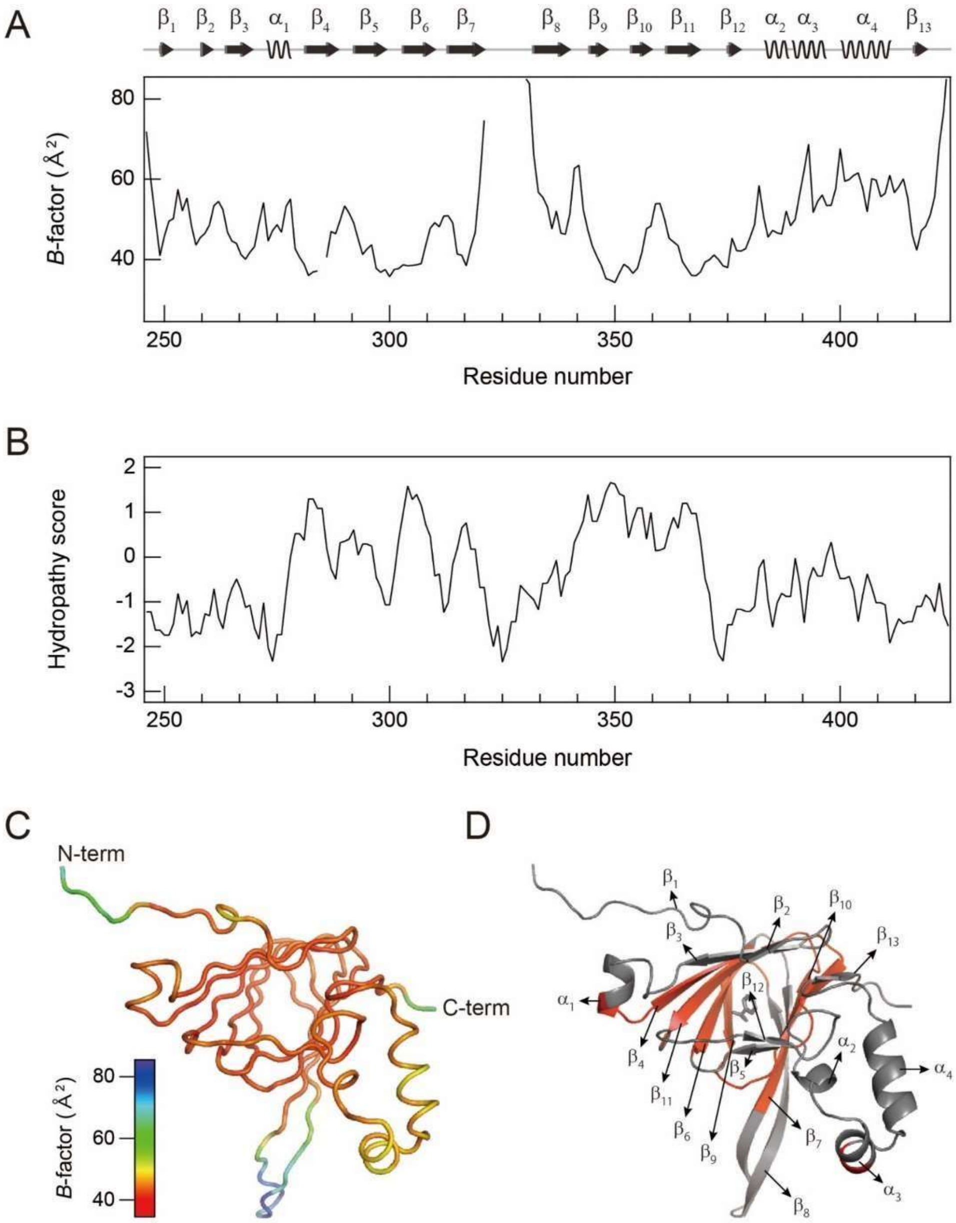

Figure S10. Intramolecular characterization of TCan. (A and B) *B*-factor values (A) and hydropathy scores (B) are plotted against the residue number of TCan. *B*-factors were calculated by averaging *B*-factor values of the main-chain atoms (Cα, N, C', O) in the X-ray structure (PDB ID: 6v7j) (11). Hydropathy scores were estimated based on the scores reported by Kyte and Doolittle (12). The secondary structure elements, α-helix (α) and β-structure (β), are schematically displayed at the top of A. (C) The structure of TCan in an intact canavalin (PDB ID: 6v7j) is color-coded according to the values of *B*-factor as indicated in the scale bar. N- and C-termini are labeled as N-term and C-term, respectively. (D) Hydrophobic regions (in which hydropathy scores are positive) in TCan are colored red. Line and cartoon models of the crystal structure of canavalin (PDB ID: 6v7j) (11) were used in C and D, respectively.

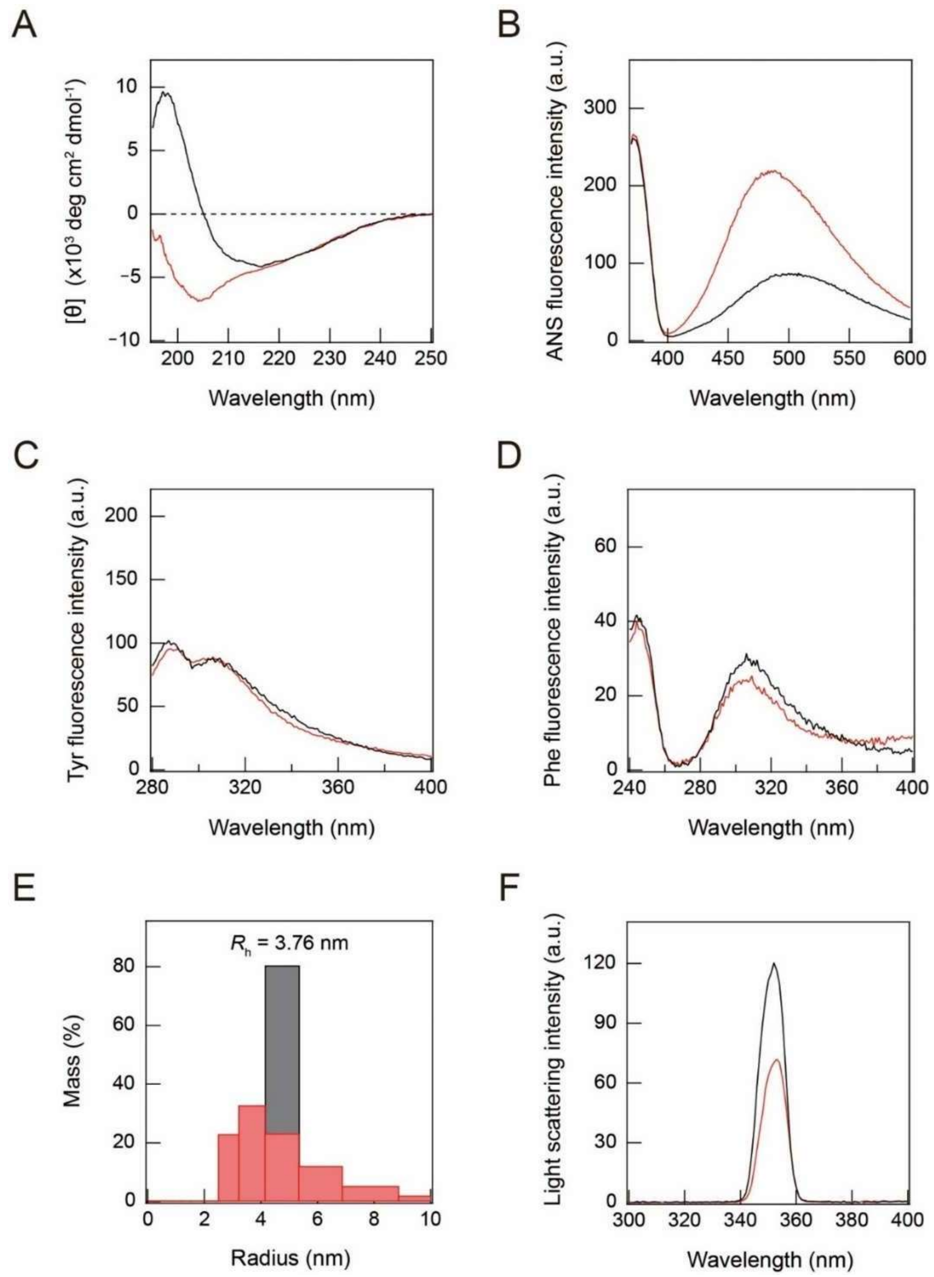

Figure S11. Irreversibility of the unfolding reaction of TCan after heat scanning. (A) Far-UV CD spectra of TCan at 37 °C before (black) and after heat scanning (red). (B–D) Fluorescence emission spectra of ANS (B) as well as Phe (C) and Tyr (D) of TCan before (black) and after the heat treatment (red) are shown. (E and F) Distribution of hydrodynamic radius (R_h) obtained using dynamic light scattering (E) and light scattering spectra (F) before (black) and after the heat treatment (red) are shown.

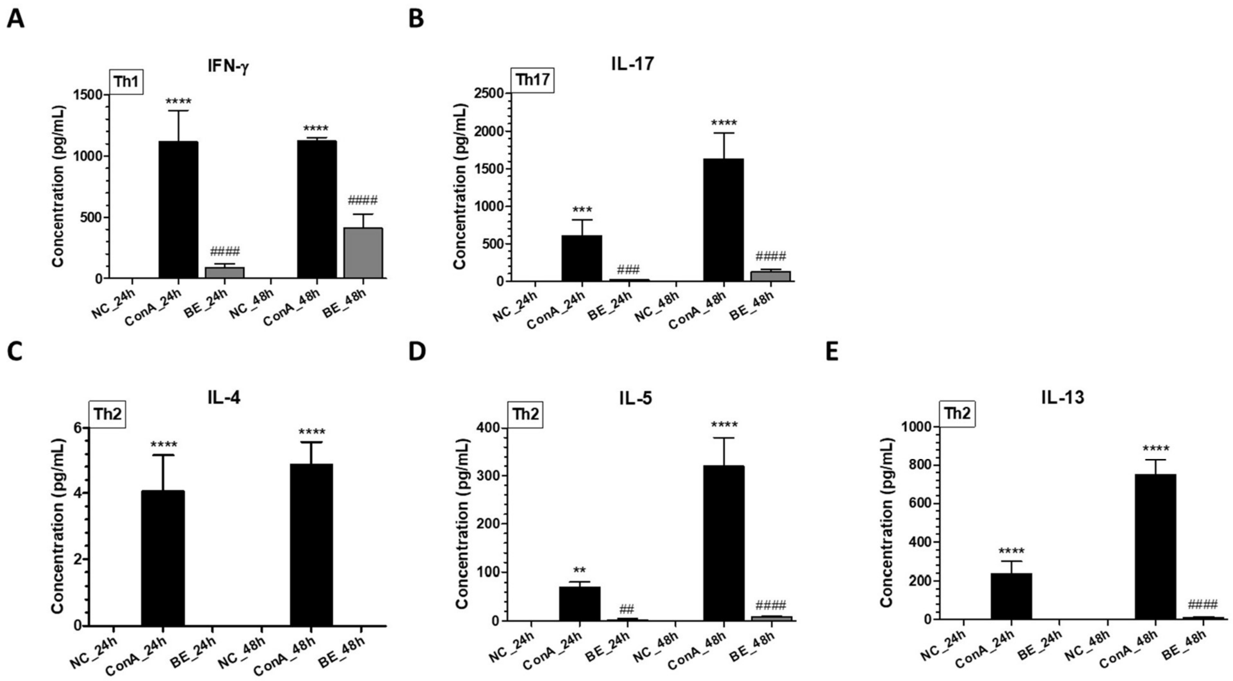

Figure S12. Changes in the expression levels of inflammatory cytokines: (A) interferon gamma (IFN-γ), (B) interleukin (IL)-17, (C) IL-4, (D) IL-5, and (E) IL-13 in splenocytes of Con A- and BE-instilled C57BL/6 mice. Data represent mean ± SD (n = 3 per group). **p < .01 and ****p < .0001 vs. naïve control (NC) group; ##p < .01 and ####p < .0001 vs. Con A group.

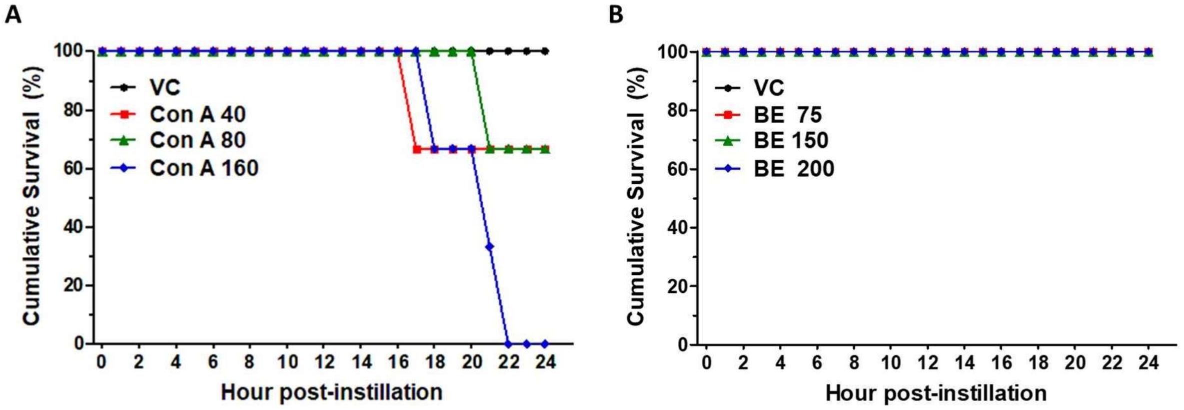

Figure S13. Effect of Con A and BE instillation on survival rate of C57BL/6 mice. (A and B) Survival was recorded every hour up to 24 h after the instillation of Con A at a concentration of 40, 80 or 160 mg/kg body weight (BW), or BE at a concentration of 75, 160, and 200 mg/kg BW, and vehicle control (VC).

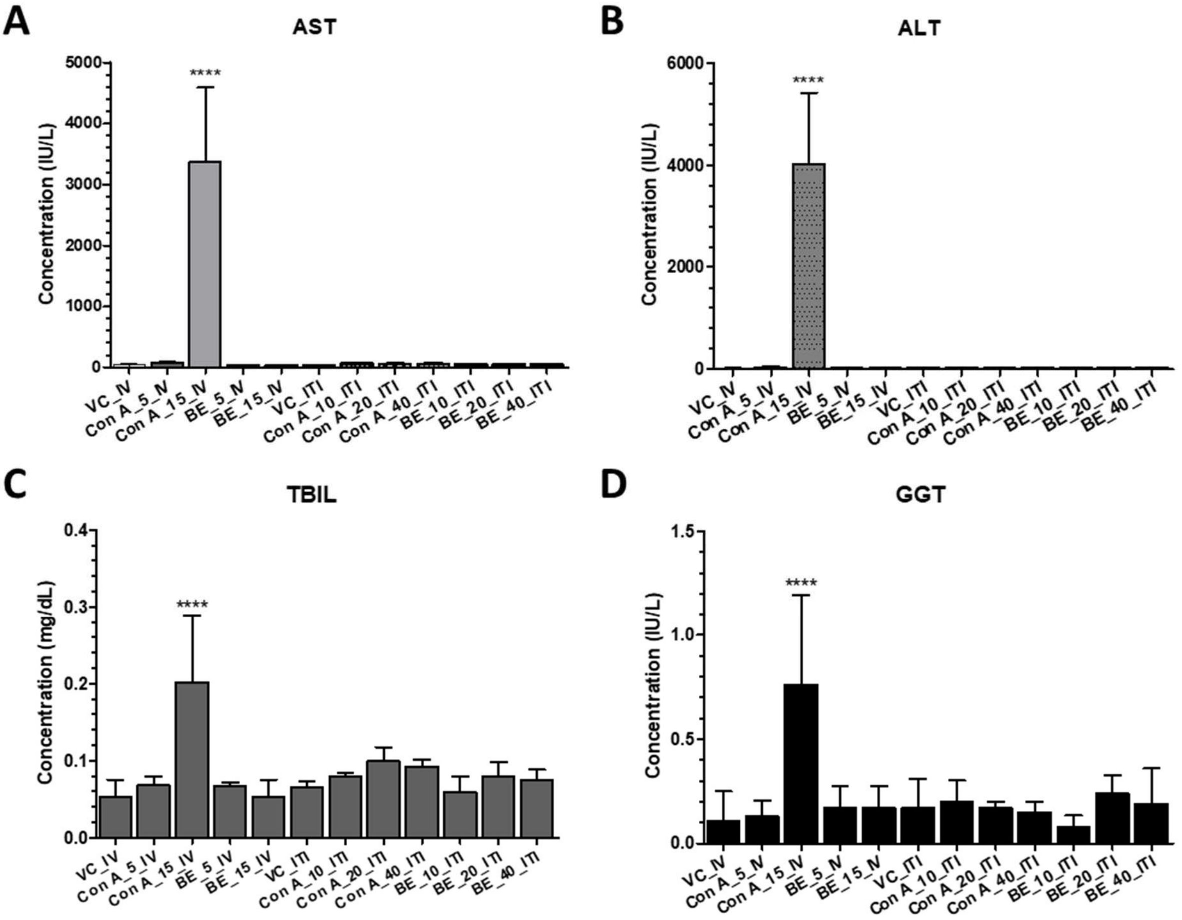

Figure S14. Changes in the levels of hepatic biomarkers (AST, ALT, TBIL, and GGT) in response to Con A and BE instillation in mice. Biomarker concentrations were measured in the blood serum of Con A- and BE-administered mice (intravenously or intratracheal instillation; 5–15 or 10–40 mg/kg BW, respectively). Data represent mean ± SD (n = 5 mice per group). $^{****}p < .0001$ vs. VC. AST, aspartate aminotransferase; ALT, alanine aminotransferase; TBIL, total bilirubin level; GGT, γ-glutamyl transferase.

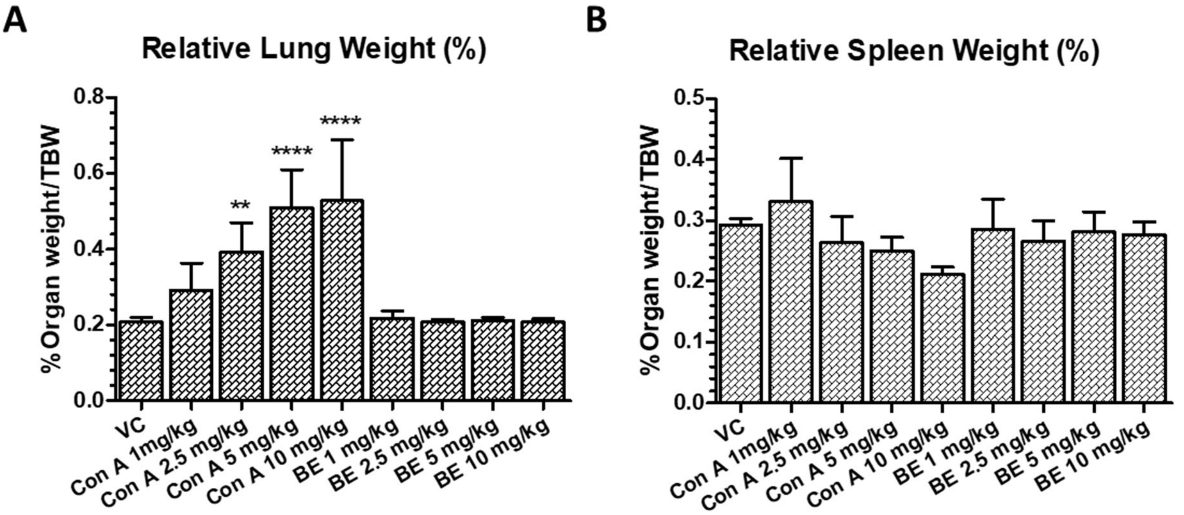

Figure S15. Changes in lung and spleen weight-to-body weight ratio after Con A and BE instillation in mice. (A) Lung and (B) spleen weights at a dose of 1–10 mg/kg body weight (BW) were calculated with the following formula: relative organ weight = organ weight (g)/final body weight (g) × 100%. Data represent mean ± SD (n = 5 per group). *p < .05, **p < .01, ***p < .001 and ****p < .0001 when compared to the corresponding vehicle control (VC), determined using Dunnett's t-test.

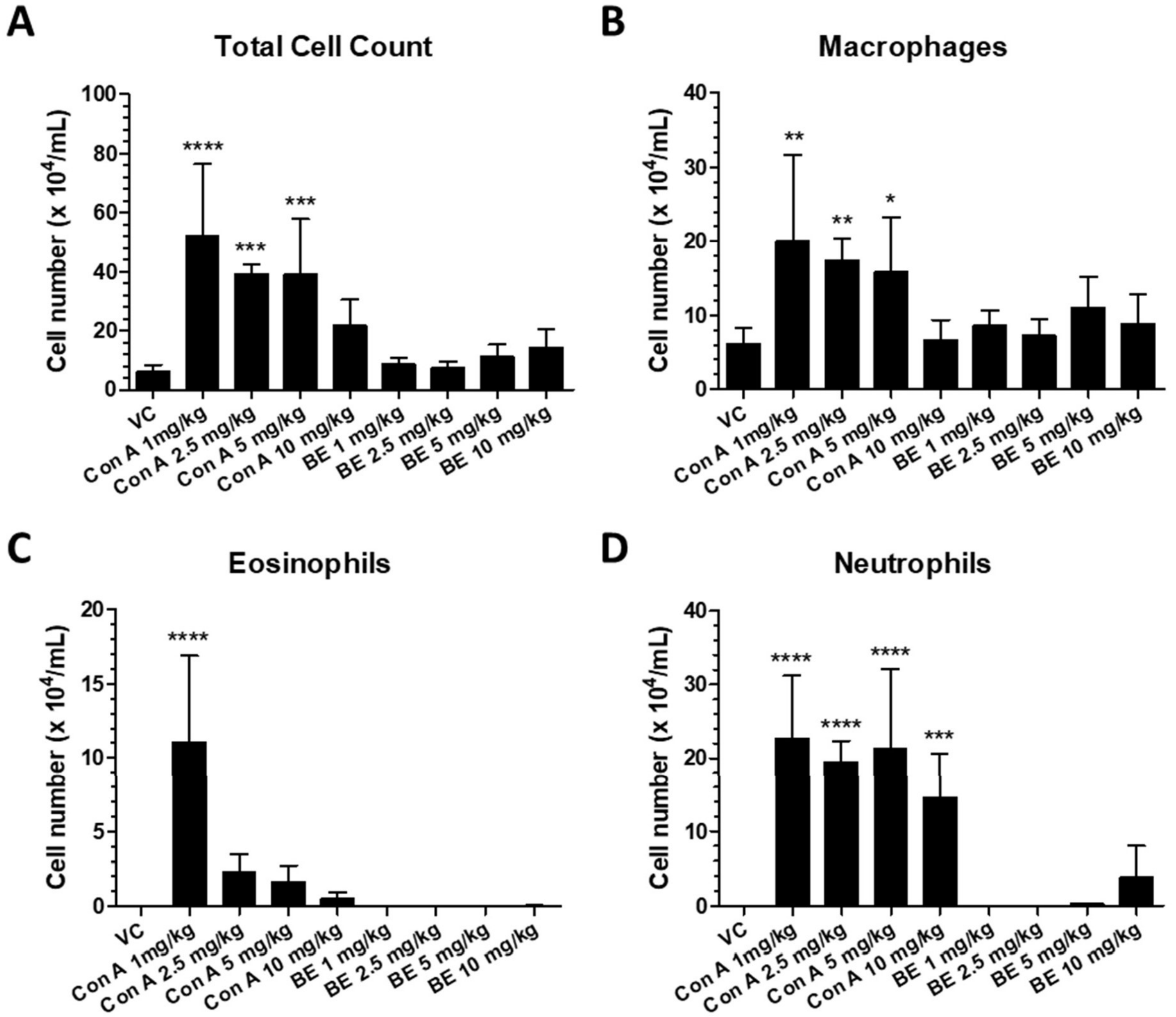

Figure S16. Changes in inflammatory cell numbers in bronchoalveolar lavage fluid (BALF) of mice following Con A and BE instillation. (A) total cells, (B) macrophages, (C) eosinophils, and (D) neutrophils were counted in BALF. BALF was collected and analyzed 24 h after vehicle, Con A, or BE instillation. Data represent mean ± SD (n = 5 per group). *p < .05, **p < .01, ***p < .001 and ****p < .0001 compared to the correspondingvehicle control (VC), determined using Dunnett's t-test.

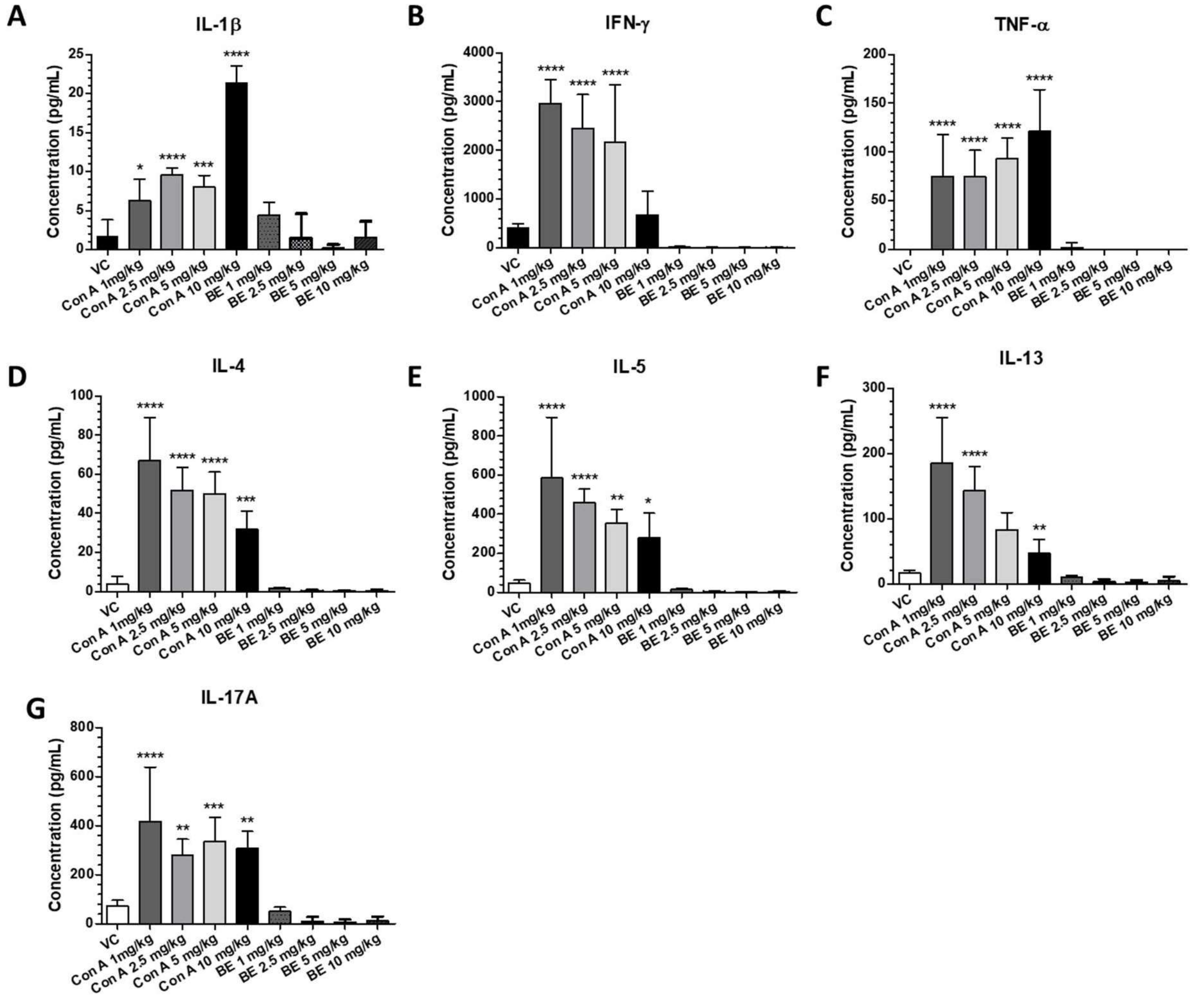

Figure S17. Changes in the expression of inflammatory cytokines. (A) IL-1β, (B) IFN-γ, (C) TNF-α (D), IL-4 (E), IL-5 (F), IL-13, and (G) IL-17A in bronchoalveolar lavage fluid (BALF) after Con A or BE instillation at doses of 1–10 mg/kg BW in mice. Data represent mean ± SD (n = 5 mice per group). *p < .05, **p < .01, ***p < .001 and ****p < .0001 when compared to the corresponding vehicle control (VC), determined using Dunnett's t-test. IL, interleukin, IFN-γ, interferon gamma, TNF-α, tumor necrosis factor alpha.

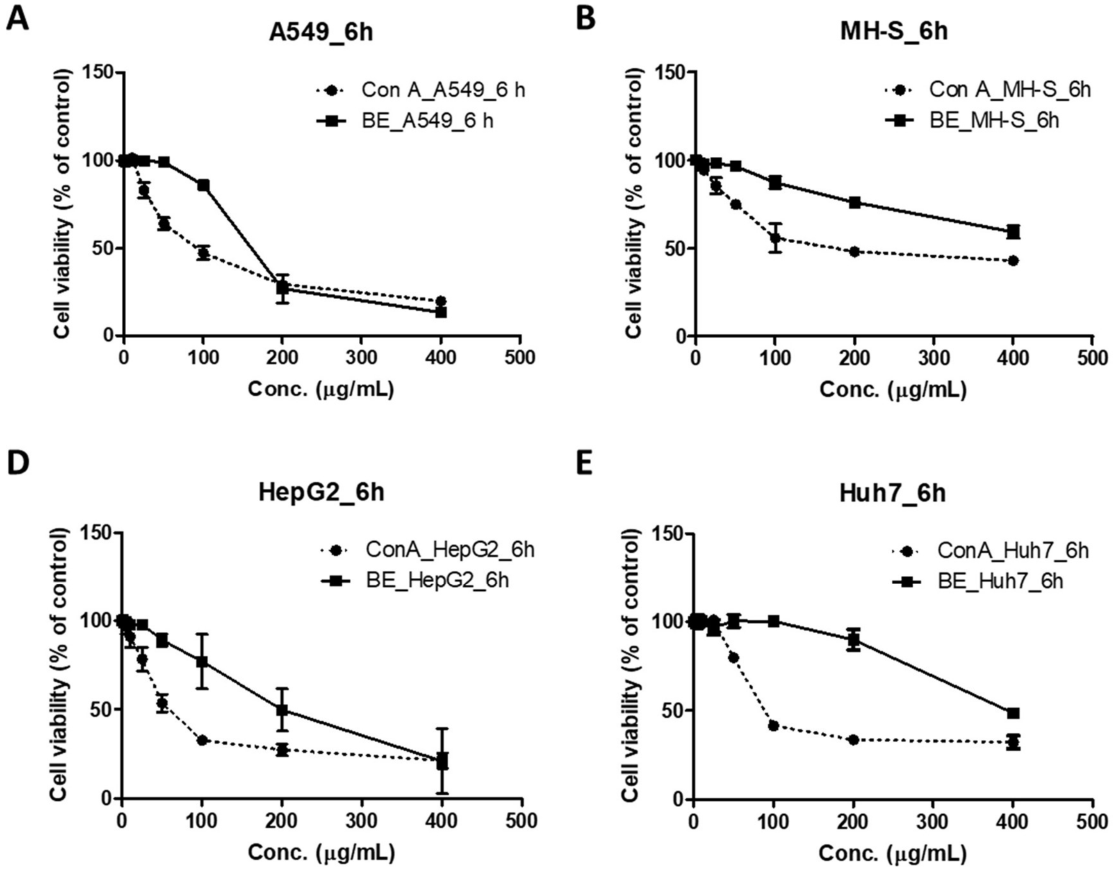

Figure S18. ***In vitro*** **cytotoxicity of Con A and BE**. The toxicity of Con A and BE toward (A) A549, (B) MH-S, (C) HepG2, and (D) Huh7 cells at a concentration of 100–400 μg/mL. Cell viability was measured by counting cells using a NucleoCounter 6 h post infection and is expressed as a percentage of the viability of control cultures. Data represent mean ± SD (n = 3 per group).

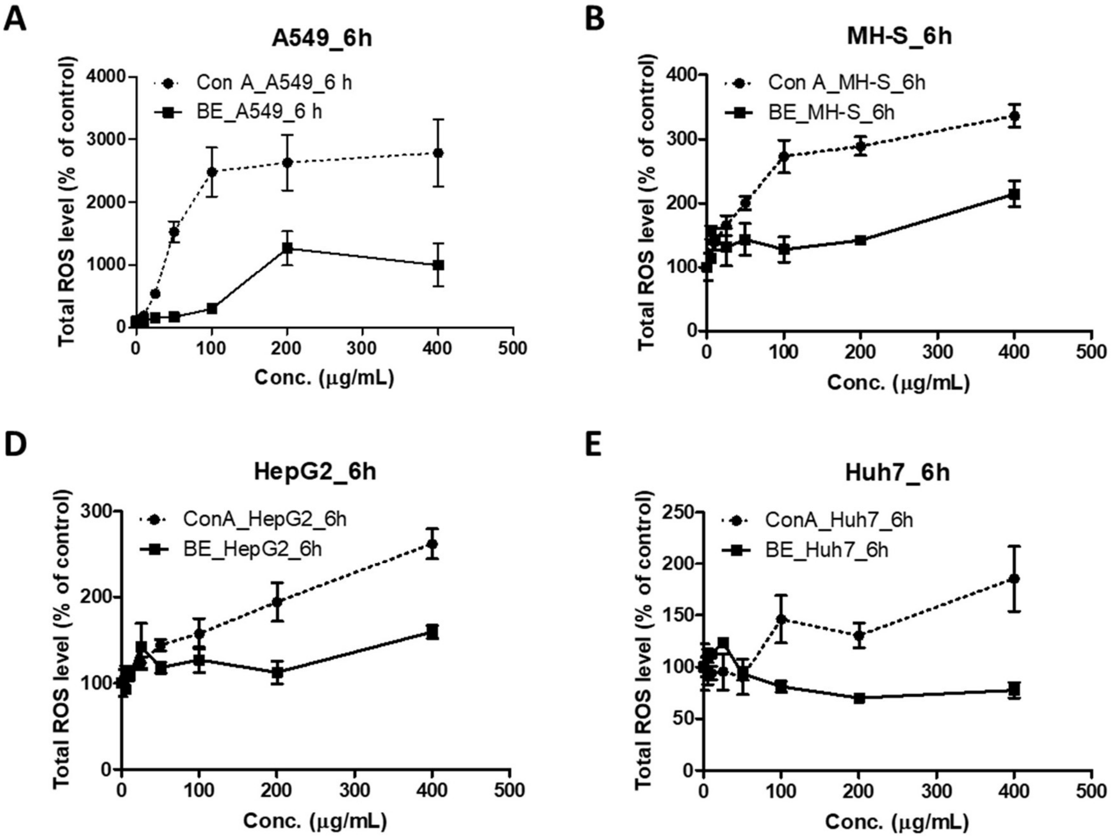

Figure S19. Reactive oxygen species (ROS) level in cells after Con A and BE treatment. ROS generation in (A) A549, (B) MH-S, (C) HepG2, and (D) Huh7 cells after treatment with Con A and BE. ROS level was measured by DCFH-DA staining 6 h after treatment with Con A or BE. The ROS level in cells is expressed as a percentage of untreated controls. Data represent mean ± SD (n = 3 per group).

Supplementary Tables

S1 Table. Evaluation of the diagnostic performance of BG-RT-PCR and BG-Ag-RDTs for patients with COVID-19 throughout the infection duration (n = 102)

Compared to NPS-RT-PCR		COVID-19 patients			Asymptomatic cases		
		Positive	Negative	Total	Positive	Negative	Total
BG-RT-PCR	Positive	83	0	83	23	0	23
	Negative	19	0	19	4	0	4
	Total	102	0	102	27	0	27
Sensitivity (%)		81.4 (95% CI: 72.7–87.7)			85.2 (95% CI: 67.5–94.1)		
Specificity (%)		*N.A.*			*N.A.*		
PPA (%)		100 (95% CI: 95.6–100)			100 (95% CI: 85.7–100)		
NPA (%)		0 (95% CI: 0–16.8)			0 (95% CI: 0–49.0)		
**Compared to BG-RT-PCR**		**Patients (Asymptomatic)[a]**			**Patients (Asymptomatic)[b]**		
		**Positive**	**Negative**	**Total**	**Positive**	**Negative**	**Total**
BG-Ag-RDTs	Positive	54 (13)	0 (0)	54 (13)	53 (13)	0 (0)	53 (13)
	Negative	29 (10)	19 (4)	48 (14)	30 (10)	19 (4)	49 (14)
	Total	83 (23)	19 (4)	102 (27)	83 (23)	19 (4)	102 (27)
Sensitivity (%)		65.1 (56.5)			63.9 (56.6)		
Specificity (%)		100 (100)			100 (100)		
PPA (%)		100 (100)			100 (100)		
NPA (%)		39.6 (28.6)			38.8 (28.6)		
**Compared to NPS-RT-PCR**							
BG-Ag-RDTs	Positive	54 (13)	0 (0)	54 (13)	53 (13)	0 (0)	53 (13)
	Negative	48 (14)	0 (0)	48 (14)	49 (14)	0 (0)	49 (14)
	Total	102 (27)	0 (0)	102 (27)	102 (27)	0 (0)	102 (27)
Sensitivity (%)		52.9 (48.1)			52.0 (48.1)		
Specificity (%)		*N.A.*			*N.A.*		
PPA (%)		100 (100)			100 (100)		
NPA (%)		0 (0)			0 (0)		

N.A.: Not applicable; PPA, positive predictive agreement; NPA, negative predictive agreement; Two saliva-based Ag-RDTs were assessed: [a]Ag-RDTs is STANDARD™ Q COVID-19 Ag Saliva test, and [b]Ag-RDTs is Gmate® COVID-19 Ag Saliva.

S2 Table. Results for the BG-RT-PCR and BG-Ag-RDTs from negative controls (n = 100) and COVID-19 patients within 6 days of illness (n = 45)

		Participants (n = 145)			Asymptomatic case (n = 11)		
		Positive	Negative	Total	Positive	Negative	Total
**BG-RT-PCR compared to NPS-RT-PCR**							
**BG-RT-PCR**	**Positive**	45	0	45	11	0	11
	**Negative**	0	100	100	0	0	0
	**Total**	45	100	145	11	0	11
**BG-Ag-RDT compared to BG-RT-PCR**							
**BG-Ag-RDT[a]**	**Positive**	44	0	44	11	0	11
	**Negative**	1	100	101	0	0	0
	**Total**	45	100	145	11	0	11
**BG-Ag-RDT compared to NPS-RT-PCR**							
**BG-Ag-RDT[a]**	**Positive**	44	0	44	11	0	11
	**Negative**	1	100	101	0	0	0
	**Total**	45	100	145	11	0	11

[a]Two saliva-based Ag-RDTs were assessed: 1) STANDARD™ Q COVID-19 Ag Saliva test, and 2) Gmate® COVID-19 Ag Saliva. There is no difference between the test results of two Ag-RDTs tested for negative controls and COVID-19 patients within 6 days of illness.

S3 Table. Evaluation of the sensitivity of BG-RT-PCR and BG-Ag-RDTs for patients with SARS-CoV-2 Alpha and Delta variants within 6 days of illness

	Type of variant (n)	Sensitivity (%) (95% CI)
**BG-RT-PCR vs. NPS-RT-PCR**	**Alpha (6)**	100 (61.0–100)
	**Delta (2)**	100 (34.2–100)
**BG-Ag-RDT[a] vs. BG-RT-PCR**	**Alpha (6)**	100 (61.0–100)
	**Delta (2)**	100 (34.2–100)
**BG-Ag-RDT[a] vs. NPS-RT-PCR**	**Alpha (6)**	100 (61.0–100)
	**Delta (2)**	100 (34.2–100)

[a]Two saliva-based Ag-RDTs were assessed: 1) STANDARD™ Q COVID-19 Ag Saliva test and 2) Gmate® COVID-19 Ag Saliva. There were no differences between the test results of the two Ag-RDTs regarding the Alpha and Delta variant-positive specimens within 6 days of illness.

S4 Table. Evaluation of the diagnostic performance of BG-RT-PCR and BG-Ag-RDTs for patients of SARS-CoV-2 variants within 6 days of illness

		Alpha variant (n = 6)			Delta variant (n = 2)		
		**Positive**	**Negative**	**Total**	**Positive**	**Negative**	**Total**
**BG-RT-PCR compared to NPS-RT-PCR**							
	**Positive**	6	0	6	2	0	2
**BG-RT-PCR**	**Negative**	0	0	0	0	0	0
	**Total**	6	0	6	2	0	2
**BG-Ag-RDT compared to BG-RT-PCR**							
	**Positive**	6	0	6	2	0	2
**BG-Ag-RDT[a]**	**Negative**	0	0	0	0	0	0
	**Total**	6	0	6	2	0	2
**BG-Ag-RDT compared to NPS-RT-PCR**							
	**Positive**	6	0	6	2	0	2
**BG-Ag-RDT[a]**	**Negative**	0	0	0	0	0	0
	**Total**	6	0	6	2	0	2

[a]Two saliva-based Ag-RDTs were assessed: 1) STANDARD™ Q COVID-19 Ag Saliva test, and 2) Gmate® COVID-19 Ag Saliva. There is no difference between the test results of two Ag-RDTs with the specimens tested positive for Alpha and Delta variants within 6 days of illness.

S5 Table. Sensitivity of BG-Ag-RDTs depending on Ct value of positive BG-RT-PCR samples

		Within 6 days	Entire period
**Ct ≤ 30**	**Positive**	44	54
	**Negative**	1	18
	**Sensitivity (%)**	97.8 (95% CI, 88.4–99.6)	75.0 (95% CI, 63.9–83.6)
**Ct > 30**	**Positive**	0	0
	**Negative**	0	11
	**Sensitivity (%)**	*N.A.*	0 (95% CI, 0–25.9)

N.A.: Not applicable

S6 Table. Summary of thermodynamic parameters for the structural stability of TCan using circular dichroism spectroscopy

$^{CD}T_m$ (℃)	$^{CD}\Delta H$ (kcal/mol)	ΔC_p (kcal/mol K)
**75.4±0.4**	100.2±20·0	2.2±0.3

S7 Table. Summary of thermodynamic parameters for the structural stability of TCan using differential scanning calorimetry

$^{DSC}T_m$ (℃)	$^{DSC}\Delta H_{cal}$ (kcal/mol)	$^{DSC}\Delta H_{VH}$ (kcal/mol)
**76.2±0.0**	19.3±0.1	158.0±0.7

Supplementary References

1. Gasteiger E, Hoogland C, Gattiker A, Duvaud S, Wilkins MR, Appel RD, Bairoch A. Protein Identification and Analysis Tools on the ExPASy Server. In: Walker JM, editor. The Proteomics Protocols Handbook. Totowa: Humana Press; 2005. pp. 571-607.
2. Lu X, Zhu H. 2005. Tube-gel digestion: a novel proteomic approach for high throughput analysis of membrane proteins. Mol Cell Proteomics. 4:1948-1958.
3. Lee S-Y, Yun SH, Lee H, Yi Y-S, Park EC, Kim W, Kim HY, Lee JC, Kim GH, Kim SI. 2020. Analysis of the extracellular proteome of colistin-resistant Korean Acinetobacter baumannii strains. ACS Omega. 5:5713-5720.
4. Greenfield NJ. Using circular dichroism spectra to estimate protein secondary structure. Nat Protoc. 2006;1:2876-2890.
5. Korshavn KJ, Satriano C, Lin Y, Zhang R, Dulchavsky M, Bhunia A, Ivanova MI, Lee YH, La Rosa C, Lim MH, Ramamoorthy A. 2017. Reduced lipid bilayer thickness regulates the aggregation and cytotoxicity of amyloid-β. J Biol Chem. 292:4638-4650.
6. Micsonai A, Wien F, Bulyáki É, Kun J, Moussong É, Lee YH, Goto Y, Réfrégiers M, Kardos J. 2018. BeStSel: a web server for accurate protein secondary structure prediction and fold recognition from the circular dichroism spectra. Nucleic Acids Res. 46:W315-W322.
7. Pal S, Pyne P, Samanta N, Ebbinghaus S, Mitra RK. 2020. Thermal stability modulation of the native and chemically-unfolded state of bovine serum albumin by amino acids. PCCP. 22:179-188.
8. Kim JY, Kinoshita M, Kume S, Gt H, Sugiki T, Ladbury JE, Kojima C, Ikegami T, Kurisu G, Goto Y, Hase T, Lee YH. 2016. Non-covalent forces tune the electron transfer complex between ferredoxin and sulfite reductase to optimize enzymatic activity. Biochem J. 473:3837-3854.
9. Lee YH, Tamura K, Maeda M, Hoshino M, Sakurai K, Takahashi S, Ikegami T, Hase T, Goto Y. 2007. Cores and pH-dependent dynamics of ferredoxin-NADP+ reductase revealed by hydrogen/deuterium exchange. J Biol Chem. 282:5959-5967.
10. Lin Y, Kardos J, Imai M, Ikenoue T, Kinoshita M, Sugiki T, Ishimori K, Goto Y, Lee YH. 2016. Amorphous aggregation of cytochrome c with inherently low amyloidogenicity is characterized by the metastability of supersaturation and the phase diagram. Langmuir. 32:2010-2022.
11. McPherson A. 2020. Binding of benzoic acid and anions within the cupin domains of the vicilin protein canavalin from jack bean (Canavalia ensiformis): Crystal structures. Biochem Biophys Res Commun. 524:268-271.
12. Kyte J, Doolittle RF. 1982. A simple method for displaying the hydropathic character of a protein. J Mol Biol. 57:105-132.
13. Bischof JC, He X. 2005. Thermal stability of proteins. Ann N Y Acad Sci. 1066:12-33.
14. Komiyama T, Miwa M, Yatabe T, Ikeda H. 1984. A circular dichroism study on thermal denaturation of a dimeric globular protein, Streptomyces subtilisin inhibitor. J Biochem. 95:1569-1575.
15. Wilkins DK, Grimshaw SB, Receveur V, Dobson CM, Jones JA, Smith LJ. 1999. Hydrodynamic radii of native and denatured proteins measured by pulse field gradient NMR techniques. Biochemistry. 38:16424-16431.
16. Marky LA, Breslauer KJ. 1987. Calculating thermodynamic data for transitions of any molecularity from equilibrium melting curves. Biopolymers. 26:1601-1620.
17. Gust D, Dirks G. 1981. Stereochemistry of oligomeric proteins. J Theor Biol. 92:39-55.
18. Kinoshita M, Lin Y, Dai I, Okumura M, Markova N, Ladbury JE, Sterpone F, Lee YH. 2018. Energy landscape of polymorphic amyloid generation of beta2-microglobulin revealed by calorimetry. Chem Commun (Camb). 54:7995-7998.
19. Lee YH, Ikegami T, Standley DM, Sakurai K, Hase T, Goto Y. 2011. Binding energetics of ferredoxin-NADP+ reductase with ferredoxin and its relation to function. Chembiochem. 12:2062-2070.
20. Kinoshita M, Kim JY, Kume S, Sakakibara Y, Sugiki T, Kojima C, Kurisu G, Ikegami T, Hase T, Kimata-Ariga Y, Lee YH. 2015. Physicochemical nature of interfaces controlling ferredoxin NADP(+) reductase activity through its interprotein interactions with ferredoxin. Biochim Biophys Acta. 1847:1200-1211.
21. Maw WW, Shimizu T, Sato K, Tomioka H. 1997. Further study on the roles of the effector molecules of immunosuppressive macrophages induced by mycobacterial infection in expression of their suppressor function against mitogen-stimulated T cell proliferation. Clin Exp Immunol. 108:26-33.
22. Gantner F, Leist M, Lohse AW, Germann PG, Tiegs G. 1995. Concanavalin A—induced T-cell—mediated hepatic injury in mice: The role of tumor necrosis factor. Hepatology. 21:190-198.
23. Sharon N, Lis H. 1972. Lectins: Cell-Agglutinating and Sugar-Specific Proteins. Science. 177:949-959.

24. Yang S-C, Chen P-J, Chang S-H, Weng Y-T, Chang F-R, Chang K-Y, Chen CY, Kao TI, Hwang TL. 2018. Luteolin attenuates neutrophilic oxidative stress and inflammatory arthritis by inhibiting Raf1 activity. Biochem Pharmacol. 154:384-396.
25. Branchett WJ, Lloyd CM. 2019. Regulatory cytokine function in the respiratory tract. Mucosal Immunol. 12:589-600.
26. Kubo M. 2017. Innate and adaptive type 2 immunity in lung allergic inflammation. Immunol Rev. 278:162-172.
27. Tan H-L, Rosenthal M. 2013. IL-17 in lung disease: friend or foe? Thorax. 68:788-790.
28. Kouokam J, Huskens D, Schols D, Johannemann A, Riedell S. 2011. Investigation of griffithsin's interactions with human cells confirms its outstanding safety and efficacy profile as a microbicide candidate as a microbicide candidate. PLoS ONE. 6:e22635.
29. Wang W, Green M, Choi JE, Gijón M, Kennedy PD, Johnson JK, Liao P, Lang X, Kryczek I, Sell A, Xia H, Zhou J, Li G, Li J, Li W, Wei S, Vatan L, Zhang H, Szeliga W, Gu W, Liu R, Lawrence TS, Lamb C, Tanno Y, Cieslik M, Stone E, Georgiou G, Chan TA, Chinnaiyan A, Zou W. 2019. CD8+ T cells regulate tumour ferroptosis during cancer immunotherapy. Nature. 569:270-274.
30. Gustot T. 2011. Multiple organ failure in sepsis: prognosis and role of systemic inflammatory response. Curr Opin Crit Care. 17:153-159.
31. Jalan R, Gines P, Olson JC, Mookerjee RP, Moreau R, Garcia-Tsao G, Arroyo V, Kamath PS. 2012. Acute-on chronic liver failure. J Hepatol. 57:1336-1348.
32. Mou Y, Wang J, Wu J, He D, Zhang C, Duan C, et al. 2019. Ferroptosis, a new form of cell death: opportunities and challenges in cancer. J Hematol Oncol. 12:34.
33. Dubbelboer IR, Pavlovic N, Heindryckx F, Sjögren E, Lennernäs H. 2019. Liver cancer cell lines treated with doxorubicin under normoxia and hypoxia: Cell viability and oncologic protein profile. Cancers. 11:1024.
34. Busciglio J, Yankner BA. 1995. Apoptosis and increased generation of reactive oxygen species in Down's syndrome neurons in vitro. Nature. 378:776-779.

STARD Checklist

Section & Topic	No	Item	Reported on page #
**TITLE OR ABSTRACT**			
	**1**	Identification as a study of diagnostic accuracy using at least one measure of accuracy (such as sensitivity, specificity, predictive values, or AUC)	1, 3
**ABSTRACT**			
	**2**	Structured summary of study design, methods, results, and conclusions (for specific guidance, see STARD for Abstracts)	3
**INTRODUCTION**			
	**3**	Scientific and clinical background, including the intended use and clinical role of the index test	5-6
	**4**	Study objectives and hypotheses	6
**METHODS**			
Study design	**5**	Whether data collection was planned before the index test and reference standard were performed (prospective study) or after (retrospective study)	7
Participants	**6**	Eligibility criteria	5 in Supplemental material 2
	**7**	On what basis potentially eligible participants were identified (such as symptoms, results from previous tests, inclusion in registry)	8, Supplemental material 1
	**8**	Where and when potentially eligible participants were identified (setting, location and dates)	8, Supplemental material 1
	**9**	Whether participants formed a consecutive, random or convenience series	6, Supplemental material 1
Test methods	**10a**	Index test, in sufficient detail to allow replication	8-9
	**10b**	Reference standard, in sufficient detail to allow replication	8-9
	**11**	Rationale for choosing the reference standard (if alternatives exist)	8-9
	**12a**	Definition of and rationale for test positivity cut-offs or result categories of the index test, distinguishing pre-specified from exploratory	8-9
	**12b**	Definition of and rationale for test positivity cut-offs or result categories of the reference standard, distinguishing pre-specified from exploratory	8-9
	**13a**	Whether clinical information and reference standard results were available to the performers/readers of the index test	8-9, Supplemental material 1
	**13b**	Whether clinical information and index test results were available to the assessors of the reference standard	8-9, Supplemental material 1
Analysis	**14**	Methods for estimating or comparing measures of diagnostic accuracy	8-10
	**15**	How indeterminate index test or reference standard results were handled	8-10
	**16**	How missing data on the index test and reference standard were handled	8-10, Figure 1
	**17**	Any analyses of variability in diagnostic accuracy, distinguishing pre-specified from exploratory	7-9
	**18**	Intended sample size and how it was determined	9
**RESULTS**			
Participants	**19**	Flow of participants, using a diagram	Figure 1, 4 in Supplemental material 2
	**20**	Baseline demographic and clinical characteristics of participants	Table 1
	**21a**	Distribution of severity of disease in those with the target condition	11, Supplemental material 1
	**21b**	Distribution of alternative diagnoses in those without the target condition	N. A.
	**22**	Time interval and any clinical interventions between index test and reference standard	7-8
Test results	**23**	Cross tabulation of the index test results (or their distribution) by the results of the reference standard	Figure 2 and S1-2, Table 2 and S1-5
	**24**	Estimates of diagnostic accuracy and their precision (such as 95% confidence intervals)	11-13 Table 2 and S1-5
	**25**	Any adverse events from performing the index test or the reference standard	N. A.

**DISCUSSION**			
	**26**	Study limitations, including sources of potential bias, statistical uncertainty, and generalisability	18-19
	**27**	Implications for practice, including the intended use and clinical role of the index test	3, 6, 17-19
**OTHER INFORMATION**			
	**28**	Registration number and name of registry	3
	**29**	Where the full study protocol can be accessed	Supplemental material 2
	**30**	Sources of funding and other support; role of funders	20
